# Interactions of LINE-1 ORF1p with proteins and chromatin suggest a role in neuronal physiology

**DOI:** 10.26508/lsa.202503546

**Published:** 2026-08-21

**Authors:** Sandra Sinnassamy, Olivia Massiani Beaudoin, Berangère Lombard, Damarys Loew, Tom Bonnifet, Magali Fradet, Héloïse Monnet, Thomas Caille, Nicolas Servant, Rajiv L Joshi, Julia Fuchs

**Affiliations:** 1 CIRB, Collège de France, Université PSL, INSERM, CNRS , Paris, France; 2 Institut Curie, Université PSL, CurieCoreTech Mass Spectrometry Proteomics , Paris, France; 3 Orion Technological Core, CIRB, Collège de France, Université PSL, INSERM, CNRS , Paris, France; 4 Institut Curie, INSERM U900, Mines Paris Tech, Université PSL , Paris, France

## Abstract

Retrotransposons are emerging as novel regulators of embryonic and brain development. We recently demonstrated that the LINE-1–encoded protein ORF1p is abundantly expressed in adult mouse and human neurons, although its function remains unclear. Here, we characterize the ORF1p interactome in differentiated mouse and human neurons using mass spectrometry and identify novel partners implicated in gene regulation and neuron-specific processes. ORF1p localizes not only to neuronal nuclei, where it associates with chromatin under steady-state conditions, but also to neurites, supporting a role in neuronal physiology. To further explore its nuclear functions, we sorted human post-mortem neurons with high or low nuclear ORF1p levels and performed ORF1p knockdown in cultured human neurons, followed by chromatin accessibility assays. Both approaches revealed consistent patterns of differential chromatin accessibility dependent on ORF1p. Loss of ORF1p also led to the down-regulation of long, neuron-specific genes and altered neurite morphology. Together, these findings point to a physiological role of ORF1p in post-mitotic neurons, mediated through converging interactions with proteins and chromatin.

## Abstract (precis)

ORF1p, a LINE-1-encoded protein, regulates chromatin accessibility, neuron-specific gene expression, and neurite morphology in post-mitotic neurons, revealing a physiological role for this retrotransposon protein in the brain.

## Introduction

Transposable elements (TEs), once considered junk DNA, are gaining interest as physiological modulators of genome architecture (1) and early embryonic (2) as well as neurodevelopment (3) but also pathological drivers of aging-related (4) and neurogenerative processes. Half of the human genome is composed of TEs, and 17% is of the Long Interspersed Nuclear Element-1 (LINE-1 or L1) (5) TE family. Between 100 and 150 LINE-1 (6, 7) are considered full-length L1 elements (flLINE-1). They are the only autonomous mobile elements in the human genome and are characterized by a bidirectional RNA polymerase II promoter in the 5′UTR and intact open reading frames encoding the canonical L1 proteins, ORF1p and ORF2p, essential for LINE-1 mobilization. ORF1p and ORF2p, translated from a bicistronic mRNA, associate with *cis-preference* to the LINE-1 mRNA to form a ribonucleoprotein (RNP). Once the LINE-1 RNP gets access to the nucleus, a new copy is pasted into the genome through target-primed reverse transcription. ORF1p is an RNA-binding protein (RBP) and a nucleic acid chaperon, and ORF2p has endonuclease and reverse transcriptase activities. When abnormally expressed, LINE-1 may represent a threat to genome and cellular integrity by generating DNA double-strand breaks and triggering inflammatory pathways (8). Consequently, LINE-1 expression is rigorously controlled through multiple mechanisms at the transcriptional and post-transcriptional levels (9). Nevertheless, LINE-1 has been shown to exert physiological functions, especially during early embryonic (10, 11) and neuronal development (3, 12, 13, 14). LINE-1 retrotransposition was observed in the human brain and neuronal precursor cells (15, 16). Intriguingly, recent and optimized next-generation sequencing data have highlighted the abundant expression of LINE-1 in the adult human brain (17, 18), revealing significant enrichment of LINE-1 expression, including evolutionary young L1HS and L1PA2 elements, in adult human neurons with no or limited expression of LINE-1 in non-neuronal cells at steady state (12, 17, 18). Indeed, on the protein level, LINE-1 encoded ORF1p expression was observed predominantly in neurons throughout the adult mouse brain (19) and in several regions of the human brain (19, 20) as well as in human dopaminergic neurons (19, 20, 21). The presence of ORF1p in adult neurons is a surprising observation given the deleterious consequences to cellular integrity attributed to LINE-1 expression and led us to test the hypothesis of a physiological function of this protein in the adult brain. To this end, we used a combination of mass spectrometry, chromatin accessibility assays, and transcriptomics. We found an important protein interaction network of endogenous ORF1p in adult neurons encompassing proteins related to RNA metabolism, as expected for an RBP, but also proteins related to nuclear functions, including chromatin regulation and gene expression as well as proteins with neuron-specific functions. This finding and the presence of ORF1p in chromatin fractions led us to analyse chromatin accessibility in nuclei with high or low ORF1p content using sorted *post-mortem* neurons or LINE-1 knockdown (LINE1-KD) in differentiated human neurons in which we also assessed transcriptomic changes. Characteristics of differentially accessible regions (DARs) were significantly different between nuclei with high or low ORF1p content, and these differences were highly similar across two different experimental systems involving gene loci related to genes important for neuronal functions. LINE1-KD led to the down-regulation of particularly long, protein-coding genes enriched in neuronal functions. Functionally, we observed morphological changes in neurites of adult mouse neurons upon LINE1-KD. Together, these data suggest a role of ORF1p in the maintenance and integrity of neuronal function in adult neurons via converging actions on chromatin organization, gene transcription, and protein interactions.

## Results

### Identification of protein partners of endogenous ORF1p in mouse and human neurons

**Figure 1.**
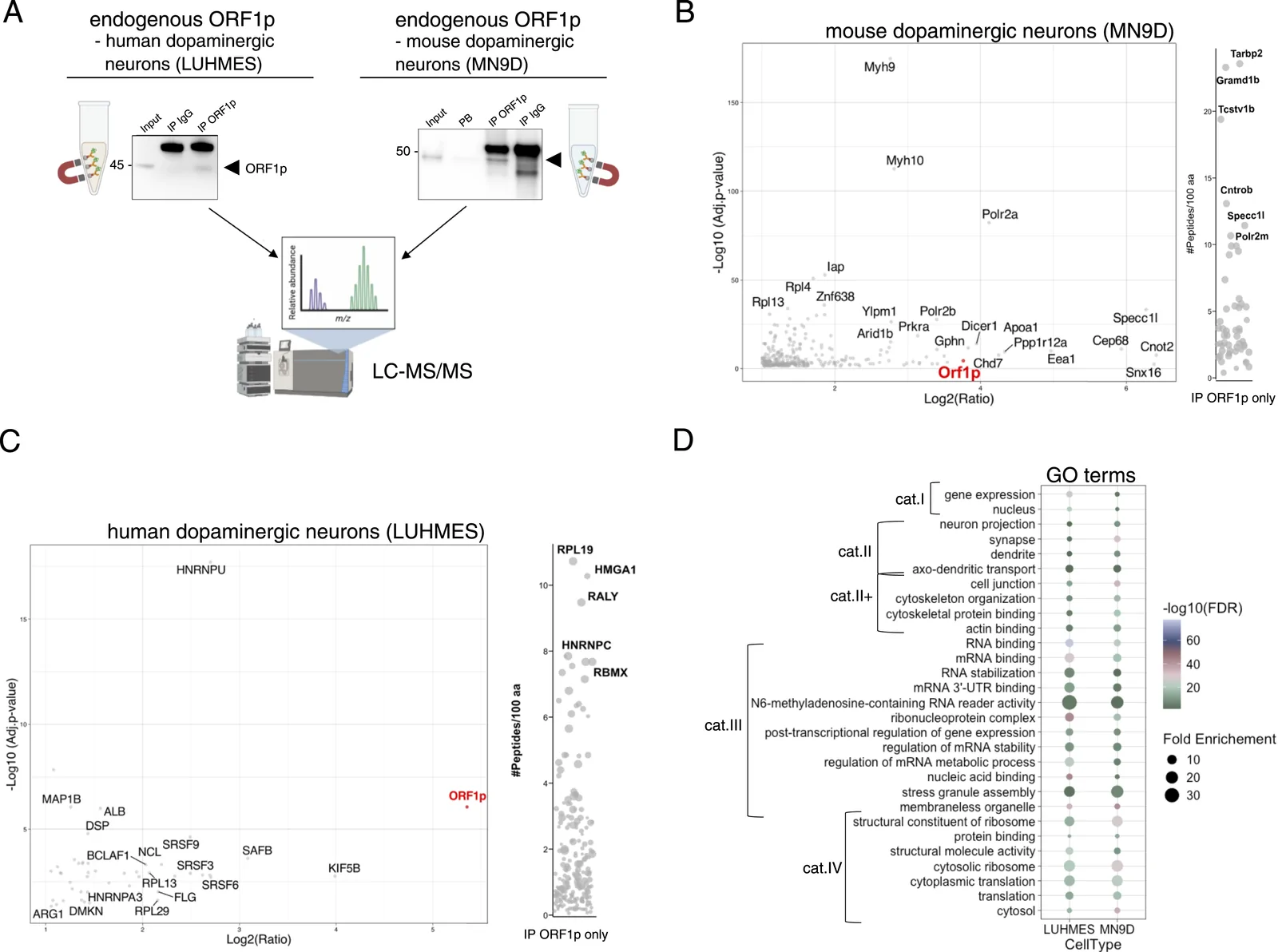
Quantitative mass spectrometry analysis of protein partners of endogenous ORF1p in human and mouse dopaminergic neurons. **(A)** Graphical representation of the experimental protocol used to identify protein partners of endogenous ORF1p in mouse and human neurons. Endogenous ORF1p was immunoprecipitated (IP) from human (LUHMES) and mouse (MN9D) differentiated dopaminergic neurons, respectively. Enrichment was verified by Western blot analysis, and lysates were analysed by LC-MS/MS, followed by the identification and quantification of protein partners. **(B)** Specific protein partners of ORF1p found by proteomic mass spectrometry in MN9D neurons, presented according to their fold change (ratio IP-ORF1p/IP-IgG) and adjusted *P*-value. Proteins identified in the IP ORF1p (ab216324) by LC-MS/MS were compared with IP IgG. For each condition (IP IgG, IP ORF1p), five independent IPs were performed. Proteins with at least two distinct proteotypic peptides in all five replicates in the IP ORF1p condition with a ratio of IP ORF1p/IP IgG ≥2 and a *P*-value ≤0.05 were considered ORF1p-interacting proteins. In the right panel, partners of ORF1p, which were not detected in the IP IgG condition (and therefore have no fold change or *P*-value), are shown according to the number of peptides per 100 amino acids identified. **(C)** Specific protein partners of ORF1p identified by proteomic mass spectrometry in LUHMES neurons, presented according to their fold change (ratio) and adjusted *P*-value. For each condition (IP IgG, IP ORF1p), five independent IPs were performed. Proteins identified in the IP ORF1p (ab49) by LC-MS/MS were compared with IP IgG. Proteins with at least one distinct proteotypic peptide in at least three replicates identified in the IP ORF1p condition with a fold change ≥2 and *P*-value ≤0.05 were considered ORF1p-interacting proteins. In the right panel, partners of ORF1p, which were not detected in the IP IgG condition (and therefore have no fold-change or *P*-value), are shown according to their number of peptides per 100 amino acids identified. **(D)** Top common GO categories (CC, MF, BP) of ORF1p protein partners in mouse (MN9D) and human (LUHMES) dopaminergic neurons.

**Figure S1.**
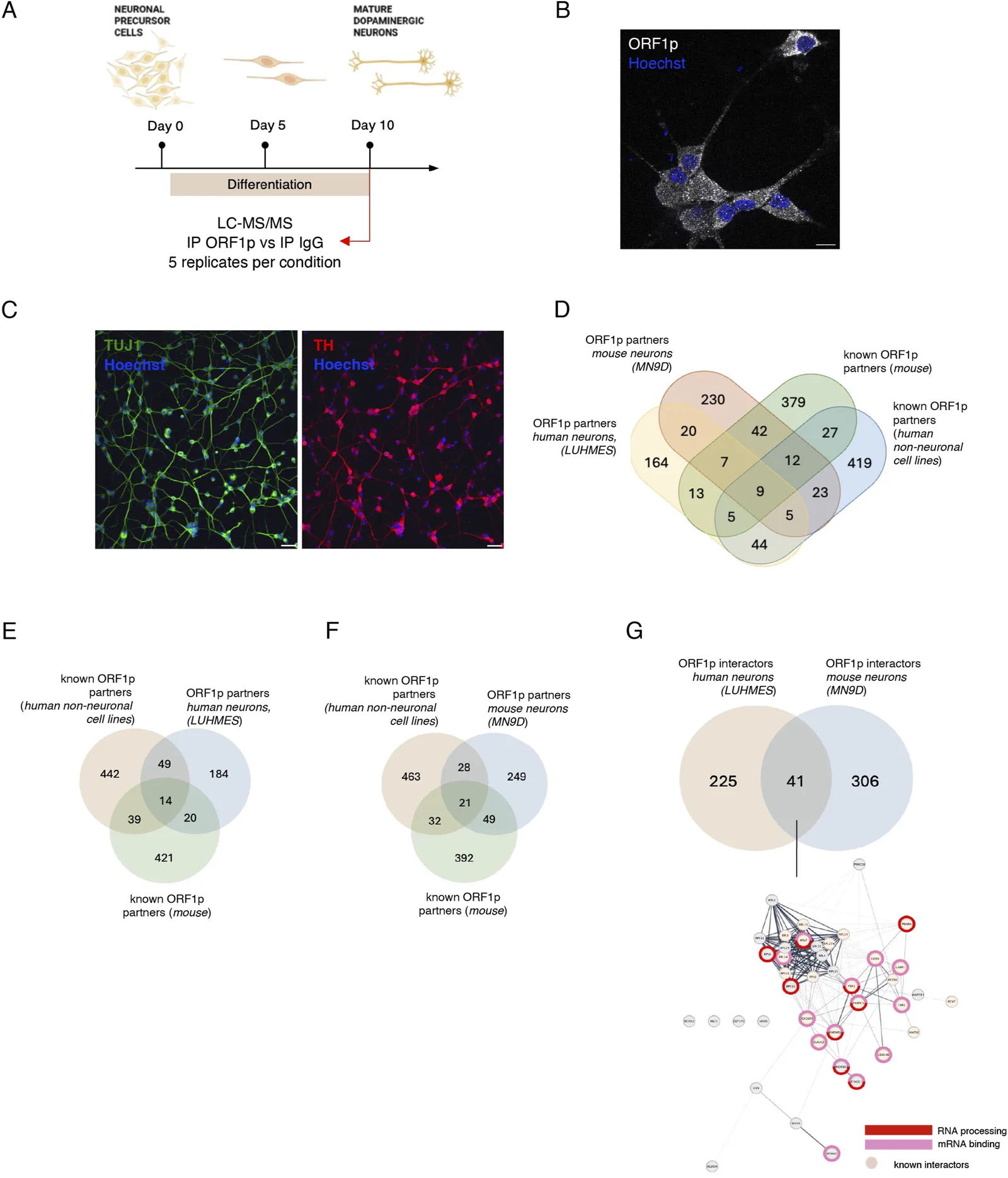
Differentiation process of MN9D cells and immunoprecipitation of ORF1p in MN9D and LUHMES cell lines. **(A)** Scheme of the differentiation protocol of MN9D cells into mature mouse dopaminergic neurons. All experiments (including quantitative mass spectrometry) were performed at day 10 of differentiation. **(B)** Immunofluorescence staining of endogenous ORF1p (ab216324) in differentiated MN9D neurons. **(C)** Immunofluorescence staining of MN9D at 10 d of differentiation expressing the neuronal marker TUJ1 (green, left) and the dopaminergic marker tyrosine hydroxylase (TH, red, right). Scale bar is 50 μm. **(D)** Venn diagram of proteins identified by quantitative mass spectrometry of immunoprecipitated endogenous ORF1p from mouse dopaminergic neurons (MN9D) and human dopaminergic neurons (LUHMES) compared with mouse and human published ORF1p interactors (19, 25, 26, 27, 28, 29, 30, 31, 32, 33, 34). **(E)** Venn diagram of proteins identified by quantitative mass spectrometry of immunoprecipitated endogenous ORF1p from human dopaminergic neurons (LUHMES) compared with previously published mouse (19, 32) and human (25, 26, 27, 28, 29, 30, 31, 33, 34) ORF1p. **(F)** Venn diagram of proteins identified by quantitative mass spectrometry of immunoprecipitated endogenous ORF1p from mouse dopaminergic neurons (MN9D) compared with previously published mouse (19, 32) and human (25, 26, 27, 28, 29, 30, 31, 33, 34) ORF1p interactors. **(G)** Common partners of ORF1p in LUHMES and MN9D neurons and their physical interactions identified by quantitative mass spectrometry.

**Figure 2.**
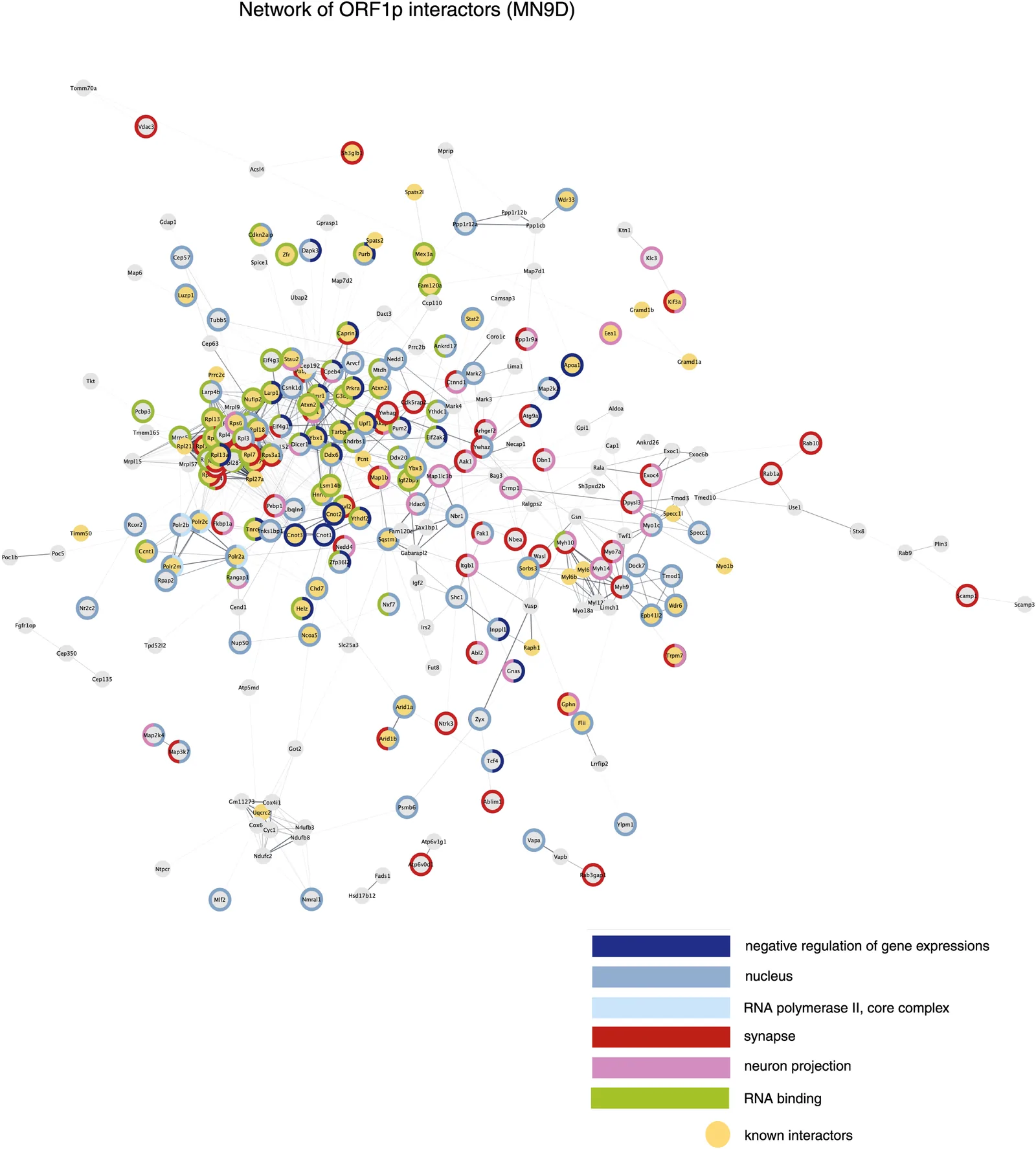
Physical protein–protein interactions (PPI) network of ORF1p protein partners identified by mass spectrometry in MN9D neurons. Physical PPI network of ORF1p protein partners identified by mass spectrometry in MN9D neurons using the STRING database and visualized with Cytoscape. ORF1p protein partners belong to different functional and enriched GO terms (rings): nucleus (dark blue), negative regulation of gene expression (blue), RNA polymerase II, core complex (light blue), synapse (red), neuron projection (pink), and RNA binding (green). Interactors previously identified are highlighted in yellow nodes. The thickness of the edges is proportional to the confidence in the physical interaction. Only proteins with physical interactions are shown. Details on proteins belonging to each GO category can be found in Table S2.

**Figure 3.**
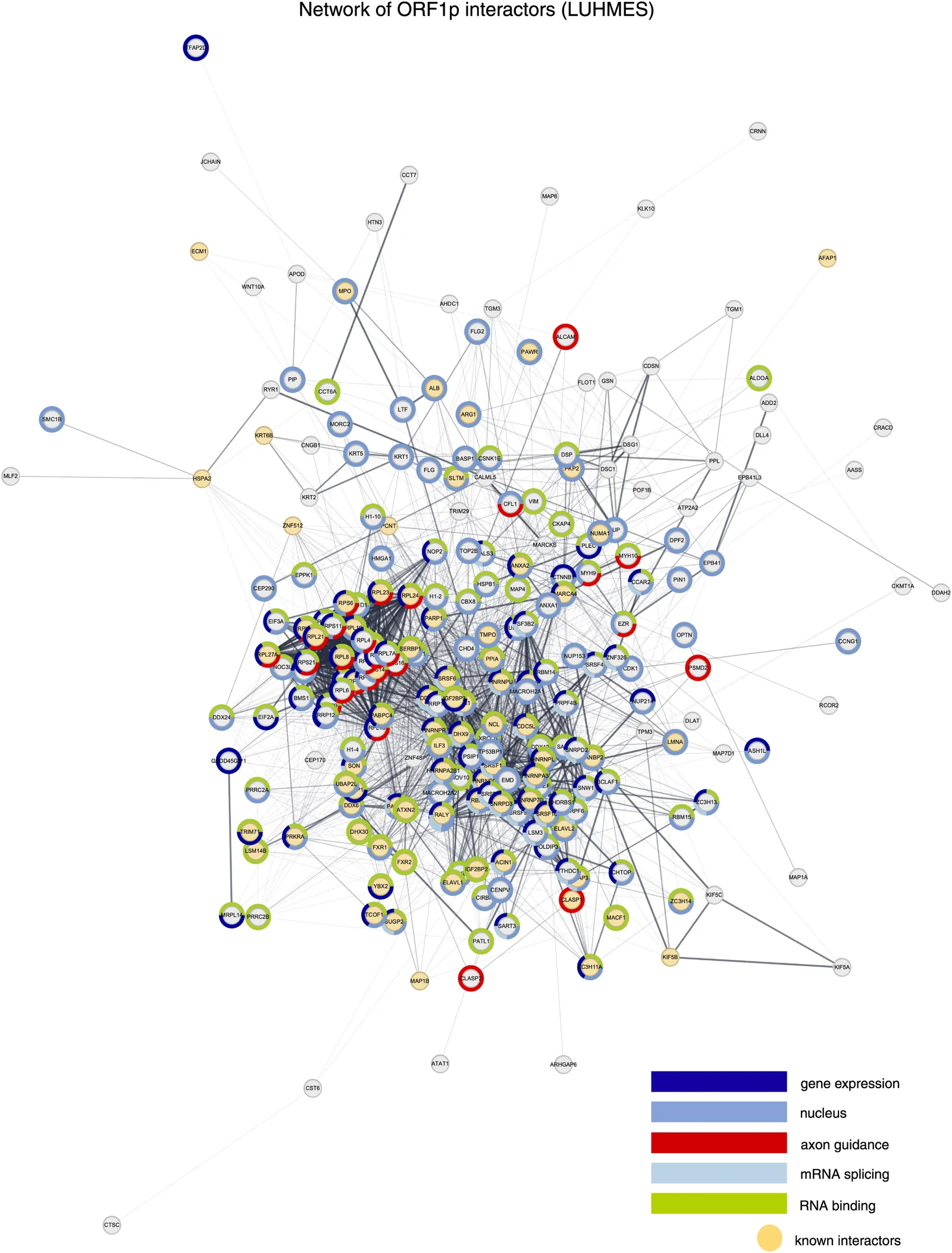
Physical protein–protein interactions (PPI) network of ORF1p protein partners identified by mass spectrometry in LUHMES neurons. Physical PPI network of ORF1p partners found in LUHMES neurons by mass spectrometry using the STRING database and visualized via Cytoscape. Partners belong to different functional enriched GO terms (rings): gene expression (dark blue), nucleus (blue), axon guidance (red), mRNA splicing (light blue), and RNA binding (green). Interactors previously identified are highlighted in yellow nodes. The thickness of the edges is proportional to the confidence in the physical interaction. Only proteins with physical interactions are shown. Details on proteins belonging to each GO category can be found in Table S2.

We and others have previously observed a widespread expression of ORF1p in mouse and human neurons at steady-state (19, 20, 21, 22). This somewhat surprising presence of ORF1p in neurons evokes the question of a possible physiological role of ORF1p specific to these cells. In a first attempt to address this question, we identified, using a quantitative mass spectrometry approach, protein partners of endogenous ORF1p in mouse (differentiated MN9D) and human (differentiated Lund human mesencephalic (LUHMES) (21, 23)) dopaminergic neurons (Fig 1A) using well-characterized and specific antibodies to ORF1p (19). Just as differentiated human dopaminergic LUHMES neurons (21, 23), mouse dopaminergic MND9 precursor cells can be differentiated into mature, post-mitotic neurons (Fig S1A) which express ORF1p (Fig S1B) along with the neuronal marker β-III tubulin (TUJ1, Fig S1C) and the dopaminergic marker tyrosine hydroxylase (TH, Fig S1C). As expected, the pattern of ORF1p staining was mainly cytoplasmic, but we also observed, as in human dopaminergic LUHMES (21), a readily detectable nuclear staining (Fig S1B). It is important to note that human and mouse ORF1p differ significantly in their amino acid composition (31% identity, NCBI Needleman-Wunsch Global Align) but share the same protein domains (24) which is reflected in very similar AlphaFold predicted 3D protein structures for mouse (https://alphafold.ebi.ac.uk/entry/AF-P11260-F1) and human (https://alphafold.ebi.ac.uk/entry/Q9UN81) ORF1p. After immunoprecipitation (IP) of endogenous ORF1p or isotype control IgG in human (LUHMES) and mouse (MN9D) dopaminergic neurons (n = 5 IPs per condition), we performed liquid chromatography coupled to mass spectrometry (LC-MS/MS) followed by a quantitative analysis (Fig 1A). ORF1p was strongly enriched in the immunoprecipitated material, with a high IP-ORF1p/IP-IgG ratio (mouse ORF1p: 3.76 log2(ratio), *P* = 4.16E-05, Fig 1B; human ORF1p: 5.35 log2(ratio), *P* = 8.73E-07, Fig 1C). 348 proteins were detected as ORF1p partners in MN9D (Fig 1B), and 267 proteins were identified as ORF1p partner proteins in LUHMES neurons (Fig 1C). As some proteins detected in the ORF1p IP were completely absent from the control IP-IgG and, as a consequence, no ratio could be calculated, these putative ORF1p protein partners are represented separately as peptides/100aa (Fig 1B and C, right panels). Next, we confronted ORF1p protein partners identified in neurons with those found in previous studies (25, 26, 27, 28, 29, 30, 31, 32, 33, 34), including our previous study of ORF1p interactors in the mouse brain (19). Nine proteins were identified in all studies (Fig S1D), namely, RPL19, IGF2BP3, LARP1, ATXN2, RPL24, YBX1, DDX6, PABPC1, and RPL8, representing, thus, high-confidence, highly conserved, and non-cell-type–specific ORF1p protein partners, most of which were RBPs, just as ORF1p itself (Fig S1H and I). 49 proteins were shared between ORF1p partners identified in various human cell types and ORF1p partners identified in human LUHMES neurons (Fig S1E), among them: HNRNPU, NCL, and IGF2BP2*,* and of those, 14 proteins were overlapping in addition to ORF1p protein partners identified in mouse MN9D neurons, including RPL19, IGF2BP3, RALY, LARP, ATXN2, RPL24, YBX1, CLASP1, DDX6, UBAP2L, DDX17, ELAVL1, PABPC1, and RPL8 (Table S1). Similarly, 70 proteins among which were GPHN, ELAVL2, ARID1B, FXR1, POLR2A, MAP1B, EEA1, ARID1A, and HELZ were in common between previously identified mouse ORF1p partners and those identified in MN9D neurons (Fig S1F). Focusing on overlapping ORF1p protein partners in mouse and human neurons (Fig S1G), the 41 proteins that were shared mostly identified as “RNA processing” and/or “RNA binding” in a STRING analysis visualized by Cytoscape (Fig S1G). To identify conserved categories of ORF1p protein partners between mouse and human neurons, we inquired for shared significant GO terms. As expected, among the top shared categories, many GO terms were related to RNA metabolism (Fig 1D, cat.III = category 3 interactors). However, two unexpected categories emerged: first, in both mouse and human neurons, ORF1p protein partners were enriched in GO terms related to nuclear presence and function (cat.I = category I interactors), namely “gene expression” (107 proteins in LUHMES [fold enrichment (FE): 3.25; FDR: 1.61E-26], 76 in MN9D [FE: 1.76; FDR: 0.0001], 23 in common) and “nucleus” (186 proteins in LUHMES [FE: 1.37; FDR: 3.07E-05], 162 in MN9D [FE: 1.82; FDR: 3.47E-22], 23 in common). In this category I of ORF1p protein partners, 31 unique proteins were identified, among which were 17 ribosomal proteins with non-canonical functions, in addition to DDX6, FXR1, GSN, IGF2BP3, KHDRBS1, LARP1, MLF2, MYH9, PABPC1, PRKRA, YBX1, HNRNPU, YTHDC1, and RCOR2, the last three of which have chromatin-related functions. In LUHMES cells, ORF1p interacted with proteins related to chromatin organization and epigenetic regulation (H1-2, H1-4, H1-10, MACROH2A1, MACROH2A2, CHD4, CBX8, ASH1L, SUPT16H, DPF2, SMARCA4, MORC2), proteins related to DNA damage and repair (TOP2B, PARP1, TP53BP1, XRCC6 (Ku70), and GADD45GP1), proteins involved in transcriptional regulation (CTNNB1, YBX1, ZC3H11A, THRAP3, SNW1, BCLAF1, PSIP1, TFAP2D), proteins associated with nuclear structure and envelope (LMNA, LBR, TMPO, EMD, NUP153, NUP214, RANBP2, NUMA1) and nuclear transport and signalling (PAWR, FXR1, and FXR2), in addition to many more involved in RNA processing, RNA metabolism, and ribosome biogenesis (Table S1). In MN9D neurons, ORF1p interacted with chromatin remodelers (CHD7, ARID1B, ARID1A); transcription factors (TFs) (ZFHX3, STAT2, ZNF638, ZNF281, TCF4); RNA polymerase subunits or associated proteins (POLR2M, POLR2A, POLR2B, POLR2C, RPAP2); and nuclear transport proteins (NUP50 and KPNA3) in addition to many RNA metabolism-related proteins (Table S1). In the second category (cat.II interactors), ORF1p protein partners were enriched for GO terms related to neuron morphology like “synapse” (LUHMES; FE: 1.92; FDR: 001; MN9D; FE: 3.62; FDR: 13E-32), “neuron projection” (LUHMES; FE: 1.85; FDR: 0.01; MN9D; FE: 2.46; FDR: 6.35E-10), “dendrite” (LUHMES; FE: 2.15; FDR: 0.0313; MN9D; FE: 3.13; FDR: 1.93E-09), “axo-dendritic transport” (LUHMES; FE: 5.84; FDR: 0.04; MN9D; FE: 5.53; FDR: 0.008), “cytoskeleton protein binding” (LUHMES; FE: 2.73; FDR: 1.12E-05; MN9D; FE: 4.04; FDR: 1.01E-19), and “actin binding” (LUHMES; FE: 3.91; FDR: 7.70E-06; MN9D; FE: 4.91; FDR: 3.10E-12) (Fig 1D). STRING analysis of physical protein–protein interactions (PPIs) of ORF1p partners in mouse (Fig 2) and human (Fig 3) neurons revealed the interaction profile of these proteins among each other according to their functional category (colours), confidence in physical interaction (thickness of edges), and known interactors (yellow nodes; please also see Table S2 for further information on proteins belonging to each category). The emergence of categories related to nuclear functions, including gene expression, was particularly intriguing in light of observations of a nuclear localization of ORF1p at steady-state in human neurons (21). Indeed, whereas ORF1p has been previously observed in the nucleus in different cell lines (28, 35, 36, 37, 38, 39, 40, 41) and in human post-mortem neurons (19, 20), the role of nuclear ORF1p has not been previously addressed except with regard to retrotransposition efficiency (42). We, therefore, asked the question whether nuclear ORF1p might have a role in mature neurons, potentially independent of the LINE-1 life cycle.

Table S1. ORF1p protein partners in LUHMES and MN9D neurons as identified by quantitative mass spectrometry.

Table S2. Details on proteins belonging to each GO category illustrated in Figs 2 and 3/Network of ORF1p interactors.

### ORF1p is located in the nucleus and associated to chromatin in post-mitotic neurons

**Figure 4.**
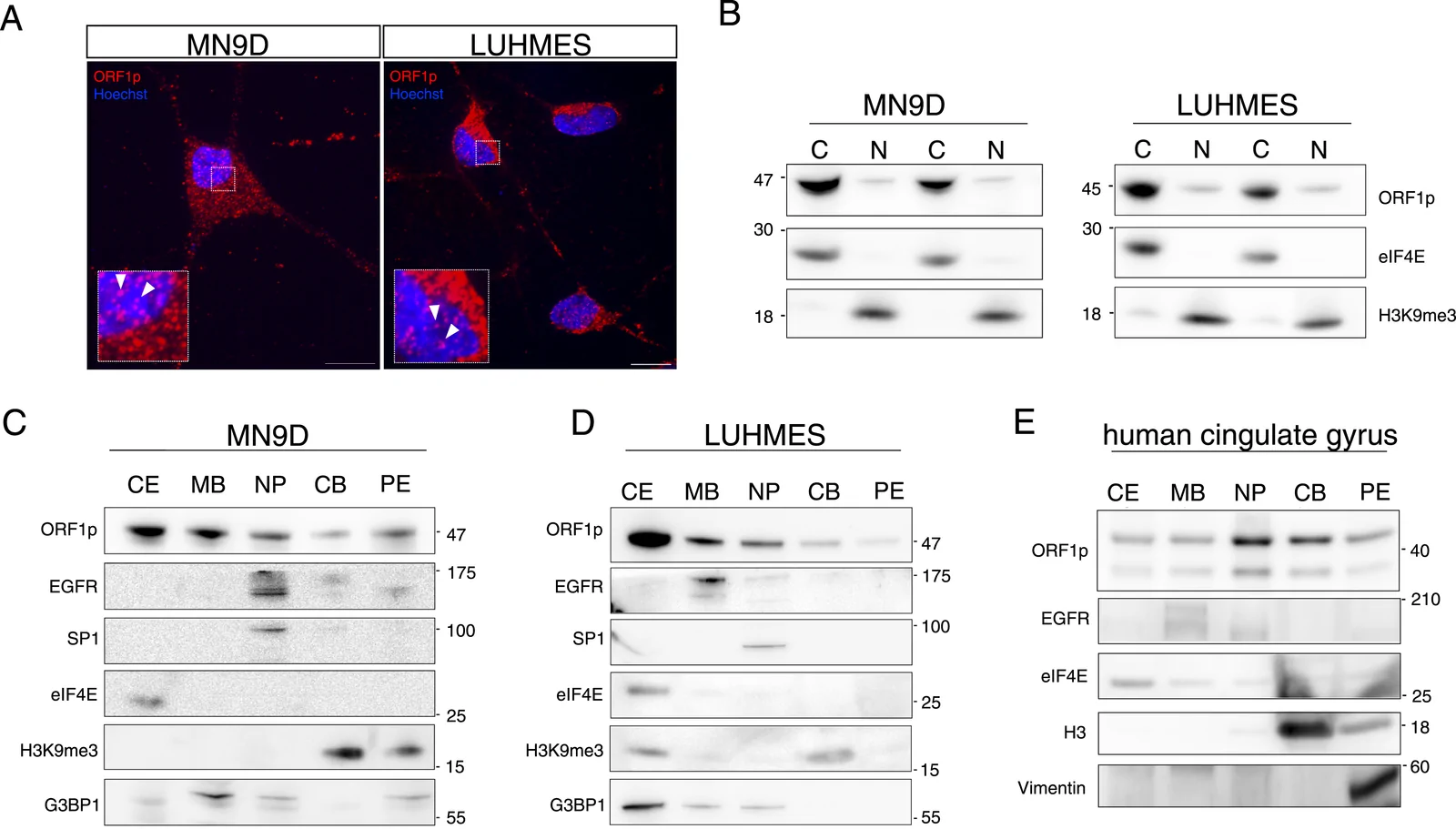
ORF1p is located in the nucleus and associated with chromatin in post-mitotic neurons. **(A)** Immunostainings of endogenous ORF1p in LUHMES and MN9D neurons. Nuclear localization of endogenous ORF1p is indicated by white arrows (antibody human ORF1p: Millipore MABC1152; antibody mouse ORF1p: Abcam ab216324). Scale bar is 10 μm. **(B)** Biochemical separation of the nuclear and cytoplasmic compartments of two independent samples followed by Western blot using an ORF1p antibody and two markers, one for the nuclear compartment (H3K9me3) and one for the cytoplasmic compartment (eIF4E). ORF1p is present in the cytoplasmic and the nuclear fraction of LUHMES and MN9D neurons. A representative experiment is shown (n = 3 independent experiments). *C*: *Cytoplasm, N: Nucleus*. **(C)** Subcellular fractionation of the cytoplasm (CE), the membrane-bound fraction (MB), the nucleoplasm (NP), the chromatin-bound fraction (CB), and the pellet (PE) of cell lysates of MN9D neurons, followed by Western blotting using an ORF1p antibody and different protein markers (CE marker: eiF4E; MB marker: EGFR; NP marker: SP1; CB marker: H3K9me3; stress granule marker: G3BP1). ORF1p is present in all compartments, including the chromatin-bound fraction. A representative experiment is shown (n = 3 independent experiments). **(D)** Subcellular fractionation of the cytoplasm (CE), the membrane-bound fraction (MB), the nucleoplasm (NP), the chromatin-bound fraction (CB), and the pellet (PE) of cell lysates of LUHMES neurons followed by Western blotting using an ORF1p antibody and different protein markers (CE marker: eiF4E; MB marker: EGFR; NP marker: SP1; CB marker: H3K9me3; stress granule marker: G3BP1). ORF1p is present in all compartments, including the chromatin-bound fraction. A representative experiment is shown (n = 3 independent experiments). **(E)** Subcellular fractionation of the cytoplasm (CE), the membrane-bound fraction (MB), the nucleoplasm (NP), the chromatin-bound fraction (CB), and the pellet (PE) of lysates of human post-mortem cingulate gyrus tissue of a neurologically healthy individual, followed by Western blotting using an ORF1p antibody and different protein markers (CE marker: eiF4E; MB marker: EGFR; NP marker: SP1; CB marker: H3. PE marker: Vimentin 1. ORF1p is present in all compartments, including the chromatin-bound fraction. A representative experiment is shown (n = 3 independent experiments).

Based on the identification of chromatin-related proteins as ORF1p protein partners, we characterized the subcellular localization of ORF1p in mouse and human neurons. Using immunofluorescence, we observed a predominant cytoplasmic expression of ORF1p in both LUHMES and MN9D neurons but also distinct nuclear dots in both species (Fig 4A). Cytoplasmic and nuclear fractionation confirmed the presence of ORF1p in the nuclear fraction of mouse and human post-mitotic neurons (Fig 4B). To gain a more precise understanding of the subcellular localization of ORF1p, we performed a more detailed biochemical fractionation, which confirmed that ORF1p is predominantly located in the cytoplasm but also present in the membrane-bound fraction (Fig 4C–E). Furthermore, ORF1p was localized to the soluble nuclear fraction (=nucleoplasm) and, importantly, reliably detected as chromatin-bound in the chromatin fraction of mouse neurons (Fig 4C), human neurons (Fig 4D), and human post-mortem tissue extracts from the cingulate gyrus of a neurologically healthy individual (Fig 4E). We confirmed the separation of all fractions using dedicated marker proteins and verified that stress granules were not contaminating the chromatin-bound fraction using the stress granule marker G3BP1 (Fig 4C and D). The presence of ORF1p on chromatin is in line with previous observations indicating the association of ORF1p with chromatin independently of ORF2p in cancer cells (43). Together, our observations suggest a close proximity and potentially functional relation of ORF1p with chromatin in neurons.

### Changes in nuclear ORF1p levels correlate with alterations in chromatin accessibility patterns in neurons

**Figure 5.**
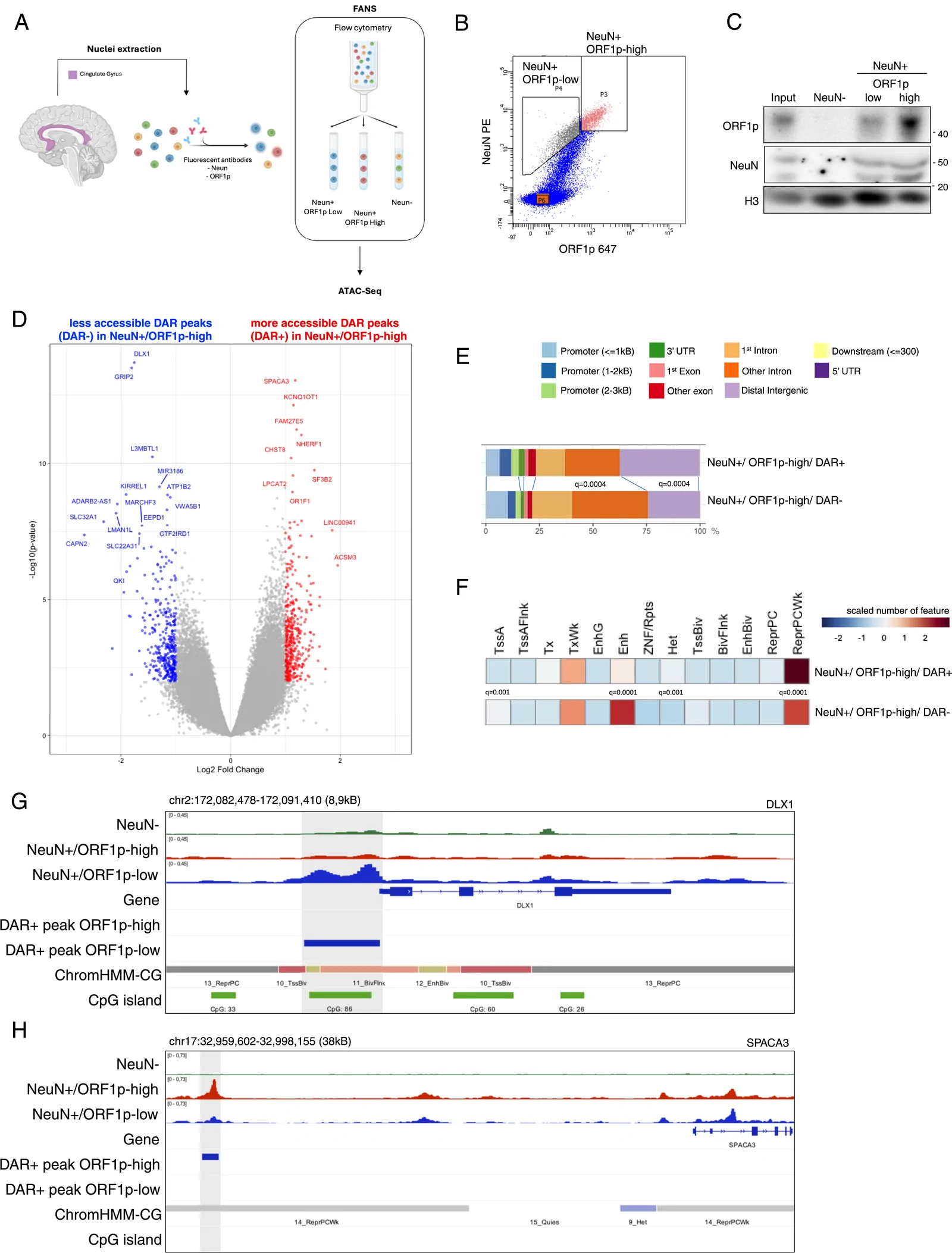
Nuclear ORF1p levels are associated with chromatin landscape variations in neurons. **(A)** Graphical representation of the experimental setup using fluorescent-activated nuclei sorting (FANS) to isolate neuronal nuclei with high or low nuclear ORF1p content from human post-mortem cingulate gyrus. Nuclei were extracted from pulverized frozen tissues of the post-mortem cingulate gyrus of neurologically healthy individuals (n = 3) and individuals diagnosed with Parkinson’s disease (n = 3). Nuclei were sorted with an antibody against NeuN to separate neurons (NeuN+) from non-neuronal cells (NeuN−) and an antibody against ORF1p to separate neuronal nuclei into two populations: those containing high amounts of ORF1p (ORF1p-high) and those containing no or low amounts of ORF1p (ORF1p-low). Sorted populations were then subjected to ATAC sequencing. **(B)** Representative fluorescent-activated nuclei sorting (FANS) profile showing the separation of non-neuronal cells (NeuN−; P6), NeuN+ cells (P3+P4) with either low (P4) or high (P3) nuclear ORF1p levels. **(C)** Western blot of nuclei sorted by FANS. ORF1p is not detectable in non-neuronal cells (NeuN−). Higher amounts of ORF1p are present in the sorted population of ORF1p-high neuronal nuclei compared with ORF1p-low or ORF1p-negative neuronal nuclei. NeuN was used as a control for the separation of neuronal from non-neuronal nuclei. H3 was used as a loading control. **(D)** Volcano plot of differentially accessible regions (DARs) identified by ATAC-seq comparing ORF1p-high and ORF1p-low human post-mortem neurons LUHMES. Red: significantly more accessible regions (DAR+) in NeuN+ ORF1p-high; Blue: significantly less accessible regions in NeuN+ ORF1p-high (DAR−) (log2 fold change ≤−1, *P*-value ≤0.01). **(E)** Genomic features associated with differentially accessible-region (DAR) in NeuN+/ORF1p-high and NeuN+/ORF1p-low. **(F)** Annotation of functional genomic features overlapping significant DARs using human post-mortem cingulate gyrus ChromHMM data retrieved from Roadmap. **(G)** IGV track of an example of DAR+ regions in ORF1p-low neuronal nuclei in the *DLX1/2* genomic locus. **(H)** IGV track of an example of a DAR+ region in ORF1p-high neuronal nuclei in the *SPACA3* genomic locus.

**Figure S2.**
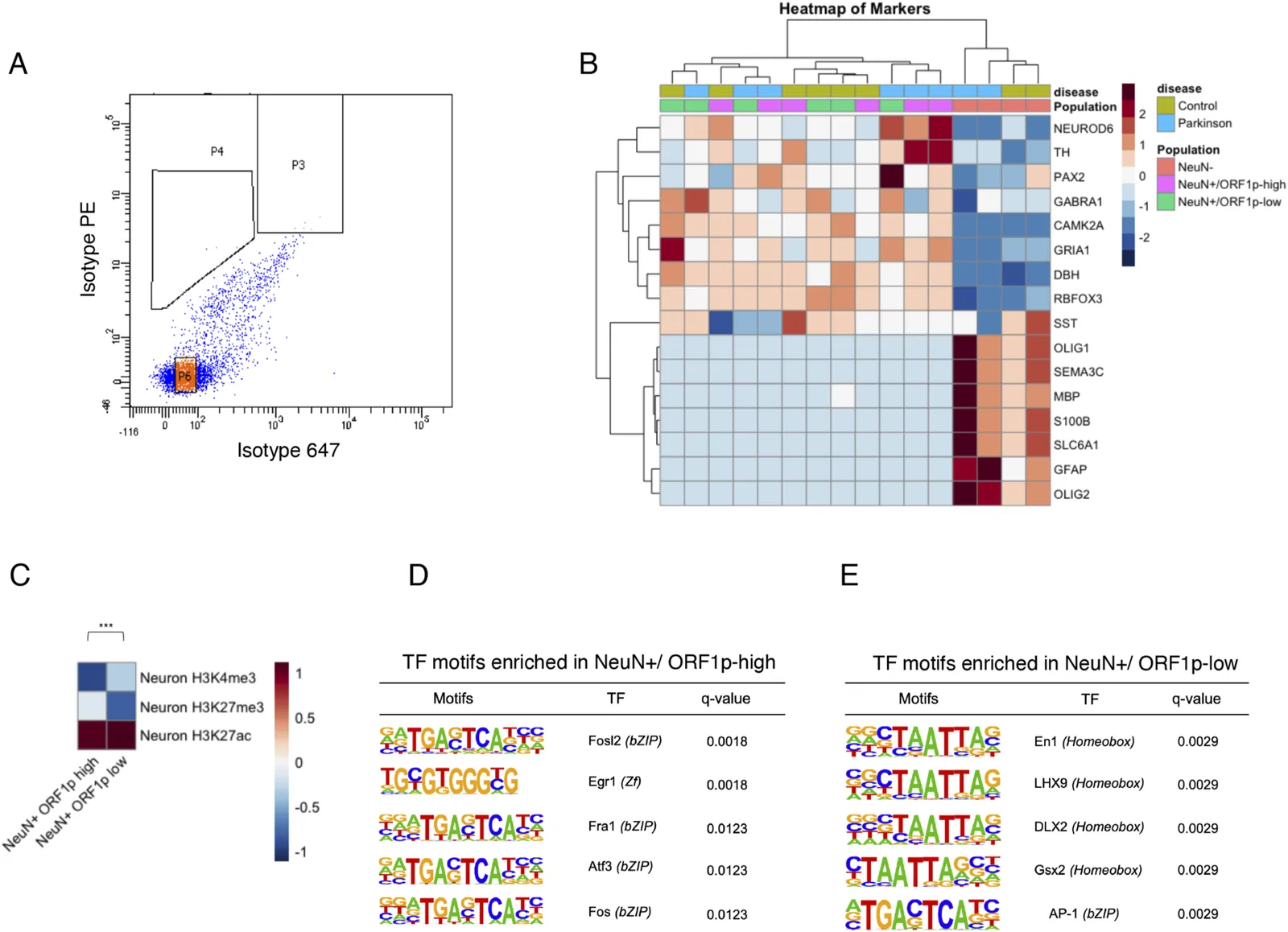
ATAC-seq analysis of FANS sorted human post-mortem nuclei. **(A)** Representative fluorescent-activated nuclei sorting (FANS) profile showing the isotype controls for the NeuN and ORF1p antibody used in Fig 5B. **(B)** Heatmap of brain cell type markers and ATAC-seq promoter peak numbers (<1 kB) in NeuN−, NeuN+ ORF1p-low, and NeuN+ ORF1p-high populations. NeuroD6, TH, PAX2, GABRA1, CAMK2A, GRIA1, DBH, RBFOX3 (=NeuN), and SST are markers of specific neuronal populations (https://biomarkerres.biomedcentral.com/articles/10.1186/s40364-023-00523-3) and show a high accessibility on promoters in both, ORF1p-high and ORF1p-low nuclei. OLIG1, SEMA3C, MBP, S100B, SLC6A1, GFAP and OLIG2 are glia-cell markers and show higher chromatin accessibility in promoter regions in non-neuronal (NeuN−) nuclei. **(C)** Heatmap of DARs in ORF1p-high and ORF1p-low nuclei and their overlap with the histone marks H3K4me3, H3K27me3 and H3K27ac. Histone post-translational modification profiles were retrieved from a previously published dataset (45). Two-sided Fisher's Exact test, followed by Benjamini, Krieger and Yekutieli post hoc test with Q:1%, ***P < 0.01. **(D, E)** Motif enrichment analysis of significant DARs in ORF1p-high and ORF1p-low neuronal populations.

To explore whether ORF1p might have a functional impact on chromatin organization, we turned to an assay for transposase-accessible chromatin followed by sequencing (ATAC-seq). As we had previously shown that ORF1p was present in neurons in the human cingulate gyrus (17) and established its presence in the chromatin-bound fraction in this brain region (Fig 4E), we isolated nuclei from frozen human post-mortem cingulate gyrus tissues of six individuals (n = 3 neurologically healthy individuals and n = 3 individuals with Parkinson disease, Table S3). We then sorted them as schematized in Fig 5A using two antibodies, the neuronal marker NeuN, separating neurons from non-neuronal nuclei, and ORF1p, separating nuclei with high levels of ORF1p (ORF1p-high; P3, Fig 5B) and those with lower levels of ORF1p, including those not expressing ORF1p (ORF1p-low; P4, Fig 5B). The corresponding isotype control profiles are shown in Fig S2A. Confirming our previous observations of a predominant neuronal expression of ORF1p in the mouse and human brain (19), non-neuronal nuclei were ORF1p negative, whereas neurons contained varying levels of nuclear ORF1p, allowing us to separate three distinct populations, NeuN−/ORF1p-minus; NeuN+/ORF1p-high; and NeuN+/ORF1p-low, which we also verified by Western blot (Fig 5C). This confirmed the absence of ORF1p in non-neuronal nuclei and the successful sorting of two neuronal populations with either high or low levels of nuclear ORF1p. We then performed high-throughput chromatin profiling on those three populations. To exclude that ORF1p-high and ORF1p-low nuclei were assimilated to a specific neuronal subtype, we plotted total ATAC-seq reads on promoter regions (<1 kb) for cell type–specific markers selected from Single Cell Base (https://biomarkerres.biomedcentral.com/articles/10.1186/s40364-023-00523-3) which showed a clear separation of NeuN− and NeuN+ accessibility profiles as expected and, importantly, no overt difference between accessibility profiles on neuronal subpopulation marker genes between ORF1p-high and -low nuclei (Fig S2B). Based on the number of normalized peaks, we determined DARs. The volcano plot in Fig 5D depicts all significantly enriched DARs, with 406 DARs more accessible in the NeuN+/ORF1p-high population (DAR+), and 379 DARs less accessible in NeuN+/ORF1p-high nuclei (DAR− ≈ more accessible in NeuN+/ORF1-low neurons; log2 fold-change ≥1 / ≤−1, *P*-value ≤0.01). We next analysed genomic features associated with each DAR. DARs were mostly located in introns (first and others) and in distal intergenic and, to a lesser extent, in promoter regions (Fig 5E). However, there were significant differences in the distribution of DARs over genomic features between NeuN+/ORF1p-high and NeuN+/ORF1p-low nuclei. NeuN+/ORF1p-high/DAR− regions were enriched in introns (q = 0.0004; Fisher’s Exact test followed by Benjamini, Krieger, and Yekutieli post-hoc test) compared with NeuN+/ORF1p-high/DAR+ regions, which were more frequently located in distal intergenic regions (q = 0.0004; Fisher’s Exact test followed by Benjamini, Krieger, and Yekutieli post-hoc test). To gain a more precise idea about the functional features that DARs in both conditions were associated with, we overlapped DARs with regulatory features stemming from a ChromHHM annotation, which defines and characterizes chromatin states across the genome based on experimental data of a given tissue, in this case, of human *post-mortem* cingulate gyrus (Fig 5F). Less accessible chromatin (DAR−) in ORF1p-high nuclei coincided with enhancers (Enh), weak repressed polycomb (ReprPCWk) domains, and domains associated with weak transcription (TxWk), whereas more accessible chromatin (DAR+) in ORF1p-high nuclei was mostly located in weak repressed polycomb (ReprPCWk) and weak transcription (TxWk) domains (Fig 5F). The distribution of DARs between ORF1p-high and ORF1p-low nuclei was significantly different, with an overrepresentation of enhancers (Enh, q = 0.0001) and active transcriptional start sites (TssA, q = 0.0013) among DAR− regions and an overrepresentation of repressed polycomb regions (ReprPCWk, q = 0.0001) and heterochromatin domains (Het, q = 0.001) in DAR+ regions in ORF1p-high nuclei (Fisher’s Exact test followed by Benjamini, Krieger, and Yekutieli post-hoc test). This indicated the existence of two different profiles of chromatin accessibility between these two nuclei populations. ORF1p-low nuclei were characterized by more accessible chromatin in intronic regions related to active transcription and enhancer activity, and ORF1p-high nuclei showed open chromatin in repressed or heterochromatin regions rather than located in distal intergenic genomic regions. To test whether DARs were present on young LINE-1 elements, we overlapped the genomic intervals of all DARs and L1HS and L1PA2 elements and found one single overlap (chr3:195087505-195087829, intronic L1PA2 within the *XXYLT1* gene), suggesting that LINE1 RNA and/or encoding proteins do not interfere with the regulation of the chromatin accessibility of young LINE-1 sequences. We next visualized the top two candidates of each category using IGV. Tracks of *DLX1* (distal-less homeobox 1) show an example of DAR+ regions in ORF1p-low neurons (Fig 5G). *DLX1* is a member of the homeobox TF family, predicted to be a transcriptional regulator for TGF-beta signalling and linked to autism (44). The DAR in the *DLX1* gene is annotated as a “flanking bivalent transcriptional start site/enhancer” and “bivalent enhancer” in adult cingulate gyrus ChromHMM annotations and overlaps with a CpG island. There are two other DARs in this region, one intergenic in-between *DLX1* and *DLX2* and one in the promoter region of *DLX2*, both characterized by bivalent enhancer or bivalent TSS annotations. These loci thus provide examples of the overrepresentation of DARs in ORF1p-low chromatin in intronic and promoter regions and transcriptional start sites and enhancers as depicted and quantified in Fig 5F. IGV tracks of *SPACA3* (Sperm Acrosome Associated 3) show an example of a DAR+ region in ORF1p-high neurons (Fig 5H). *SPACA3* is a sperm-specific gene, and the DAR+ peak in ORF1p-high chromatin overlaps with a “weak repressed polycomb” annotation and reflects the overrepresentation of ORF1p-high DAR+ regions in repressive chromatin regions (Fig 5F). Using ChIP-seq data from sorted human post-mortem neurons (45) for trimethylated Histone H3 at lysine 4 (H3K4me3), which marks transcriptional start sites of active genes; trimethylated Histone H3 at lysine 27 (H3K27me3), a histone modification associated with polycomb-mediated heterochromatin regions; and acetylated Histone H3 at lysine 27 (H3K27ac), a marker for active enhancers, we observed significant differences in proportions of DAR+ regions overlapping with all three histone profiles. H3K27me3 was overrepresented in ORF1p-high nuclei (q = 0.0003) in line with the repressive polycomb signature observed in the ChromHMM data, whereas H3K4me3 (q = 0.0007) and H3K27ac (q = 0.0003) were significantly overrepresented over DAR+ regions in ORF1p-low nuclei (Fig S2C, Fisher’s Exact test followed by Benjamini, Krieger, and Yekutieli post-hoc test), in line with the active transcription and enhancer profile observed in the ChromHMM data (Fig 5F). Together, these data indicated a distinct genomic and functional genomic profile of DARs dependent on ORF1p nuclear content, suggesting a regulatory function of nuclear ORF1p on chromatin dynamics. We then asked which TF-binding sites were overrepresented among the identified DARs and performed TF motif enrichment analysis with HOMER (Fig S2D and E). In DAR+ regions in neuronal nuclei with a high ORF1p content, predicted TF motifs were predominantly showing members of the highly conserved basic leucine zipper (bZIP) TF family, like ATF3, involved in axonal regeneration (46), and three members of the FOS TF family (FOSL2, FRA1, and FOS), which are TFs orchestrating the conversion of short-term extracellular signals into long-term functional changes via the activation of dedicated gene expression programs as members of the immediate early genes, including roles in memory formation (47). We also identified signatures of another immediate early gene, the zinc finger TF EGR1, within DAR+ regions in NeuN+/ORF1p-high nuclei. Interestingly, EGR1 is also involved in memory formation (47). TF signatures on ORF1p-low DAR+ regions were mainly composed of homeobox genes like Engrailed-1 (En1) (Fig S2E) which have important functions in brain patterning (48) but also in the maintenance of adult neurons (48).

Table S3. Metadata of post-mortem cingulate gyrus tissues used in FANS/ATAC-seq.

### LINE-1 knockdown induces changes in chromatin organization

**Figure 6.**
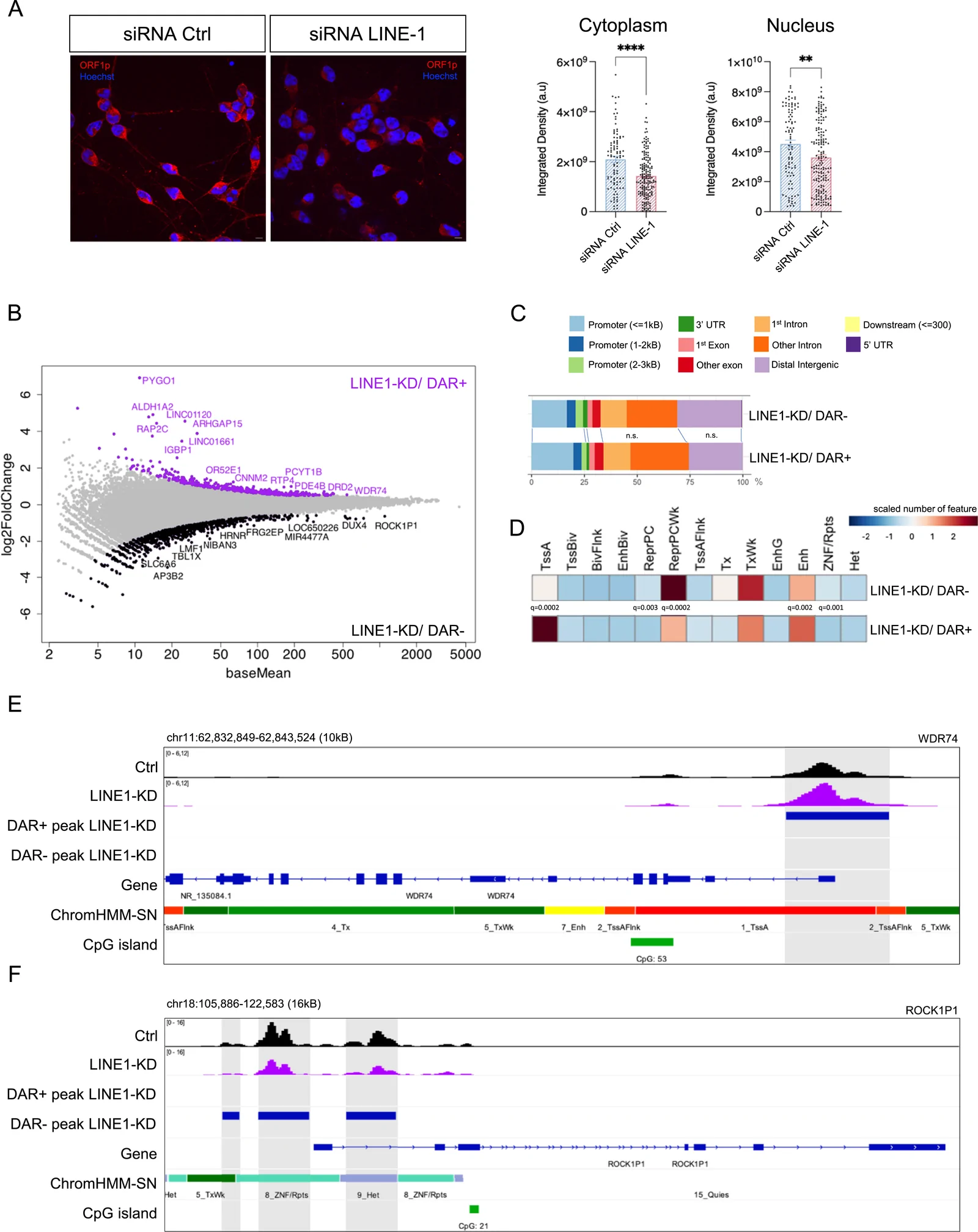
ORF1p knockdown induces changes in chromatin organization. **(A)** Representative image of ORF1p knockdown by immunofluorescence (left) and quantification of ORF1p staining intensity (right; each dot on the dot blot represents one image). Two-tailed Mann–Whitney *U test*. Error bars are represented as mean ± SEM. **P ≤ 0.01; ****P ≤ 0.0001. **(B)** MA-plot of differentially accessible regions (DAR) identified by ATAC-seq after LINE1-KD in human neurons (log2 fold change ≤−0.5, *P*-value ≤0.05). **(C)** Genomic features associated with DARs more (DAR+) or less accessible (DAR−) in LINE1-KD neurons. **(D)** Annotation of functional genomic features overlapping significant DARs using human post-mortem *substantia nigra* ChromHMM data retrieved from Roadmap. **(E)** IGV track of a DAR+ region in LINE1-KD neuronal nuclei as identified by ATAC-seq in the *WDR74* genomic locus. **(F)** IGV track of DAR− regions in neurons after LINE1-KD in the *ROCK1P1* gene locus.

**Figure S3.**
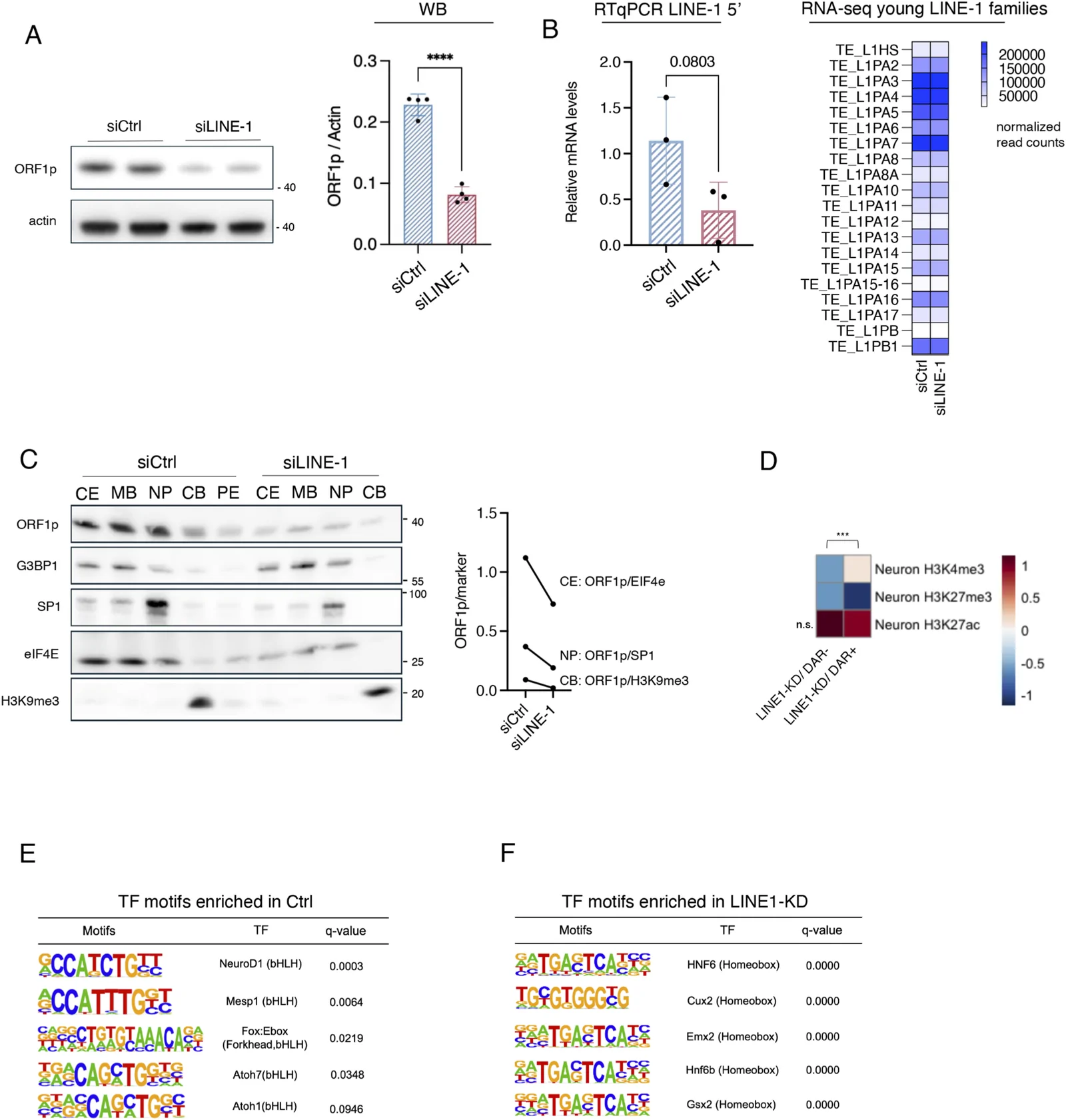
KD of LINE-1 in differentiated LUHMES cells followed by ATAC-seq analysis. **(A)** Western blot and quantification showing LINE1-KD in differentiated LUHMES neurons (n = 4 wells; three independent experiments, day 7 post-differentiation). Two-tailed Mann–Whitney *U test*. Data are represented as mean ± SEM. Mann–Whitney test, ****P ≤ 0.0001, n = 4. **(B)** LINE1-LOF on the RNA_level is not significant. LINE-1 5′UTR RTqPCR of control (siCtrl) and LINE1-KD (siLINE-1) in LUHMES neurons (left). Heatmap of RNA-seq data for young LINE-1 families, including the two families containing full-length and coding LINE-1 elements (L1HS and L1PA2) as well as older elements (L1PA3-17, L1PB, L1PB1) comparing control siRNA with siRNA against LINE-1 (mean of n = 4 in each condition, right). **(C)** Subcellular fractionation of control (siCtrl) or LINE1-KD (siLINE-1) LUHMES neurons. Shown are the cytoplasm (CE; marker: eIF4E), the membrane-bound fraction (MB), the nucleoplasm (NP; marker: SP1), the chromatin-bound fraction (CB; marker: H3K9me3), and the pellet (PE). **(D)** Heatmap of DARs in Ctrl and LINE1-KD nuclei and their overlap with the histone marks H3K4me3, H3K27me3, and H3K27ac. Histone post-translational modification profiles were retrieved from a previously published dataset (45). Two-sided Fisher's Exact test, followed by Benjamini, Krieger and Yekutieli post-hoc test with Q:1%, ***P < 0.01. **(E, F)** Motif analysis of significant DARs identified in ATAC-seq in control and LINE1-KD human dopaminergic neurons (LUHMES).

Having established significant changes in chromatin accessibility in nuclei differing in the levels of ORF1p content in the human cingulate gyrus, we then turned to human LUHMES neurons in which we experimentally manipulated endogenous ORF1p steady-state expression levels using LINE-1 siRNAs targeting evolutionary young LINE-1 elements. LUHMES neurons were transfected with siRNA at day three and retrieved at day seven of differentiation. Efficient knockdown of ORF1p was verified by immunostaining with a significant reduction in the cytoplasmic and nuclear compartments (Fig 6A) and by Western blot (Fig S3A), whereas LINE-1 RNA was slightly but not significantly reduced in independent RTqPCR experiments (Fig S3B, left) and not reduced at the family level of the youngest LINE-1 families in the RNA-seq data as depicted in the heatmap in Fig S3B, right).

We also verified by Western blot the loss-of-function of ORF1p after LINE-1 knockdown (LINE1-KD) in the chromatin-bound fraction (Fig S3C). Using this knockdown protocol, we performed ATAC-seq on LINE-1 siRNA-targeting ORF1 and control siRNA (Ctrl)-transfected neurons. The MA-plot in Fig 6B shows the DARs (log2 fold-change ≥1 / ≤−1, *P*-value ≤0.05), with 432 regions significantly more accessible (DAR+) after LINE1-KD and 917 less accessible regions (DAR−) compared with control. Although effect sizes were rather low, we observed many similarities to human post-mortem nuclei depending on the nuclear ORF1p content. Indeed, in nuclei with low ORF1p levels, DAR+ regions in the LINE1-KD condition were mostly located in introns and DAR− regions in distal intergenic regions, but with no significant overrepresentation when comparing the two conditions (Fig 6C). When analysing the regulatory regions overlapping DAR+/DAR− regions in LINE1-KD nuclei (Fig 6D) using a ChromHMM collection from human *substantia nigra* as a suitable reference for differentiated LUHMES cells which are dopaminergic (49), we identified a very similar profile as in ORF1p-high and ORF1p-low nuclei in the cingulate gyrus (Fig 5E). Indeed, just as in ORF1p-low nuclei, DAR+ regions in LINE1-KD nuclei were enriched on active transcriptional start sites (q = 0.0001) and enhancers (q = 0.03, Fisher’s exact test followed by Benjamini, Krieger, and Yekutieli post-hoc test). And similar to ORF1p-high nuclei, Ctrl DAR+ regions were overrepresented on repressed chromatin domains associated with polycomb (ReprPCWk, q = 0.0001; ReprPC, q = 0.005) and zinc finger genes/repeat-associated genomic regions (ZNF/Rpts, q = 0.001; Fisher’s Exact test followed by Benjamini, Krieger, and Yekutieli post-hoc test, Fig 6D). Again, using histone ChIP-seq data from sorted post-mortem neurons pooled from 5 different brain regions (45), we also found a significant overrepresentation in the fraction of DAR+ regions in LINE1-KD nuclei over H3K4me3 domains (q = 0.0001), whereas DAR− regions were overrepresented in H3K27me3 (q = 0.0001) domains (Fig S3D). We then chose representative examples for DAR+ regions in Ctrl (Fig 6E) and LINE1-KD (Fig 6F) for visualization. *WDR74* is an example of a DAR+ region in LINE1-KD neurons and is located in the promoter region overlapping with a TssA in line with the preferential location of DARs in LINE1-KD promoters depicted in Fig 6C and the overrepresentation of TssA regulatory regions more accessible in LINE1-KD (Fig 6D). *ROCK1P1*, a pseudogene, contains two DARs overlapping with “ZNF/Rpts,” “Het,” and “TxWk” annotations in line with regions overrepresented in LINE1-KD DAR− regions (Fig 6D). To test whether DARs were present on young LINE-1 elements, we overlapped the genomic intervals of all DARs and L1HS and L1PA2 elements and found no overlap, again suggesting that LINE-1 RNA and/or encoding proteins do not interfere with the regulation of the chromatin accessibility of young LINE-1 sequences. Next, we investigated the TF-binding sites enriched in those DAR. In DAR+ regions in Ctrl neurons, the family of basic helix–loop–helix (bHLH) TFs (Fig S3E) was strongly represented. These TFs have essential regulatory functions for neural cell fate specification and in embryonic development (50, 51). Interestingly, analogous to the TF profile overrepresented on DAR+ regions in ORF1p-low nuclei (Fig S2F), DAR+ regions in LINE1-KD nuclei were enriched in homeobox genes (Fig S3F). Therefore, low ORF1p nuclear content might favour accessibility to genomic regions regulated by homeobox proteins, which are essential for specifying cell identities and positioning during embryonic development but also play a role in the adult brain related to neuronal maintenance (52). Taken together, when comparing features across these two independent ATAC-seq experiments in human neurons (post-mortem cingulate gyrus neurons and differentiated dopaminergic neurons in culture) with high or low nuclear ORF1p content, we made similar observations: nuclei containing high levels of ORF1p showed more accessible chromatin preferentially in distal intergenic regions associated with polycomb-associated repressive domains, and nuclei with low ORF1p content were predominantly characterized by more accessible DARs in intronic regions associated with enhancer functions and active transcription containing signatures of homeobox TF-binding sites (Table 1). This might suggest that LINE-1 regulates cell identity in post-mitotic neurons.

**Table 1.** Significant characteristics of significantly enriched “differentially accessible regions” more accessible (DAR+) in neurons with either high or low nuclear ORF1p content identified by ATAC-seq of human post-mortem cingulate gyrus neurons and ATAC-seq of human dopaminergic neurons in culture (LUHMES).

​	ORF1p-high	ORF1p-low
GC		
Genomic region	**Distal intergenic**	Intronic
Functional feature	Heterochromatin	**Flanking active TSS**
	**Weak repressed polycomb**	**Enhancer**
Histone profile	**H3K27me3**	**H3K4me3**, H3K27ac
TF-binding sites	bZIP, ZF	**Homeobox**, bZIP

​	Control	ORF1p-LOF
LUHMES		
Genomic region	**Distal intergenic**	​
Functional feature	Repressed polycomb	**Flanking active TSS**
	**Weak repressed polycomb**	**Enhancer**
	ZNF/repeats	​
Histone profile	**H3K27me3**	**H3K4me3**
TF-binding sites	bHLH	**Homeobox**

Overlapping characteristics in both experimental systems are highlighted in bold.

### Nuclear ORF1p modulates chromatin accessibility on genes related to neuron-specific processes

**Figure 7.**
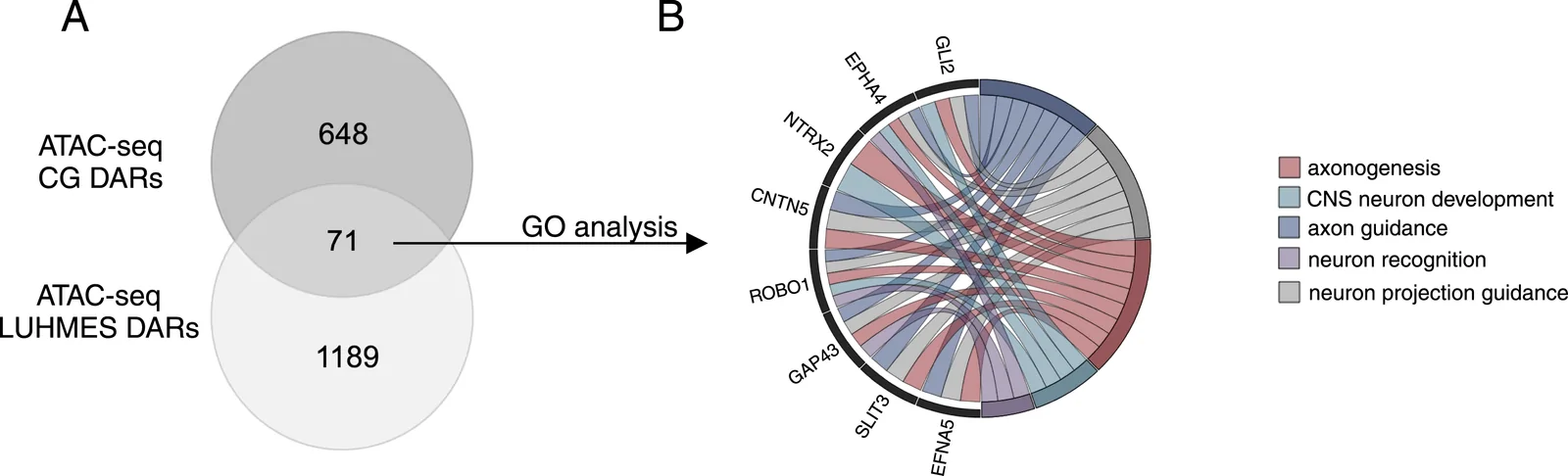
Nuclear ORF1p modulates chromatin accessibility on genes related to neuron-specific processes. **(A)** Venn diagram showing the overlap between ATAC-seq DARs from human neuronal nuclei containing high or low amounts of ORF1p (1260 DARs) and ATAC-seq DARs from control human dopaminergic neurons (LUHMES) compared with neurons after LINE1-KD (719 DARs). 71 genes are associated with DARs in both conditions. **(B)** GO analysis of 71 genes which are associated with at least one DAR in both ATAC-seq analyses. GO biological process complete; Fisher’s exact test with FDR correction. 52 of 71 were unambiguously mapped. Axon guidance (fold enrichment 11.92, FDR: 0.02, 7 genes).

We next compared the actual overlap of DAR-associated genes between both experimental systems. Of the 648 DAR-associated genes identified in cingulate gyrus neurons and the 1,189 DAR-associated genes identified in LUHMES neurons, 71 events were found to be shared between the two (Fig 7A). This overlap represents 10.96% of the CG DARs and 5.97% of the LUHMES DARs. Whereas this is only a modest overlap (Jaccard similarity coefficient 4.02%), GO analysis of these genes revealed five significant biological processes: axonogenesis, CNS neuron development, axon guidance, neuron recognition, and neuron projection guidance (Fig 7B). The GOChord plot in Fig 7B shows the eight genes sharing these GO terms. These findings reinforce the possibility of a role of nuclear ORF1p in neuron-specific functions.

### Knockdown of ORF1p affects the neuronal transcriptome, including down-regulation of long genes related to neuron-specific functions

**Figure 8.**
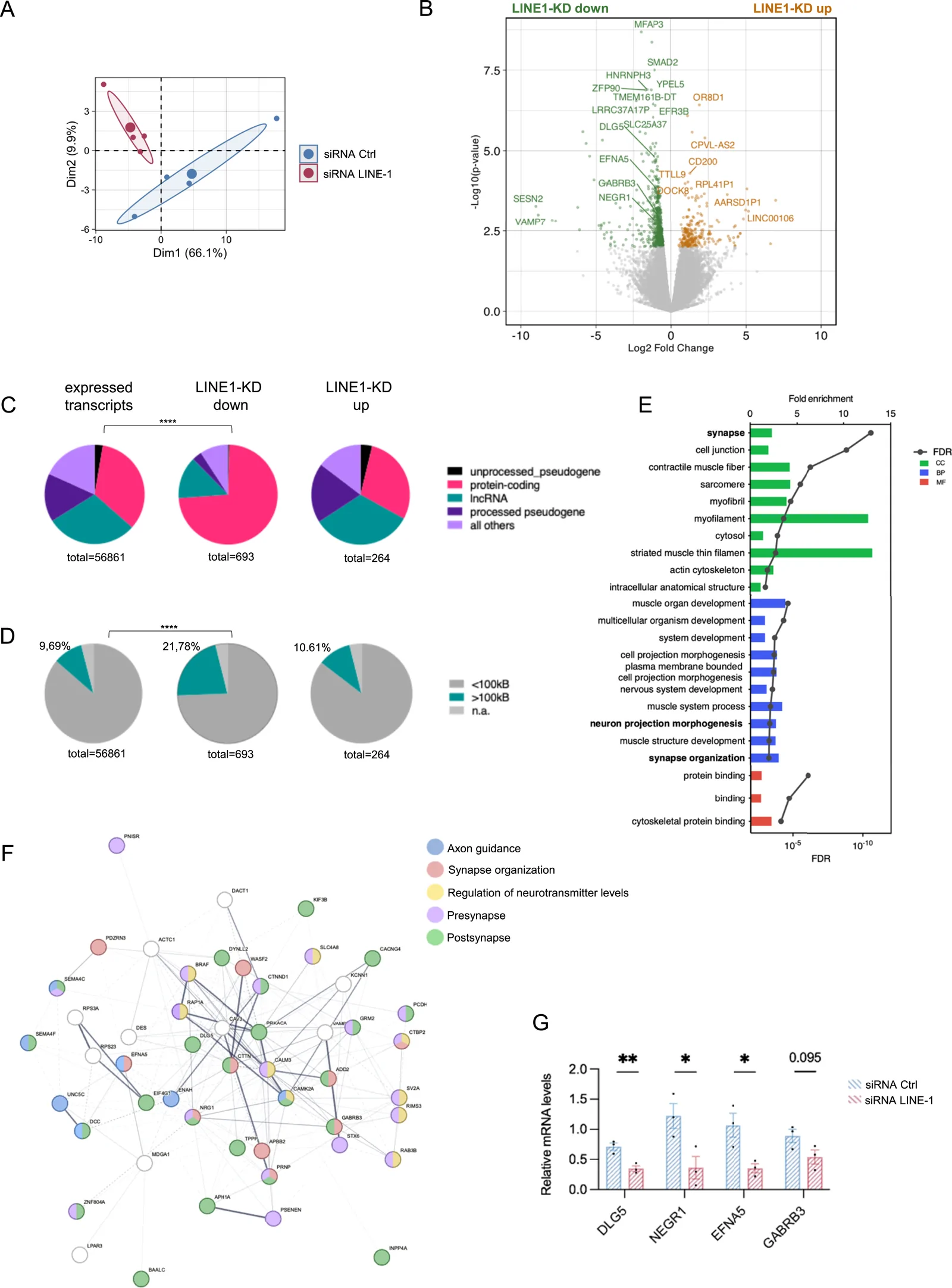
Loss of ORF1p leads to transcriptomic changes in neuron-specific genes. **(A)** Principal component analysis of RNA-seq data showing the separation of control neurons (blue) and ORF1p-KD neurons (red; n = 4 samples per condition, LUHMES). **(B)** Volcano plot showing differentially expressed genes (DEGs) in LUHMES neurons upon LINE1-KD. 698 genes were significantly up-regulated (in red: log2 fold change ≥0.5, *P*-value ≤ 0.01); 264 genes were significantly down-regulated (in blue: log2 fold change ≤−0.5, *P*-value ≤0.05). **(C)** Parts of whole graph showing the distribution of gene biotypes extracted from NCBI RefSeq among all expressed transcripts present in the RNA-seq analysis (left) or up-regulated (middle) or down-regulated transcripts (right) upon LINE1-KD. Distributions of gene biotypes among expressed or up-regulated transcripts were similar, while down-regulated transcripts were mainly protein-coding transcripts. Gene biotype overrepresentation analysis was performed using a Chi-square test with observed = number of feature counts in LINE1-KD and expected = all RNA-seq transcript counts. **(D)** Gene length analysis of all expressed transcripts in human LUHMES neurons (left), up-regulated (middle) or down-regulated transcripts (right) upon LINE1-KD. Genes were separated into two categories: long genes (>100 kB) and short genes (</=100 kB). The fraction of long genes among all and up-regulated transcripts was similar (around 10%) but was significantly higher among down-regulated transcripts (22%). Long gene overrepresentation analysis was performed using a Chi-square test with observed = number of feature counts in LINE1-KD and expected = all RNA-seq transcript counts. **(E)** GO analysis of significant down-regulated genes from LINE1-KD. FDR was calculated from *P*-values (Fisher’s exact test) by the Benjamini–Hochberg procedure. CC, cellular component; BP, biological process; MF, molecular function. **(F)** Protein–protein interaction (PPI) network of significantly down-regulated genes upon LINE1-KD (log2 fold change ≤−0.5, *P*-value ≤0.005) illustrated for the GO Cellular Component term “Synapse” GO:0045202 (50 genes). Genes are coloured according to their associated GO term. **(G)** Quantitative RT–PCR depicting the relative mRNA expression of *DLG1*, *NEGR1*, *EFNA5*, and *GABRB3* normalized to ENO2, TBP, and HPRT in LUHMES neurons treated with siRNA-Ctrl or siRNA-ORF1 (n = 3 per condition). Two-tailed Student’s *t* test. Data are represented as mean ± SEM *P ≤ 0.05; **P ≤ 0.01; ****P ≤ 0.0001.

A way to interrogate changes in neuronal cell identity and function is via the characterization of the transcriptome. Therefore, we performed RNA-seq analysis after LINE1-KD in differentiated LUHMES neurons. Principal component analysis separated Ctrl and LINE1-KD transcriptomes (Fig 8A). Using DESeq2, we identified 962 differentially expressed genes (DEG) (log2FC  ≥ 0.5 / ≤−0.5, *P*-value ≤0.01), of which 264 were up-regulated and the majority, 698 genes, were significantly down-regulated as shown in the volcano plot in Fig 8B. Characterization of major “gene biotypes” using Ensembl annotations (hg38) revealed a similar pattern between all LUHMES expressed transcripts (total = 56,861) and transcripts up-regulated upon LINE1-KD (total = 264) with almost equal proportions of protein-coding transcripts (34% Ctrl; 29% LINE1-KD) and long non-coding transcripts (30% Ctrl; 34% LINE1-KD). However, LINE1-KD down-regulated transcripts showed a significant different repartitioning of transcripts. Of all down-regulated transcripts, 73% were protein-coding and only 13% were lncRNA transcripts (Chi-square test, observed versus expected, DF=4, *P* > 0.0001). Similarly, when evaluating the fraction of long (>100 kB) versus short genes (<100 kB) differentially regulated upon LINE1-KD, we found that down-regulated genes, but not up-regulated genes, were particularly long (≥100 kB). Compared with the fraction of long genes among all expressed transcripts in differentiated LUHMES neurons (9.69% of a total of 56,861 transcripts) or genes up-regulated upon LINE1-KD (10.61% of a total of 264 transcripts), down-regulated genes upon LINE1-KD belonged more than two times more frequently to the category of >100 kB genes (21.78% of a total of 698 transcripts) (Fig 8C and D). However, the probability of a long gene containing a flLINE-1 sequence to be differentially regulated by LINE-1 knockdown was not higher than for long genes not containing a flLINE-1 sequence. Along the same line, 59 of 671 down-regulated genes contained ≥1 young L1HS or L1PA2 element (8.79%), a frequency similar to the 19 of 254 up-regulated genes containing ≥1 young L1HS or L1PA2 element (7.48%).

**Figure S4.**
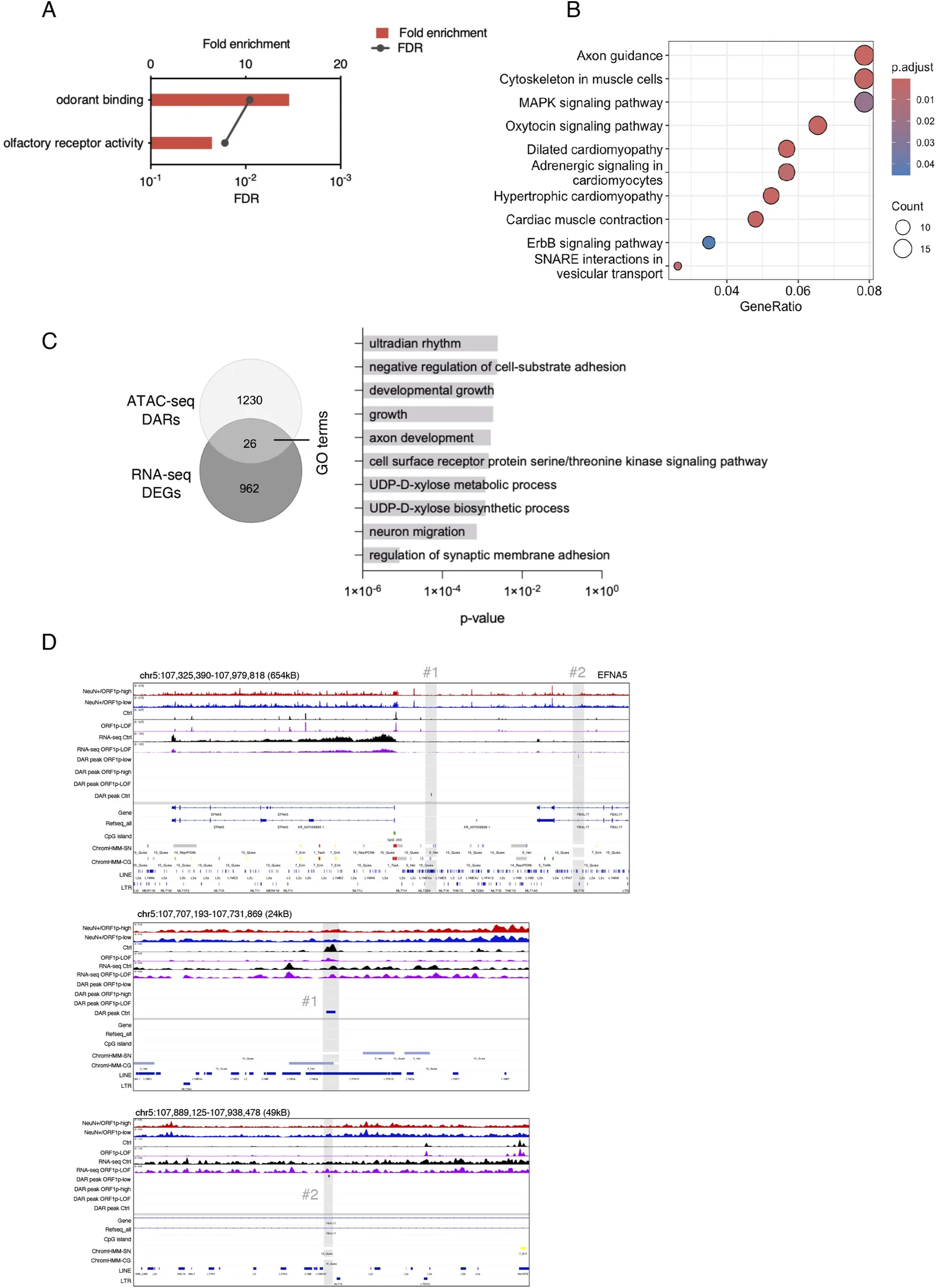
Comparaison of RNA-seq and ATAC-seq. results upon LINE1-KD in LUHMES. **(A)** GO analysis of significantly up-regulated transcripts in LINE1-KD neurons. FDR was calculated from *P*-values (Fisher’s exact test) by the Benjamini–Hochberg procedure. MF, molecular function. **(B)** Kyoto Encyclopedia of Genes and Genomes (KEGG) pathway analysis of significantly down-regulated genes upon LINE1-KD. *P*-value was calculated using a one-sided Fisher’s exact test, and the *P*-value was adjusted for multiple testing by the Benjamini–Hochberg method. **(C)** Venn diagram of common differentially accessible regions (DARs) in ATAC-seq (1,230 total) and differentially regulated genes (DEGs) in RNA-seq (962 total) from human neurons (LUHMES). GO (biological process, BP) of the 26 common genes. **(D)** IGV track of an example of the neuron-specific gene EFNA5 with DARs in human post-mortem cingulate gyrus (ORF1p-high versus ORF1p-low) and human LUHMES neurons (Ctrl versus LINE1-KD). EFNA5 was significantly down-regulated upon LINE1-KD.

**Figure S5.**
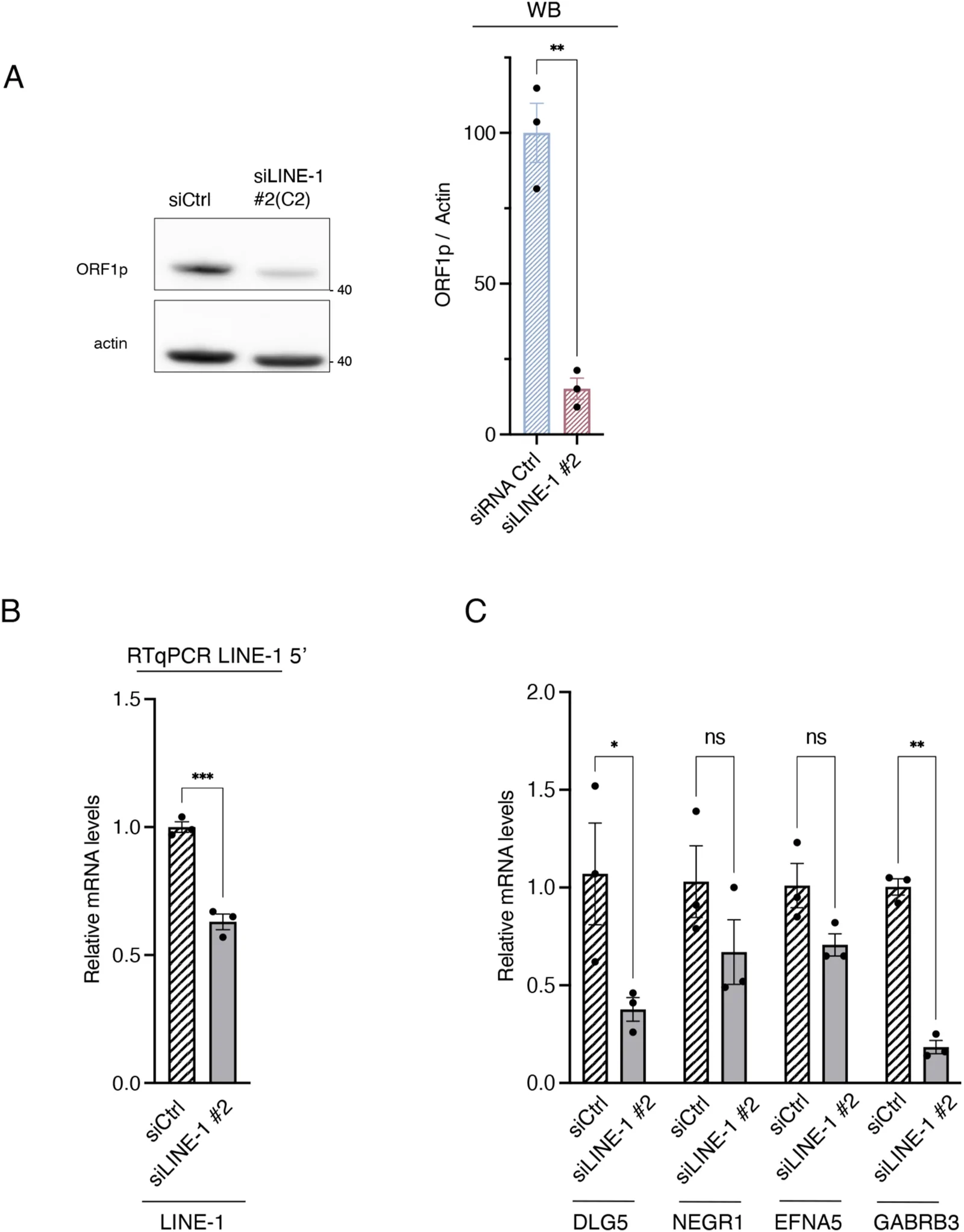
KD of LINE-1 in differentiated LUHMES using siRNA #2. **(A)** Western blot of ORF1p expression (left) and its quantification (right) upon LINE1-KD using LINE-1 siRNA #2 in mature LUHMES neurons. Unpaired *T* test, **P < 0.01, n = 3. **(B)** RTqPCR of LINE-1 expression in mature LUHMES neurons upon LINE1-KD using LINE-1 siRNA #2. Unpaired *T* test, ***P < 0.001, n = 3. **(C)** RTqPCR of gene expression of four genes related to neuronal functions and down-regulated using LINE-1 siRNA #1 (see Fig 6G) in mature LUHMES neurons upon LINE1-KD using LINE-1 siRNA #2. 2-way ANOVA, *P < 0.05, **P < 0.01, n = 3.

Together, this might indicate that LINE-1 is implicated in the regulation of long gene expression. Interestingly, long genes have been associated with neuron-specific functions (53, 54). GO analysis of up-regulated genes after LINE1-KD identified “odorant binding” and olfactory receptor activity as a significantly enriched (Fig S4A). More interestingly, down-regulated genes were enriched in highly significant GO terms related to synapse (FDR: 2,53e-011, FE: 2.32; GO CC), neuron projection morphogenesis (FDR: 0,000425, FE: 2,72; GO BP), nervous system development (FDR: 0,000275, FE: 1,73; GO: BP) and synapse organization (FDR: 0.0005, FE: 3,01; GO BP) (Fig 8E). PPI network analysis of significant down-regulated genes upon LINE-1 KD contained in the GO CC term “Synapse” (GO:0045202; 50 genes) using STRING with a stringent cutoff (log2 fold change ≤−0.5 / ≤−0.5, *P*-value ≤0.005) showed a highly interconnected protein interaction network (Fig 8F). A large proportion of these proteins were implicated in pre- and post-synapse, axon guidance, regulation of neurotransmitter levels, and synapse organization. In line with this, KEGG pathway analysis identified “axon guidance” as one of the most significant pathways enriched in down-regulated transcripts (Fig S4B). We independently tested four down-regulated genes with functions related to neurons by RTqPCR (*DLG5*, *NEGR1*, *EFNA5*, and *GABRB3*) and validated *DLG5*, *NEGR1*, and *EFNA5* to be significantly down-regulated after LINE1-KD (Fig 8G). To independently validate the LINE-1 KD-mediated down-regulation of neuron-related genes, we used an additional siRNA targeting LINE-1. Despite difficulties in selecting siRNAs knocking down LINE-1 ORF1p in LUHMES neurons, where the ORF1p protein seems to be particularly stable, we identified another LINE-1 siRNA which knocked down ORF1p efficiently as shown (left) and quantified (right) in Fig S5A using the same protocol as above. Using this LINE-1 siRNA ((siLINE-1 #2), LINE-1 RNA was significantly down-regulated (Fig S5B). We then tested this siRNA targeting LINE-1 in RTqPCR, selecting the same four genes we had identified as down-regulated in the RNA-seq experiment and in RTqPCR using siLINE-1 #1 as shown above (Fig 8). We confirmed the down-regulation of *DLRG5* and *GABRB3*, and there was a trend, but no significant down-regulation of the expression of *NEGR1* and *EFNA5* (Fig S5C). DARs identified in ATAC-seq are associated with the closest gene, although this might not always be biologically relevant as open chromatin regions, especially outside proximal promoter regions, do not necessarily regulate proximal genes but might act at greater distances. We nevertheless overlapped RNA-seq DEGs and ATAC-seq DARs obtained from LUHMES neurons and identified an overlap of 26 genes (Fig S4C). Interestingly, GO analysis of these genes (*APBB2*, *CACNA2D1*, *EVC*, *DUSP22*, *ENOX2,* *TMEM120B*, *ATF7*, *NMNAT2*, *USP13*, *SEPTIN9*, *SPATS2L*, *MDGA1*, *SMAD2*, *EFNA5*, *PSMB11*, *CPVL-AS2*, *UXS1*, *PPM1L*, *PCDH8*, *SDC3*, *PTBP2*, *PTPN3*, *MARCHF1*, *AUTS2*, *SATB1*, *TMTC2*) revealed that they belonged to neuron-specific pathways such as “axon development,” “regulation of synaptic membrane adhesion,” and “neuron migration” (Fig S4C). Interestingly, three of these genes with neuronal functions, namely, *AUTS2*, *PTBP2*, and *EFNA5*, also contained DARs in cingulate gyrus neurons. *AUTS2* (Activator Of Transcription And Developmental Regulator) is a component of the polycomb group multiprotein complex 1 with regulatory functions in neurite morphology and is associated with autism spectrum disorders, intellectual disability, and developmental delay (55). *PTBP2* (Polypyrimidine Tract Binding Protein 2) is predominantly present in the brain and involved in splicing regulation (56). *EFNA5* (Ephrin-A5) is involved in migration, repulsion, and adhesion, notably in axon guidance during neuronal development (57). The gene locus of *EFNA5* with ATAC-seq and RNA-seq tracks is shown in Fig S4D. Taken together, these transcriptomic changes upon LINE1-KD suggest a role of steady-state ORF1p in the homeostasis and maintenance of post-mitotic neurons.

### LINE-1 knockdown leads to changes in neurites morphology

**Figure 9.**
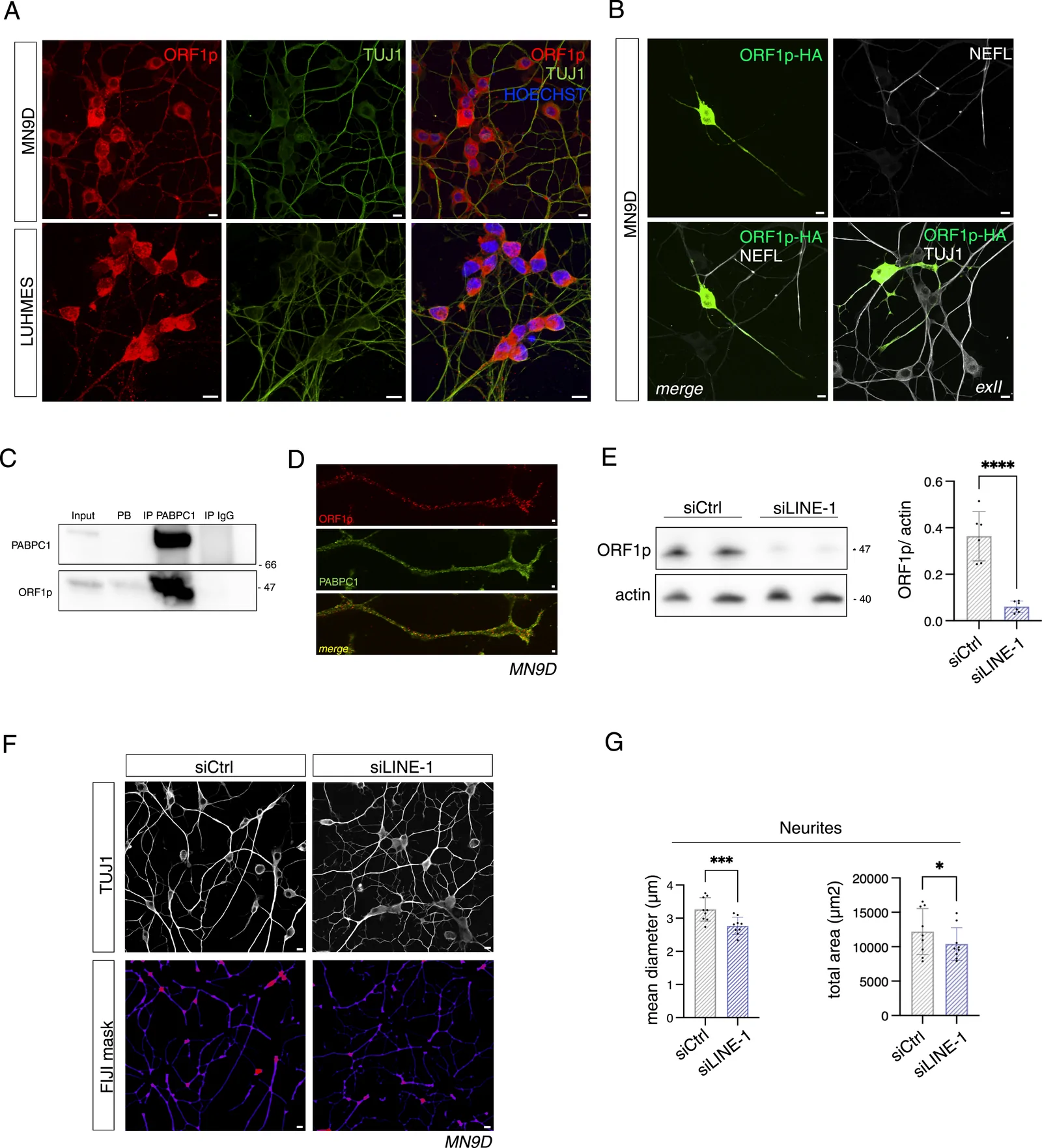
ORF1p knockdown induces phenotypic changes of neurites. **(A)** Representative images showing ORF1p expression (red) in neurites (green; TUJ1) in mouse (upper panel) and human (lower panel) dopaminergic neurons. Scale bar is 10 μm. **(B)** Expression of plasmid-encoded human ORF1p tagged with HA and labelled with a HA antibody (green) in differentiated mouse dopaminergic neurons (MN9D). Neurons were transfected at day 10 of differentiation. Neurites were counterstained with NEFL or TUJ1 (white) as indicated. ORF1p is readily detectable in TUJ1-positive neurites and NEFL-positive axons. Scale bar 10 μm. **(C)** Co-IP of ORF1p with PABPC1-antibody–coated beads. Western blot confirming efficient IP of PABPC1 and specific co-IP of ORF1p. **(D)** Immunofluorescence staining of ORF1p (red) and PABPC1 (green) in differentiated mouse neurons. Co-localization is visible in yellow. **(E)** Western blot analysis and quantification of ORF1p knockdown in differentiated MN9D (n = 6, one dot in the dot plot corresponds to one well; Mann–Whitney test, ****P ≤ 0.0001) (n = 3 independent experiments). **(F)** Immunofluorescent staining of the neuritic network of mouse dopaminergic neurons using TUJ1. siCtrl neurons are shown on the left and siLINE-1 neurons on the right. Scale bar is 10 μm. Masks were established using a Fiji-operated script for the quantification of neurite morphology. **(G)** Quantifications of neurite morphology based on TUJ1 staining of neurites. The mean diameter of neurites (in μm) was quantified using Fiji. Each point on the graph corresponds to the mean diameter value of an independent experiment. Neurite total area (μm2) was quantified in siCtrl and siLINE-1 MN9D neurons (n = 9 independent experiments, Paired *t* test, *P ≤ 0.05; ***P ≤ 0.001).

In addition to the fact that knockdown of LINE-1 induced chromatin accessibility and transcriptomic changes, which suggested that LINE-1 ORF1p (±LINE-1 RNA) might be involved in regulating the homeostasis and maintenance of adult neurons at steady-state, mass spectrometry analysis of protein interactors of endogenous ORF1p in both mouse and human neurons had revealed proteins related to neuron-specific functions with high confidence (cat.II ORF1p interacting proteins, Figs 1D, 2, 3, and Table S2). Cat.II ORF1p interactors belonged to GO categories like “neuron projections” (33 proteins in LUHMES, 65 in MN9D; 5 in common), “synapse” (42 proteins in LUHMES, 111 in MN9D, 15 in common), “dendrites” (18 in LUHMES, 43 in MN9D, 5 in common), and “axo-dendritic transport” (6 in LUHMES, 8 in MN9D, 1 in common; Table S1). Among these cat.II protein interactors of endogenous mouse and human ORF1p in neurons, 18 common proteins emerged; 9 ribosomal proteins and 2 myosin proteins with non-canonical functions in addition to YBX1, HNRNPU, MLF2, and FXR1 and 2 microtubule-associated proteins with canonical functions in neurite morphology, namely, two microtubule-associated proteins, MAP6 (dendrite morphogenesis, axonal growth and transport (58), and MAP1B (neurite extension (59)). These findings again suggest a possible role of ORF1p specific to neurons, which converge towards a pathway related to neurites (Fig 1D). Moreover, this idea was further supported by the presence of ORF1p itself in neurites as revealed by immunofluorescence stainings of endogenous ORF1p in MN9D and LUHMES (Fig 9A). Using TUJ1 to identify neurites, immunostaining of ORF1p showed its localization in neurites in both human and mouse dopaminergic neurons (Fig 9A). In addition, we transfected MN9D neurons with human ORF1p-HA and detected ORF1p by HA immunostaining, as expected, in the cytoplasm and the nucleus but also localized to neuronal extensions (counterstained with TUJ1), including axons (counterstained with NEFL) (Fig 9B). This indicated a possible colocalization of ORF1p with proteins related to neurite functions in the neuritic compartment itself. We verified the co-localization and co-IP of PABPC1, an interesting candidate with regard to neurite physiology (60, 61) and protective against dopaminergic neurodegeneration (62), which we had identified as interacting with ORF1p in both human and mouse neurons by mass spectrometry (Tables S1 and S2) and others in different cultured cell types (25, 26, 27, 28, 30, 31, 32, 33, 34). Co-IP (Fig 9C) and colocalization (Fig 9D) in mouse neurons validated the interaction and identified that both proteins colocalize in the cytoplasm but also in neurites. We thus hypothesized that both nuclear ORF1p-induced chromatin and transcriptional changes and ORF1p PPIs in the cytoplasm and in neuronal extensions might affect neurite morphology. To test this hypothesis, we carried out LINE1-KD in MN9D neurons at 7 d of differentiation and collected them at day 10 to perform neurite staining. LINE1-KD in mouse neurons was efficient as verified by Western blot (Fig 9E). Using an automated, non-biased image quantification workflow, we measured neurite morphology via measures of neurite diameter and area (Fig 9F and G). Quantifications of images illustrated in Fig 9F revealed a significant loss of the mean diameter of neurites after LINE1-KD accompanied by a decrease in the total neurite area (Fig 9G). In summary, these data suggest that LINE-1 ORF1p, in conjunction or not with LINE-1 RNA, plays a role in the maintenance of neurite morphology, possibly by converging pathways mediated by protein interactions and by modulating chromatin accessibility and transcriptomic landscapes.

## Discussion

Starting from the unexpected observation that the LINE-1–encoded ORF1p protein is expressed at steady-state in various neuronal subtypes in the adult brain of mice and humans (19, 20), we tested here the hypothesis that the sustained presence of ORF1p in an important fraction of neuronal cells in the brain (19) might be a requirement for neurons to maintain specific functions. Using quantitative mass spectrometry, confocal imaging, chromatin accessibility, and transcriptomics on human post-mortem neurons and differentiated mouse and human neurons in culture with varying levels of nuclear ORF1p, we provide evidence that ORF1p interacts with chromatin-binding proteins, binds to chromatin, modulates chromatin accessibility, and maintains neuronal gene expression. Loss of function of ORF1p leads to the down-regulation of genes related to neuronal processes and alters neurite morphology. In addition, ORF1p directly binds to proteins implicated in neurite morphology, indicating that it might be the converging interaction of ORF1p with proteins and chromatin that mediate this effect. To our knowledge, these data represent the first demonstration of a role of a LINE-1–encoded protein in the homeostasis of mature neurons and indicate a possible exaptation of a retrotransposon-encoded protein for physiological functions in the adult brain. These data thus add to the growing evidence for the exaptation of retrotransposon-derived RNA and proteins, for now documented during early development (2, 10, 11, 63), including in the developing brain (3). During early preimplantation development, LINE-1 RNA transcription leads to global chromatin relaxation and ribosomal DNA transcription, promoting blastocyst formation (10, 11). In premature aging cells, LINE-1 is up-regulated, and LINE-1 RNA acts as a negative regulator of the heterochromatin maintenance enzyme SUV39H1 (suppression of variegation 3-9 homolog 1). SUV39H1 dysfunction leads to heterochromatin loss and onset of aging phenotypes, which can be rescued by ASO-mediated depletion of LINE-1 RNA (64). Accumulating evidence also suggests that LINE-1 RNA interferes with the differentiation of the neuronal lineage. LINE-1 RNA was identified as a structural organizer of chromatin organization (65), activator of neuronal gene expression in ESCs (66 *Preprint*), in human neuronal progenitors (67) and cerebral organoids (12, 68), as well as in embryonic and adult neurogenesis (3) and as regulators of mouse brain corticogenesis (13). Importantly, Adami et al. reported that down-regulation of LINE-1 expression in *cis* in cerebral organoids using CRISPRi led to the up-regulation of genes linked to neuronal growth and neurogenesis and resulted in reduced cerebral organoid size (68). Mangoni *et al*. showed, using shRNA-mediated knockdown of LINE-1 in *trans,* that, in the developing mouse brain, LINE-1 acts as a long non-coding (lnc) RNA and as such epigenetically mediates the deposition of H3K27me3, leading to the down-regulation of genes implicated in neuronal differentiation and migration (13). Whereas it remains open whether, during corticogenesis, ORF1p is, in addition to LINE-1 RNA, involved in gene regulation, both sets of data suggest a function of LINE-1 RNA and/or LINE-1–encoded ORF1p in regulating parts of the neuronal transcriptome (13). Further, both studies link LINE-1 activity to neuronal gene expression regulation, either mediated through the binding of the LINE-1 RNA to polycomb complexes (13) or via nuclear ORF1p mediating differential accessibility on polycomb-repressed genome domains itself (this study). In post-mitotic neurons, both in human post-mortem and in LUHMES neurons, higher nuclear levels of ORF1p were associated with more accessible chromatin on weak polycomb repressive domains. Further, ORF1p nuclear content was associated with chromatin accessibility changes on the PRC-related gene *AUTS2*, and ORF1p interacted with the PRC1 protein CBX8. PRC1 is involved in chromatin silencing following PRC2-mediated trimethylation of histone H3 at lysine 27 (69, 70). Notably, Polycomb complex proteins are important for cellular identity in development (71) and in neuronal morphology (72) through the epigenetic silencing of target genes (73). LINE-1 RNA and PRC2 also interact outside the nervous system, maintaining nucleolar organization and an embryonic stem cell identity (23). The interaction of LINE-1 RNA with PRC2 or the differential accessibility of PRC chromatin domains depending on nuclear ORF1p content and the interaction of ORF1p with the PCR1 protein CBX8 could, thus, suggest a global and important role in maintaining cell identities, including the cell identity of post-mitotic neurons.

One important question which arises in this context is that of the molecular form of LINE-1 associated with the observed cellular and molecular changes upon knockdown, that is, whether ORF1p is acting alone, in association with LINE-1 RNA, with other cellular RNAs, and/or with ORF2p. As mentioned above, ORF1p is a nucleic acid chaperone and an RBP and is known to be because of its *cis*-preference, associated with LINE-1 RNA but also with other cellular RNAs prone to forming a LINE-1 RNP (74, 75, 76, 77, 78). We have shown here that ORF1p is present in the nucleus and bound to chromatin in post-mortem human neurons and in differentiated mature mouse and human neurons in culture, an observation that had been already made earlier in other cell types and in vitro (79, 80, 81). In dividing cells, the LINE-1 RNP is supposed to enter the nucleus passively when the nuclear envelope breaks down during mitosis (40, 81) before ORF1p re-exits the nucleus through an active process (40). In non-dividing somatic cells (35) and in G_o_-arrested cells like neurons (39), where retrotransposition events can occur, ORF1p supposedly enters the nucleus through binding to import proteins and active nuclear transport (38, 82), including in post-mitotic neurons (21). Whether once in the nucleus, ORF1p associates with RNA or not to bind chromatin, is an open question, which remains to be addressed. It is, however, possible that the presence of steady-state nuclear ORF1p could be a particular feature of post-mitotic neurons providing a unique nuclear function. As we have shown here, nuclear chromatin containing higher or lower levels of ORF1p showed differences in chromatin accessibility with similar characteristics in two different neuronal systems, suggesting a functional role of ORF1p on chromatin, which might or might not be involving (LINE-1) RNA. Whereas the overlap of genomic sites of differential chromatin accessibility in the cingulate gyrus and in LUHMES neurons was rather low, both systems recapitulated specific features of chromatin accessibility depending on nuclear ORF1p content. In both neuronal systems, high nuclear ORF1p led to more accessible chromatin on weak repressed polycomb domains and predominantly in distal intergenic regions, whereas low nuclear ORF1p was associated with more accessible chromatin over enhancers and flanking active transcription sites in intronic regions. The consistency between post-mortem findings and controlled in vitro experiments suggests a reproducible relationship between ORF1p and chromatin accessibility and provides cross-validation across different experimental systems, supporting the specificity of ORF1p′s effect on chromatin accessibility. Whether LINE-1 RNA might be involved or not remains to be investigated.

How exactly nuclear ORF1p can regulate chromatin accessibility remains to be investigated. One possibility is through the association with LINE-1 RNA or directly with proteins involved in chromatin-related functions. Indeed, we identified many nuclear proteins with chromatin-related function interacting with ORF1p. These protein interactors of ORF1p provide an extensive network of proteins related to chromatin organization and transcription regulation. Three protein interactors of ORF1p stood out as they were identified as common ORF1p-interacting proteins in both mouse and human neurons: RCOR2 (corepressor 2 of REST), a transcriptional repressor required for mouse ESC differentiation (83) and neurogenesis (84, 85), YTHDC1, a protein implicated in m6A-dependent RNA metabolism (86) and HNRNPU, a DNA and RNA binding protein associated with various neurodevelopmental disorders (87). HNRNPU is involved in chromatin organization, transcription regulation, and many other nuclear processes (88) and has been previously identified as an ORF1p interactor in cancerous cell lines (33). Interestingly, YTHDC1 has been shown to bind m6A-modified LINE-1 RNA to form a protein-RNA complex which represses the 2-cell program in ESCs (89) but might also be involved in neuronal survival (90) and cerebral stress response (91). It would be interesting to test the functional relevance of the interaction of ORF1p with these proteins in mediating chromatin accessibility in neurons. Whereas the functional impact of these PPIs remains to be determined, their identification reinforces the hypothesis that it might be ORF1p that modulates chromatin accessibility in mature neurons, either directly or indirectly through the binding to these proteins and/or to RNA. Given the extensive list of ORF1p protein interactors with functions in RNA metabolism, we cannot exclude that ORF1p might also regulate gene expression post-transcriptionally. Whereas many of these interactions were consistent with previoulsy identified protein partners of ORF1p in human cancer cell lines, mouse brain tissues, and sperm and were identified in well-established neuronal cell lines, future studies in primary neurons or brain tissue will be important to confirm their relevance in vivo and fully characterize the specificity and strength of these associations.

A combination of observations suggests a role of ORF1p in neuron-specific functions. (i) Shared genes containing DARs in both neuronal systems were enriched in gene categories related to axonogenesis and neuron projection. (ii) Transcriptomic changes observed after LINE1-KD indicated that down-regulated transcripts were particularly long, protein-coding, and related to neuron-specific functions like synapse, neuron projection, and axon guidance. (iii) Endogenous and HA-tagged ORF1p localized to neurites, both in mouse and human neurons. (iv) Mass spectrometry identified axono-dendritic ORF1p interacting partners. (v) Functionally, LINE1-KD induced morphological changes in neurites. Indeed, effects of LINE-1 on neurite morphology have been previously observed during mouse and human brain development. LINE-1 KD in vitro and in vivo induced morphological NPC differentiation and increased the number of dendritic branches, length, and complexities (14). This was accompanied by LINE-1-KD DEGs enriched in functional categories including morphogenesis and projection, similar to what we identified here. Morphological changes during neurodevelopment were characterized by an increase in neurite branches, whereas we observed a decrease in neurite diameters. This suggests that flLINE-1 activity acts on neuronal morphology in post-mitotic neurons. Interestingly, whereas Toda et al. did detect LINE-1 RNA on chromatin, there was no correlation of the presence of RNA and DEG gene loci as described during zygotic development (11), which could suggest that the presence of ORF1p is mandatory for specificity in chromatin targeting in neurons. Indeed, during preimplantation development, ORF1p is exclusively cytoplasmic (11), whereas in neurons, ORF1p is readily detectable in the cytoplasm and the nucleus at steady-state. These data suggest a dual role of ORF1p converging on the regulation of specialized neuronal functions. Whereas future work will be required to demonstrate the direct causal links between these sequential events, the consistent enrichment for neuron-specific biological processes such as ‘axon guidance’ in the proteomic, chromatin, and transcriptomic analyses provides strong correlative support for a unified functional pathway.

Although we have provided correlative genomic and loss of function evidence for an involvement of ORF1p in adult neuronal physiology, our study has some limitations. We cannot definitively exclude that the observed effects are partially mediated by LINE-1 RNA or other indirect effects or whether ORF1p is bound to LINE-1 RNA on chromatin. The identification of ORF1p chromatin-binding sites, the characterization of the ORF1p complex binding chromatin, and/or rescue experiments of ORF1p should be the subject of future studies.

Together with mounting evidence of the importance of LINE-1 activity during embryonic and adult neurogenesis (2) and neurodevelopment (3), our work, thus, contributes to the increasing evidence of the exaptation of what was once called “selfish elements” for important physiological brain functions and beyond. This is important to take into consideration when targeting LINE-1 activity for developing novel therapies for diseases like cancer, autoimmune, and neurodegenerative diseases. We have shown previously that excessive ORF1p and its translocation to the nucleus alter nuclear membrane integrity, transport, and heterochromatin organization (21), indicating that increased nuclear levels of ORF1p might be deleterious. Our present study adds to the understanding of LINE-1 biology by showing that mature neurons depend on LINE-1 ORF1p for the fine-tuning of chromatin organization, neuron-related gene expression, and neurite morphology. Taken together, we propose a threshold model in which intermediate levels of ORF1p are necessary for maintaining neuronal morphology and function, whereas levels going below or beyond a physiological steady-state expression window might be harmful to neurons.

## Materials and Methods

### Ethical approvals

Reglementary authorizations to use human cell lines and human samples were obtained from the French Ministry of Higher Education, Research, and Innovation (authorization number DC-2020-4013). Informed consent was obtained from all human subjects and experiments conformed to the principles set out in the WMA Declaration of Helsinki and the Department of Health and Human Services Belmont Report. Experiments using siRNA in human cells (LUHMES) were authorized according to regulatory procedures defined by the French Ministry of Higher Education, Research, and Innovation (OGM n°8273 and OGM n°10463).

### Cell culture

LUHMES cells were ordered from ATCC (ATCC-CRL-2927). Cell culture dishes were pre-coated with 50 μg/ml poly-L-ornithine (#P4957; Merck) diluted (1:2) in DPBS (#14190144; Gibco) with, in addition, 1 μg/ml human plasma fibronectin (#F0895; Sigma-Aldrich) at 37°C for at least 3 h and then rinsed 2 times with DPBS. Proliferating LUHMES were cultured using Advanced DMEM/F12 (#12634028; Gibco) added with 1% N-2 supplement (#15410294; Gibco), GlutaMax (#35050061; Gibco), and 40 ng/ml human recombinant FGF (#100-18B; Peprotech) at 37°C in a 5% CO_2_ incubator. LUHMES cells were differentiated into mature dopaminergic neurons. For this purpose, neurons were cultured in advanced DMEM/F12 added with 1% N-2 supplement and GlutaMax, supplemented with 1 μg/ml doxycycline (Sigma-Aldrich), 2 ng/ml recombinant human GDNF (#450-10; Peprotech), and 1 mM cAMP (#D0627; Sigma-Aldrich). ORF1p IP was performed on day 5 of differentiation, and all ORF1p knockdown experiences were conducted on day 7 of differentiation, when LUHMES cells are mature dopaminergic neurons (92, 93). The medium was changed every 2 d. Cells used in this study had a maximum of 15 passages.

The MN9D cell line was purchased from Merck (# SCC281). Cell culture plasticware was coated with 1 mg/ml poly-lysine (#P4707; Merck) for 2 h and then rinsed two times with DPBS. Cells were cultured in DMEM High Glucose (#D5796; Sigma-Aldrich) supplemented with 10% fetal bovine serum (FBS) (#10270098; Gibco) in 5% CO_2_, 37°C incubator. MN9D were differentiated for 10 d with the following medium: DMEM High Glucose (#D5796; Sigma-Aldrich) supplemented with 10% fetal bovine serum (FBS) (#10270098; Gibco) with 1 mM n-butyrate (#B5887; Sigma-Aldrich) and 1 mM dibutyryl cAMP (#D0627; Sigma-Aldrich). Media were changed every 2 d. Experiences were performed on day 10 of differentiation. Cells used in this study had a maximum of 15 passages.

### siRNA delivery

Around 150,000 cells/well of LUHMES or MN9D were plated. Media was freshly replaced 2 h before. Cells were lipofected using RNAiMAX (#13778150; Invitrogen) at day 3 of differentiation for LUHMES and day 7 of differentiation of MN9D, with 100 nM of siRNA. The target sequence of the human ORF1 siRNA was the same as in the shRNA ORF1 sequence characterized by Philippe et *al*. (94), named #1. Alignment to full-length and coding LINE-1 sequences in the human reference genome (hg38, as defined by REF Penzkofer et *al*., L1Basev2) showed the presence of this target sequence in 134 of 146 LINE-1 elements (L1HS and L1PA2). The second siRNA against LINE-1 ORF1 was also taken from Philippe et *al*. (94) (named #2).

siCtrl: TAA​TGT​ATT​GGA​ACG​CAT​A

siLINE-1 (93) (#1, human LINE-1, ORF1, LUHMES, target sequence):

5′-AAG​AAG​GCT​TCA​GAC​GAT​CAA-3′

siLINE-1 (#2, human LINE-1, ORF1, LUHMES, target sequence):

5′-AAA​TGA​AGC​GAG​AAG​GGA​AGT-3′

siLINE-1 (20) (mouse LINE-1, ORF2-MN9D, target sequence): CTA​TTA​CTC​TGA​TAC​CTA​AAC.

### Plasmid delivery

MN9D were plated at the density of 200,000 cells/well into a pre-coated 4-well plate. On day 7 of differentiation, new prewarmed media were added 2 h before, and cells were transfected with 1 μg of plasmid/well (pcDNA3-ORF1-HA, kind gift from Dr. John and Dr. Ariumi (95)) or pmaxGFP (Lonza) using Lipofectamin 3000 (#L3000015; Thermo Fisher Scientific) according to the manufacturer’s instruction.

### Antibodies

All antibodies used in imaging, Western blotting, and FANS experiments are listed in Table S4.

Table S4. List of all antibodies used in this study.

### Western blot

Cells were scraped at day 7 of differentiation (LUHMES) and at day 10 of differentiation (MN9D) in RIPA buffer (10 mM Tris–HCl pH 8.0, 150 mM NaCl, 1 mM EDTA, 1% Triton X-100, 0.1% sodium deoxycholate, 0.1% SDS). Laemmli buffer was added, and samples were boiled for 10 min at 95°C before loading on a 1.5 mm NuPAGE 4-12% Bis-Tris polyacrylamide gel (#NP0336BOX; Invitrogen) for the Western blotting. Gels migrated in NuPAGE MES SDS Running Buffer (#NP0002; Invitrogen) for 1h 10 min at 175 mV. Gels were transferred onto a methanol-activated PVDF membrane (# IPVH00010; Immobilon) in transfer buffer (25 mM Tris pH: 8.3 and 192 mM glycine) during 1 h 30 at 400 mA. Membranes were blocked in 5% milk TBST (0.2% Tween 20, 150 mM NaCl, 10 mM Tris pH: 8) for 1 h. Primary antibodies were diluted in 5% milk in TBST, and membranes were incubated o/n at 4°C. After 3 × 10 min washing in TBST, membranes were incubated for 1 h with the respective secondary antibodies diluted at 1/2,000 in 5% milk TBST, followed by 3 × 10 min washes in TBST. Membranes were revealed by the LAS-4000 Fujifilm system using Clarity Western ECL Substrate (Bio-Rad) or Maxi Clarity Western ECL Substrate (Bio-Rad) according to the sensitivity needed. Quantifications were performed using LI-COR Image Studio Lite (v.5.2.5).

### Immunofluorescence

Cells were fixed using 4% methanol-free formaldehyde (#28908; Sigma-Aldrich) diluted in PBS for 20 min at RT and washed three times with PBS. Cells were permeabilized with 0.5% Triton X-100 PBS 20 min and washed twice with PBS. 100 mM glycine was added for 20 min, and after washing three times with PBS, cells were incubated with blocking buffer (10% FBS, 0.5% Triton X-100, PBS) for 1 h. Primary antibodies in blocking buffer were added overnight at 4°C. The next day, following three washes with PBS for 10 min, cells were incubated with secondary antibodies diluted in blocking buffer for 1 h at RT. Finishing with three PBS washes, slices were mounted with Fluoromount (#00-4958-02; Invitrogen). Fluorescence images were acquired using a CSU-W1 spinning-disk confocal system (Yokogawa) on an Axio Observer Z1 inverted microscope (Zeiss) and equipped with an Orca Flash 4.0 sCMOS camera (Hamamatsu Photonics) or a CSU-X1 spinning-disk confocal unit (Yokogawa) on a Nikon Eclipse Ti inverted microscope, equipped with an Orca Flash 4.0 sCMOS camera (Hamamatsu Photonics). Objectives used were either a 40×/1.40 NA, a 63×/1.40 NA Plan-Apochromat oil immersion or 100×/1.40 NA oil immersion with 405, 491, 561, and 642 nm laser lines.

### Image analysis

Neurite network analysis was conducted using a custom macro developed for the Fiji software (96), incorporating functionalities from the Bio-Formats (97), Analyse Skeleton (98), and Local Thickness (99) plugins. Code is openly accessible at https://github.com/orion-cirb/Axon_Skel_Analyzer.

Nuclei were segmented and counted in the DAPI channel using the following sequence of operations: maximum intensity z-projection, median filtering (radius = 10 pixels), Huang automatic thresholding, hole filling, watershed splitting, object labelling, and filtering based on morphological criteria (minimum area = 40 μm^2^, minimum circularity = 0.7). Cell bodies were segmented in the ORF1p channel by successively applying sum slices z-projection, median filtering (radius = 50 pixels), Huang thresholding, morphological opening (20 iterations) and dilation (5 iterations), hole filling, and size filtering (minimum area = 80 μm2).

The TUJ1 channel was segmented via maximum intensity z-projection, background subtraction (rolling ball radius = 50 pixels), median filtering (radius = 2 pixels), Huang thresholding, morphological closing (4 iterations), additional median filtering (radius = 2 pixels), selective hole filling (holes <10 μm^2^), and size filtering (minimum area = 20 μm2). To isolate neurites, cell bodies segmented from the ORF1p channel were subtracted from the TUJ1 channel binary mask. The resulting neuronal processes were then skeletonized, and branches shorter than 4 μm were excluded to minimize artifacts. This processing pipeline enabled local thickness analysis to quantify the distribution of branch diameters.

The analysis of ORF1p intensity after LINE1-KD in LUHMES cells was performed using the Nuclei_ORF1p plugin (21) developed for the Fiji software. This tool enabled quantification of ORF1p signal specifically in the nucleus versus the cytoplasm of cells.

### Subcellular fractionation

Subcellular fractionation was performed with 10^6^ cells using the Subcellular Protein Fractionation kit (#78840; Thermo Fisher Scientific) according to the manufacturer’s recommendations. For cytoplasm and nucleus fractionation, cells were washed in PBS and resuspended in lysis buffer (10 mM HEPES ph 7.9, 40 mM KCL, 5% glycerol, 3 mM MgCl2, 0.2% NP40, EDTA-free protease inhibitor cocktail, and phosphatase inhibitors) and incubated for 30 min at 4°C. Lysates were passed 20 times in a Dounce homogenizer (loose) and then left for 30 min on a wheel at 4°C. After centrifugation at 4°C at 500 *g* for 5 min, the supernatants were kept as the cytosolic fraction, and the pellets were washed once in lysis buffer and resuspended in nuclear lysis buffer (20 mM HEPES ph 7.9, 420 mM NaCl, 25% glycerol, 1.5 mM MgCl2, EDTA-free protease inhibitor cocktail, and phosphatase inhibitors) and rotated for 30 min on a wheel at 4°C. Finally, lysates were centrifuged for 10 min at 14,000 *g* at 4°C, and the supernatant was collected as nuclear extract.

### RNA extraction

3.5 × 10^5^ cells were scrapped in TRIzol reagent (#15596026; Invitrogen), and total RNA was extracted using Direct-zol RNA Microprep (#R2062; Zymo) according to the manufacturer’s instructions. All samples were subjected to DNAse I treatment during extraction.

### RTqPCR

300 ng RNA was subject to reverse transcription using the All-In-One 5X RT MasterMix kit (#G592; Abm). qPCR was performed in duplicate for each sample and each gene with SsoAdvanced Universal SYBR Green (#1725274; Bio-Rad) with the following protocol: a first heat-up at 95°C, followed by the denaturation step at 95°C, annealing at 57°C, and finally extension at 72°C for a total of 40 cycles, on a CFX Opus 384 Real-Time PCR System (#12011452; Bio-Rad). qPCR was analysed by CFX Maestro 2.0 software (v5.0.021.0616) (Bio-Rad), and each gene of interest was normalized to three housekeeping genes: ENO2, TBP, and HPRT.

The following primers were used: HPRT (F: CAG​CCC​TGG​CGT​CGT​GAT​TAG​T; R: CCAGCAGGTCAGCAAAGA AT); TBP (F: CAG​CAT​CAC​TGT​TTC​TTG​GCG​T; R: AGA​TAG​GGA​TTC​CGG​GAG​TCA​T); ENO2 (F: CGT​TAC​TTA​GGC​AAA​GGT​GTC​C; R: CTC​CAG​CAT​CAG​GTT​GTC​CAG​T); *DLG5* (F: GCC​TTC​AGA​GTC​TGC​CTT​GT; R: CAG​GCT​GGA​AGA​CAG​GAA​GG); *NEGR1* (F: TTA​AGA​GGC​AGG​CTC​GCA​TT; R: TAC​CAA​CGT​GAC​ACA​GGA​GC); *EFNA5* (F: AGC​GGT​CCA​TTT​GGA​GAG​TG; R: ATG​AGG​ACT​CCG​TCC​CAG​AA); *GABRB3* (F: GCC​TTG​GCT​GTC​TTT​TCT​GC; R: ACC​TTA​TGG​GCT​GCT​TCG​TC).

### IP and sample preparation for mass spectrometry

Magnetic beads were coupled with ORF1p (MN9D: #ab245124; Abcam) (LUHMES: #ab245249; Abcam) or IgG rabbit (#ab172730; Abcam) antibodies using the Dynabeads Antibody Coupling Kit (#14311D; Thermo Fisher Scientific) according to the manufacturer’s recommendations. After two washes in DPBS, cells were scraped in lysis buffer (10 mM Tris HCl pH: 8, 150 mM NaCl, 0.5% NP40, protases, and phosphatase inhibitors), incubated on ice for 15 min, and sonicated (Bioruptor UCD-200) for 15 min (30 s on/30 s off) on ice at medium power. Lysates were incubated with coupled beads and diluted in wash buffer (10 mM Tris HCl pH8, 150 mM NaCl, protases, and phosphatase inhibitors) overnight on a wheel at 4°C. Samples were washed four times with wash buffer and were resuspended in 100 μl of 25 mM NH4HCO3. IP samples were then washed twice with 100 μl of 25 mM NH4HCO3 (ABC), and proteins were trypsin/lysC (0.2 μg, Promega) digested directly on beads in a total volume of 100 μl of 25 mM ABC for 1 h at 37°C while under agitation at a constant speed of 1,000 rpm in a thermomixer. Samples were then cleaned on homemade C18 StageTips (AttractSPE Disk Bio C18-100.47.20 Affinisep) before being eluted using 40/60 CH3CN/H2O + 0.1% formic acid and vacuum concentrated to dryness.

### LC-MS/MS analysis

LUHMES liquid chromatography was performed with an RSLC nano system (Ultimate 3000, Thermo Fisher Scientific) coupled online to an Orbitrap Exploris 480 mass spectrometer (Thermo Fisher Scientific). Peptides were trapped on a C18 column (75 μm inner diameter × 2 cm; nanoViper Acclaim PepMap 100, Thermo Fisher Scientific) with buffer A (2/98 CH3CN/H2O in 0.1% formic acid), and separation was performed on a 50 cm × 75 μm C18 column (nanoViper Acclaim PepMap RSLC, 2 μm, 100 Å, Thermo Fisher Scientific) with a linear gradient of 3% to 29% buffer B (100% CH3CN in 0.1% formic acid, A’: 100% H2O in 0.1% formic acid) at a flow rate of 300 nl/min over 91 min. For data-dependent acquisition (DDA), MS full scans were performed in the ultrahigh-field Orbitrap mass analyser in the ranges m/z 375–1,500 with a resolution of 120,000 at m/z 200. The top 20 intense ions were subjected to Orbitrap for further fragmentation via high-energy collision dissociation (HCD) activation and a resolution of 15,000 with the intensity threshold kept at 1.3 × 10^5^. We selected ions with charge states from 2+ to 6+ for screening. Normalized collision energy was set at 27 and the dynamic exclusion at 40 s.

MN9D LC-MS/MS analysis was performed, as previously, in data-independent acquisition (DIA) mode and by using a linear gradient of 2% to 30% buffer B. MS full scans were performed in the ultrahigh-field Orbitrap mass analyser in the range m/z 375–1,500 (120,000 resolution; IT 100 ms; AGC 300%). 40 DIA scan of a 15 Da isolation window with a 1 Da overlap between 400 and 1,000 m/z (15,000 resolution; normalized collision energy 25; AGC 3000; IT auto) were set.

For protein identification, LUHMES DDA data were searched against the *Homo sapiens* (UP000005640_9606) UniProt database using Sequest HT through proteome discoverer (PD version 2.4). Enzyme specificity was set to trypsin and a maximum of two miss-cleavage sites were allowed. Oxidized methionine, Met-loss, Met-loss-Acetyl, and N-terminal acetylation were set as variable modifications. Maximum allowed mass deviation was set to 10 ppm for monoisotopic precursor ions and 0.02 Da for MS/MS peaks.

MN9D DIA data were searched against the Mus musculus (UP000000589) Uniprot database using Spectronaut v18.6 (Biognosys) by directDIA+ analysis using default search settings. Enzyme specificity was set to trypsin and a maximum of two missed cleavage sites were allowed. N-terminal acetylation and oxidation of methionine were set as variable modifications.

The resulting files were further processed using myProMS v3.10.0 (https://github.com/bioinfo-pf-curie/myproms) (100). FDR calculation used Percolator (101) and was set to 1% at the peptide level for the LUHMES data. The label-free quantification was performed by peptide extracted ion chromatograms (XICs), and DDA were reextracted by conditions and computed with MassChroQ version 2.2.21 (102) (LUHMES data). For protein quantification, XICs from proteotypic peptides shared between compared conditions (TopN matching) with missed cleavages were used. Median and scale normalization at the peptide level was applied to the total signal to correct the XICs for each biological replicate (N = 5). To estimate the significance of the change in protein abundance, a linear model (adjusted on peptides and biological replicates) was performed, and a two-sided *t* test was applied on the fold change estimated by the model. The *P*-values were adjusted using the Benjamini–Hochberg FDR procedure.

The mass spectrometry proteomics raw data have been deposited to the ProteomeXchange Consortium via the PRIDE (103) partner repository under the accession number PXD066047.

### RNA sequencing and analysis

Four samples for each condition (siRNA-Ctrl and siRNA-Orf1) were sequenced (Novogene Europe). Quantity and quality were assessed using Nanodrop and Bioanalyzer RNA 6000 Pico Kit (#5067-1513; Agilent Technologies). Libraries, including rRNA removal, were generated by the RNA Library Prep Kit for Illumina following the manufacturer’s recommendations and sequenced as pair-end (PE) 150 bp using the Novaseq x plus sequencer. Mapping was performed by Hisat2 (v2.0.5) on the human genome (hg38/GRCh38 assembly). Differential enrichment analysis was performed with DESeq2 (v1.46.0), and data were normalized using the DESeq2 scaling and size factors method. Genes where the sum of reads from all samples was under ten reads were removed. Genes with no reads for at least 6 samples on 8 were discarded. Volcano plot was generated by considering as significatively up-regulated (264 genes), genes with log2 fold change ≥0.5 and *P* ≤ 0.01; significatively down-regulated (698 genes), genes with log2 fold change ≤−0.5, *P* ≤ 0.01). Another more stringent threshold was fixed for defining down-regulated genes (log2 fold change ≤−0.5, *P* ≤ 0.005), and among those, genes forming a network involved in synapse GO (50 genes) were visualized by the STRING database (v.12.0). Gene annotations were retrieved from hg38 RefSeq_all, and long genes were defined as >100 kB. Kyoto Encyclopedia of Genes and Genomes (KEGG) pathway analysis was conducted with the R function enrichKEGG from clusterProfiler (v.4.14.4). Gene Ontology (GO) analysis was executed using the PANTHER GO database (release 19.0). *P*-values were adjusted using the Benjamini–Hochberg method. To analyse TEs, adapters were trimmed using TrimGalore (v0.6.10) (104). Paired-end read alignment was performed onto the human reference genome (hg38) with STAR (v2.7.6a) [2], allowing at most 6% of mismatches (--outFilterMismatchNoverLmax 0.06) and reporting randomly one position among at most 5,000 positions for a read (--outFilterMultimapNmax 5000 --outSAMmultNmax 1). The repeat annotation was downloaded from the UCSC Table Browser using RepeatMasker rmsk track for hg38. Quantification was performed with FeatureCounts (v2.0.1, parameters: -M -p -C -s 0).

### Human samples

Cingulate gyrus brain tissue from control (n = 5) and Parkinson disease patients (n = 5) were provided by the Biobank Neuro-CEB and conserved at −80°C. Tissues underwent one manual physical shock and were then disposed of in precooled Tissue-Tube bags (Covaris) and dry cryo-pulverized with a level 6 impact using the CryoPrep Dry Pulveriser (Covaris). All steps were performed on dry ice.

### Isolation and sorting of nuclei (FANS)

Nuclei from pulverized tissues of human patients were resuspended in homogenization buffer (0.25 M sucrose, 0.5 mM spermidine, 0.15 nM spermine, NP40 0.3%, protease inhibitors, 1 mM DTT). Tissues were grinded mechanically using a Dounce homogenizer and passed through a 40 μm cannula. The recovered cells were then put on an OptiPrep gradient (#D1556; Sigma-Aldrich) and centrifuged for 15 min at 10,000 *g*. Pellets were dried and put in PBS spermine 0.15 nM. Human nuclei were then labelled 1 h on a wheel at 4°C using the following antibodies: NeuN PE (#FCMAB317PE; Merck), ORF1p AF647 (#ab314927; Abcam), and control antibodies: IgG1 mouse-PE (#A07796; Beckman Coulter) and IgG rabbit-AF647 (#ab199093; Abcam). Nuclei sorting was performed using a FACSARIA II cell sorter (Beckton Dickinson) equipped with three lasers (405, 488, and 640 nm), a 70 μm nozzle, and FACSDiva software (version 6.1.2). Nuclei were detected according to their forward scattering light and gated using forward and side scatter light signals. Aggregates were excluded by forward scatter area versus height pulse measurement. Finally, PE and AF647 fluorescence were, respectively, measured through 585/42 and 660/20 nm bandpass filters. P3 corresponds to all nuclei (defined as ORF1p-high), which fluoresce beyond the maximum intensity of nuclei in the control (non-relevant antibody that fluoresces in the same channel as ORF1 [x-axis, 647 nm]), whereas P4 is everything below this threshold (ORF1p-low nuclei). As in the control (non-relevant antibody that fluoresces in the same channel as NeuN [y-axis, NeuN-PE]), autofluorescent nuclei form a diagonal, we excluded nuclei in this range and defined as NeuN-positive all nuclei above this autofluorescent window. 50,000 nuclei were recorded. Nuclei were collected in PBS buffer in Eppendorf tubes.

### ATAC sequencing and analysis

After FANS, 50,000 nuclei per condition were centrifuged at 4°C, 500 *g* for 10 min. ATAC-seq was then performed using a commercial kit (Diagenode Cat. No. C01080006 for human tissue and Cat. No. C01080001 for LUHMES) according to the instructions of the manufacturer. Profiles of libraries were verified using the Bioanalyzer High Sensitivity DNA Kit (Agilent Technologies). The library pool was quantified by qPCR using the KAPA library quantification kit (Roche). Sequencing was carried out on the NovaSeq 6000 instrument from Illumina using paired-end 2 × 100 bp sequencing. ATAC-seq data were analysed using the Curie Institute ATAC-seq pipeline (v1.02, DOI: 10.5281/zenodo.7576558 and available at https://github.com/bioinfo-pf-curie/ATAC-seq/releases/tag/v1.0.2). Briefly, sequencing reads were aligned after adapter removal on the human reference genome (hg38) with bwa-mem. Aligned reads were filtered to remove mitochondrial reads, PCR duplicates, singleton, and reads aligned with a mapping quality lower than 20. Genomic sites from the ENCODE blacklist regions were also discarded from the analysis. The peak calling was then performed using the MAC2 software with the following parameters: “-f BAMPE --nomodel -q 0.01.” The DeepTools (v3.5.4) command multiBamSummary was used to count reads from all peaks. All peaks were kept, and peaks with the sum of reads from all samples smaller than ten reads were removed. Differential accessible peaks were determined with DESeq2 (v1.46.0). For LUHMES, we compared siRNA-Ctrl (n = 2) and siRNA-ORF1 (n = 2) and peaks were considered significant with a *P*-value ≤0.05 and log2FC ≥ and ≤0.5. For human samples (NeuN− = 4 samples, NeuN+ ORF1p-low = 6 samples, and NeuN+ ORF1p-high = 6 samples), peaks were considered significant with a *P*-value ≤0.01 and log2FC ≥ and ≤1. Peaks were annotated to the nearest gene with the function annotatePeak from the ChIPseeker R package (v.1.42.1), using *TxDb.Hsapiens.UCSC.hg38.knownGene* and TSSregion set to ±3 kb. Motif enrichment analysis was performed with HOMER (v5.1), using findMotifsGenome.pl, with default parameters. GO analysis was conducted on the PANTHER GO database (release 19.0). A significative DAR association with known chromatin states was investigated using available ChromHMM annotations (retrieved from Roadmap: https://pmc.ncbi.nlm.nih.gov/articles/PMC8734071/) from adult cingulate gyrus (ATAC human brain) and substantia nigra (ATAC LUHMES). 71 significant genes were overlapping between ATAC-seq in human post-mortem tissues and LUHMES. GO BP analysis of those proteins was carried out in R using clusterProfiler (v.4.14.4) package and represented as a GOChord plot (package GOplot v.1.0.2). Bigwig tracks were visualized using the Integrated Genome Viewer (IGV) browser (v2.19.1). To analyse whether ATAC peaks were overlapping with L1HS and L1PA2, repeat annotation was downloaded from the UCSC Table Browser using the RepeatMasker rmsk track for hg38.

### Statistics

Statistical analyses were performed with Graph Pad PRISM software (v10.4.1). The significance threshold was defined as *P* < 0.05 except stated otherwise. We assessed the distribution of DARs across genomic features or regulatory regions using two-sided Fisher’s exact tests. To ensure statistical robustness, categories with low total counts (<5 DARs) were excluded from these analyses. False discovery rate (FDR) correction with reported q-values was applied to *P*-values of the remaining comparisons using the Benjamini–Hochberg procedure (Q =1%). Gene biotype and long gene overrepresentation analyses were performed using a Chi-square test with observed = number of feature counts in LINE1-KD and expected = all RNA-seq transcript counts.

## Supplementary Material

Reviewer comments

## Data Availability

The ATAC-seq and RNA-seq data stemming from human differentiated neurons in culture (LUHMES) are available at EBI-EMBL ArrayExpress database under the following accession numbers: ATAC-seq: E-MTAB-15871 RNA-seq: E-MTAB-15856 The mass spectrometry raw data are available on the PRIDE database with the accession number PXD066047. The ATAC-seq data stemming from post-mortem human brain tissue are only available upon request from the authors because of privacy and ethical restrictions.
