# Supplementary material for: Interactions of LINE-1 ORF1p with proteins and chromatin suggest a role in neuronal physiology
Source: Life Sci Alliance. 2026 Aug 21;9(11):e202503546. doi: 10.26508/lsa.202503546 (PMC13498733; doi:10.26508/lsa.202503546)
Supplement: Supplementary file 1 [file LSA-2025-03546_review_history.pdf]

Interactions of LINE-1 ORF1p with proteins and chromatin suggest a role in neuronal physiology

Sandra Sinnassamy, Olivia Massiani-Beaudoin, Bérangère Lombard, Damarys Loew, Tom Bonnifet, Magali Fradet, Héloïse Monnet, Thomas Caille, Nicolas Servant, Rajiv Joshi, and Julia FUCHS
DOI: https://doi.org/10.26508/lsa.202503546

Corresponding author(s): Julia FUCHS, Collège de France and Rajiv Joshi, Collège de France

Review Timeline:		
	Submission Date:	2025-10-24
	Editorial Decision:	2025-12-22
	Revision Received:	2026-06-02
	Editorial Decision:	2026-07-10
	Revision Received:	2026-08-05
	Accepted:	2026-08-10

Scientific Editor: Sarita Hebbar

Transaction Report:

(Note: With the exception of the correction of typographical or spelling errors that could be a source of ambiguity, letters and reports are not edited. The original formatting of letters and referee reports may not be reflected in this compilation.)

December 22, 2025

Re: Life Science Alliance manuscript #LSA-2025-03546-T

Dr. Julia FUCHS
Collège de France
Centre for Interdisciplinary Research in Biology (CIRB)
11, place Marcelin Berthelot
Paris 75231 Paris Cedex
France

Dear Dr. Fuchs,

Thank you for submitting your manuscript entitled "Interactions of LINE-1 ORF1p with proteins and chromatin converge on a role in neuronal physiology" to Life Science Alliance.

Your manuscript was evaluated by three expert reviewers whose comments are appended below. All three reviewers agree that this work is of potential interest to the field. You will also note that the reviewers had several concerns that preclude publication at this stage.

We agree with all the reviewers that the conclusion on ORF1p's influence on the observed effects is overstated. The reviewers all state potential other possibilities that underlie these observations which must be addressed in the manuscript text. Further, we encourage you to provide evidence to back these strong claims; for example suggestions by Reviewer 3 to eliminate a role of LINE-1 RNA or to rule out collateral effects. In the absence of any additional supporting evidence, we agree that these conclusions must be toned down in the manuscript. We also concur that you must extend the discussion to discuss the direct and indirect effects of these observations as explained by the reviewers.
Confirming the efficacy of the siRNA-based experiments would be essential to strengthen this study, using one of the method suggested by reviewers
The reviewers have sought additional details on the results presented in the manuscript that we concur must be provided with text-based additions in the results section (for example Reviewer 1a-b, 4,5, Reviewer 2, points 2, 4, and Reviewer 3, minor points).
Finally we agree with the suggestions of all the reviewers to improve the presentation and clarity of the figures.

In line with their overall assessment, we invite you to submit a revised manuscript addressing the reviewers' comments. When submitting the revision, please include a letter addressing the reviewers' comments point by point. While a rebuttal must respond to all points in some form, additional experiments to resolve these points, other than indicated above, will not be required.

I would be happy to discuss the revision in more detail via email or phone/videoconferencing. Please let me know which option you prefer, if any.

To upload the revised version of your manuscript, please log in to your account: https://lsa.msubmit.net/cgi-bin/main.plex

You will be guided to complete the submission of your revised manuscript and to fill in all necessary information. Please get in touch in case you do not know or remember your login name.

The typical timeframe for revisions is three months. Please note that papers are generally considered through only one revision cycle, so strong support from the referees on the revised version is needed for acceptance.

While you are revising your manuscript, please also attend to the below editorial points to help expedite the publication of your manuscript. Please direct any editorial questions to the journal office.

Thank you for this interesting contribution to Life Science Alliance. We hope that the comments below will prove constructive as your work progresses, and we are looking forward to receiving your revised manuscript.

Sincerely,

Sarita Hebbar, PhD
Scientific Editor
Life Science Alliance
http://www.lsajournal.org

A. THESE ITEMS ARE REQUIRED FOR REVISIONS

-- A letter addressing the reviewers' comments point by point.

-- An editable version of the final text (.DOC or .DOCX) is needed for copyediting (no PDFs).

-- High-resolution figure, supplementary figure and video files uploaded as individual files: See our detailed guidelines for preparing your production-ready images, https://www.life-science-alliance.org/authors

-- Summary blurb (enter in submission system): A short text summarizing in a single sentence the study (max. 200 characters including spaces). This text is used in conjunction with the titles of papers, hence should be informative and complementary to the title and running title. It should describe the context and significance of the findings for a general readership; it should be written in the present tense and refer to the work in the third person. Author names should not be mentioned.

-- By submitting a revision, you attest that you are aware of our payment policies found here: https://www.life-science-alliance.org/copyright-license-fee

B. MANUSCRIPT ORGANIZATION AND FORMATTING:

Full guidelines are available on our Instructions for Authors page, https://www.life-science-alliance.org/authors

We encourage our authors to provide original source data, particularly uncropped/-processed electrophoretic blots and spreadsheets for the main figures of the manuscript. If you would like to add source data, we would welcome one PDF/Excel-file per figure for this information. These files will be linked as supplementary "Source Data" files.

IMPORTANT: It is Life Science Alliance policy that if requested, original data images must be made available. Failure to provide original images upon request will result in unavoidable delays in publication. Please ensure that you have access to all original microscopy and blot data images before submitting your revision.

Reviewer #1 (Comments to the Authors (Required)):

In this study the authors follow up their previous report of L1-derived protein expression in mammalian neurons and ask: what might that protein be doing functionally? They conduct proteomic profiling of the L1-ORF1p-interactome in mouse and human in vitro neurons. They also identify that L1-ORF1p is present in all compartments of these cells, notably including the chromatin-bound fraction. They then follow up these observations in two ways: firstly, they correlate chromatin accessibility changes in neurons with high/low ORF1 expression and in L1-ORF1p-depleted vs control conditions. Secondly they follow up a particular ORF1p-interaction in neurites and report phenotypic changes to the neurites upon knockdown of ORF1p.

The majority of the experiments seem to have been performed well and with appropriate controls, and controls and group sizes are for the most part clearly communicated in the manuscript. I also think it's an interesting area of study: the majority of work in this area have focused on phenotypes that may be driven by L1 RNA and regulatory elements, with a lesser focus on proteins (although it's often hard to distinguish between the two). With that in mind I see clear value in the interactome (Fig1 - assuming the antibodies used are specific). I also like the simple experiment of checking where ORF1 molecules are in the cell (Fig 2) and the idea of thorough characterization of ORF1-low vs ORF1-high neurons (Fig 3). However, the latter figures are (to me at least) a little more problematic. I felt that the ATAC-seq and RNA-seq analysis was a bit overinterpreted given the rather small (very small in case of the L1-KD) and scattered effect sizes observed. In particular, the siRNA-mediated knockdown of L1s has potential off-target effects and confounding factors, and the on-target effects are sparsely characterized - overall I think the data related to the siRNA are therefore the part of the manuscript that could be most improved or toned down. Maybe i've missed something but it's also tricky throughout to define ORF1(protein)-driven vs RNA-driven effects, especially the effects in the nucleus. It's not a dealbreaker but could be good to clearly outline throughout what other effects they could be measuring in any given experiment.

In the end, I would say it's an interesting survey of potential mechanisms of ORF1-dependent phenotypes without nailing any particular one. So i'm left interested by some of the observations but without a really striking take home message. Having said that, I don't think it's a reviewer's job to judge the novelty of a paper (nor do I think an obsession with novelty is a good thing for science...) so I will restrict my comments to analysing the strength of the evidence presented.

In that spirit, some hopefully constructive comments/questions for the authors' consideration:

-I was surprised at the relatively low effect size of the L1 KD and am a bit concerned about the lack of on-target validation and

confounding off target possibilities. I don't think RT-qPCR is a great way to validate the KD (Supp Fig 3B). The Westerns are fine and clear but is there no possibility to analyse the RNA-seq to identify the L1-RNAs being knocked down? Related to this, how many L1s genome wide are (a) transcribed at steady state and (b) targeted by this siRNA sequence? A second siRNA sequence in each species would have helped to control for off-target effects.

-The ATACseq data of L1-high vs L1-low neurons shows some interesting DARs with reasonable effect sizes. Whether that has anything to do with L1s is of course a separate and relevant question, but the description is interesting and worth reporting. It would be logical that some of these DARs were over young L1 promoters - I know there are mappability challenges but did the authors find evidence for this? Sorry if I missed it.

-The overlap between these DARs and the control/KD neurons (where the effect size appears barely measurable even in the snapshot examples) is, however, very low. I applaud the authors for being up-front about this but still, I would encourage the authors to focus mostly on the L1-high/low comparison and characterization and to tone down a lot anything that comes from the siRNA, unless extensive further validation of that treatment is done.

-How are they sure that any effects are protein and not RNA-dependent (cis or trans). Shouldn't the siRNA knock down L1 transcripts also in the nucleus that may drive novel transcripts in cis?

-Are the protein interactors bound in an RNA-dependent manner? Or are they direct? I wonder (maybe for future studies) about screening with AlphaFold for stable complexes.

-For the chromatin interactions, could I clarify: do the authors hypothesize that ORF1p is bound to particular sequences - i.e. is the model that ORF1 is bound with cis-preference to L1-derived RNA that remains chromatin associated in these cells? Outside the scope probably, but it would be cool if they could ChIP the ORF1p (either with the antibody or via the HA tag)? In the absence of such a model it's hard to understand whether and how particular pathways/genes regulated by L1s could be functionally connected? On which point, for the pathway analysis, is the background the genes expressed in the given cell type?

-Some of the figure details are too small and hard to read - particularly genome browser snapshots and interactomes, please check throughout.

I hope my comments are constructive and improve your manuscript.

Christopher Douse
Lund Stem Cell Center

Reviewer #2 (Comments to the Authors (Required)):

Using correlative genomics and loss of function, the authors have addressed the potential role of L1-Orf1 proteins in human neurons. Recent studies have identified the physiological roles of L1s in early embryonic and brain development, primarily focusing on L1 RNA function and its role as a cis-regulatory element in gene regulation. However, as the authors note, the role of L1 proteins is not well understood, except in the context of RT/de novo L1 insertion. The authors observed L1-Orf1 expression in both the cytoplasm and the nucleus of post-mitotic neurons. They explored the potential role of Orf1 proteins. First, they identified Orf1-interacting proteomes using mouse and human dopaminergic cell lines. They found cytoplasmic and nuclear interactors of Orf1, some of which were epigenetic regulators. To infer the potential roles of these interactors, they isolated Orf1-high and Orf1-low NeuN+ cells and compared their chromatin accessibility profiles. No differences in cell type-related accessibility were found between the two populations, but several differentially accessible regions were identified. They noted that these regions exhibited different levels of enrichment of potential binding motifs. However, it is not totally clear whether these changes are driven by Orf1p.
To investigate this, Orf1 was knocked down in human DA neurons using siRNA and differentially accessible regions (DARs) were identified by ATAC-seq. Similar patterns of DAR distribution were observed with Orf1-high/low DARs (enrichment in TSS/Polycomb), but the overlap between the two systems was low (5-10%). Subsequently, they conducted RNA-seq after Orf1 KD and identified differentially expressed genes. They noted that protein-coding long neuronal genes were more preferentially affected. They then overlapped the ATAC-seq and RNA-seq data and found that 26 genes overlapped among the 698 DEGs. While the observation is interesting, there are several major assumptions that are not clearly supported by the data. Therefore, the manuscript requires substantial revision.

The major points are:
1. Although the manuscript concludes that the observed effects are likely derived from Orf1p, no direct evidence is provided. The observed effects could be mediated by LINE-1 RNAs or indirect effects, such as changes in RNA metabolism, translational changes and secondary effects of gene changes. Could the authors verify whether any DARs or DEGs are directly bound by Orf1p? Or could they be rescued by nuclear-targeted Orf1p expression? Otherwise, their major conclusion should be toned down.

2. The authors correlated Orf1-LOF DARs with motifs. Given that they have interactome data, why don't they compare the list of Orf1p interactors with the enriched motifs in Orf1-LOF DARs? Are there any overlaps? If there are any, this would support the idea that Orf1p may be binding to chromatin regions with its partner. Can this be verified by ChIP-qPCR?

(3) siRNA knockdown of LINE-1 could downregulate Orf2 RNA and proteins. How can they be sure that the observed phenotypes are due to Orf1 and not Orf2?

(4)What is the spatial relationship between the expressed LINE-1 elements in the cell lines used and the DAR/DEGs? Can they be sure that the effects of LINE-1 KD are not mediated by nuclear LINE-1 KD? It would be important to check the spatial relationship between the expressed LINE-1 elements in the cell lines/tissue used and the Orf1-associated DARs/DEGs.

Minor point:
In Figure 1E/1F, the names of the proteins are not visible.

Reviewer #3 (Comments to the Authors (Required)):

In this manuscript, Sinnassamy et al. describe a set of observations linking expression of the LINE-1 retrotransposon-encoded ORF1p protein with neuronal gene regulatory patterns and expression, as well as cellular identity and function. A large body of data derives from comparison of neuronal cells expressing low versus high levels of endogenous ORF1p or from cells subjected to siRNA-mediated knockdown of ORF1p. Their observations are intriguing, suggesting a possible physiological role for LINE-1 retrotransposons in neuronal function. They are also timely, with several other recently published articles suggesting interaction of LINE-1 RNA with Polycomb complexes or LINE-1 sequences acting as cis-regulatory elements in embryonic neurogenesis. However, the current manuscript suffers from a lack of controls that renders its conclusions insufficiently robust. The main issues that would need to be resolved before publication are listed below.

Main points:
1. For any siRNA experiment, using at least two independent siRNAs targeting two distinct regions of the target is essential to exclude possible off-target effects. In the current study, only one is used per organism (one for mouse, one for human).
2. It is also good practice to perform rescue experiments. Because the authors target repeated sequences that can also be dispersed in genes, knocking down LINE-1 may also affect genes that contain them (not strictly speaking off-target effects, but rather collateral effects). This may explain why the comparison of ORF1p high/low versus ORF1p control/knockdown yields minimal overlap and why longer genes (which have a higher probability of containing L1 sequences) are more affected by LINE-1 knockdown.
3. Whether some of the observed effects are direct and whether they are mediated by ORF1p or LINE-1 RNA requires clarification. Therefore, the rescue could be performed with LINE-1 sequences that can or cannot be translated into ORF1p.

Minor points:
4. It would be helpful to clarify whether, in this experimental system, ORF1p binding to chromatin is RNA-dependent and whether ORF1p can directly bind DNA (ChIP-seq).
5. Subcellular fractionation experiments would benefit from quantification. Some fractions are missing in Fig. S3C.
6. Do differentially expressed genes upon LINE-1 knockdown contain L1 sequences and/or potential binding sites for the LINE-1 siRNA?
7. When performing knockdown of LINE-1, there is a minimal effect on LINE-1 RNA by RT-qPCR (Fig. S3B). What about separating cytoplasmic versus nuclear RNA? It would be helpful to understand which fraction of LINE-1 RNA is actually affected by the knockdown.
8. Fig. S3 B and C: legends were inverted.
9. Fig. S3D does not show DAR−/DAR+ unlike the main text description (lines 360-364).
10. Fig. 4E/F: track legends (on the left) do not correspond to the displayed tracks.
11. Referencing: in the introduction, the original primary papers showing LINE-1 expression in the brain are missing (from the Gage and Faulkner labs). Most cited preprints are now published (Zernia et al., Sci. Adv. 2025; Adami et al., Cell Genom. 2025; Bonnifet et al., eLife 2025; Zhang et al., Dev. Cell 2025) and could be referenced as such.
12. The article by Della Valle et al., Sci. Transl. Med. 2022, which describes the role of LINE-1 RNA in heterochromatin formation, notably through SUV39H1 regulation, is directly related to the present study and would merit citation and discussion.
13. Although this could be due to the submission system, the quality of network graphs could be improved, as they are not readily readable in their present state.
14. Comparison of IP/MS between mouse and human, and with previous ORF1p IP/MS obtained in other systems, could be enhanced in the supplementary table: which interactor was found in which study/sample? (Adding a column per cell type and/or study, rather than a simple list of "known interactors," would facilitate comparison).

Dear editors,

We thank all the reviewers for their constructive feed-back and evaluation of our manuscript and reply below, in a point-by-point manner, to their comments. Our responses are in blue.

Reviewer #1 (Comments to the Authors (Required)):

In this study the authors follow up their previous report of L1-derived protein expression in mammalian neurons and ask: what might that protein be doing functionally? They conduct proteomic profiling of the L1-ORF1p-interactome in mouse and human in vitro neurons. They also identify that L1-ORF1p is present in all compartments of these cells, notably including the chromatin-bound fraction. They then follow up these observations in two ways: firstly, they correlate chromatin accessibility changes in neurons with high/low ORF1 expression and in L1-ORF1p-depleted vs control conditions. Secondly they follow up a particular ORF1p-interaction in neurites and report phenotypic changes to the neurites upon knockdown of ORF1p.

The majority of the experiments seem to have been performed well and with appropriate controls, and controls and group sizes are for the most part clearly communicated in the manuscript. I also think it's an interesting area of study: the majority of work in this area have focused on phenotypes that may be driven by L1 RNA and regulatory elements, with a lesser focus on proteins (although it's often hard to distinguish between the two). With that in mind I see clear value in the interactome (Fig1 - assuming the antibodies used are specific). I also like the simple experiment of checking where ORF1 molecules are in the cell (Fig 2) and the idea of thorough characterization of ORF1-low vs ORF1-high neurons (Fig 3). However, the latter figures are (to me at least) a little more problematic. I felt that the ATAC-seq and RNA-seq analysis was a bit overinterpreted given the rather small (very small in case of the L1-KD) and scattered effect sizes observed. In particular, the siRNA-mediated knockdown of L1s has potential off-target effects and confounding factors, and the on-target effects are sparsely characterized - overall I think the data related to the siRNA are therefore the part of the manuscript that could be most improved or toned down. Maybe i've missed something but it's also tricky throughout to define ORF1(protein)-driven vs RNA-driven effects, especially the effects in the nucleus. It's not a dealbreaker but could be good to clearly outline throughout what other effects they could be measuring in any given experiment.

In the end, I would say it's an interesting survey of potential mechanisms of ORF1-dependent phenotypes without nailing any particular one. So i'm left interested by some of the observations but without a really striking take home message. Having said that, I don't think it's a reviewer's job to judge the novelty of a paper (nor do I think an obsession with novelty is a good thing for science...) so I will restrict my comments to analyzing the strength of the evidence presented.

In that spirit, some hopefully constructive comments/questions for the authors' consideration:

-I was surprised at the relatively low effect size of the L1 KD and am a bit concerned about the lack of on-target validation and confounding off target possibilities. I don't think RT-qPCR is a great way to validate the KD (Supp Fig 3B). The Westerns are fine and clear but is there no possibility to analyze the RNA-seq to identify the L1-RNAs being knocked down? Related to this, how many L1s genome wide are (a) transcribed at steady state and (b) targeted by this siRNA sequence? A second siRNA sequence in each species would have helped to control for off-target effects.

On-target validation

We validated on-target effects by Western blot and RTqPCR showing efficient protein and (less efficient, non-significant) RNA knock-down. As requested by the reviewer, we have now added new data based on the RNA-seq experiment. We reanalyzed the RNA-seq data with a TE-adapted mapping method (for details please see Materials & Methods) normalized the raw counts by gene +TE counts and plotted the normalized expression of young LNE-1 families in a heatmap, now added to Suppl. Fig. 3B (and copied below). As expected, the youngest LINE-1 families L1HS and L1PA2 are less transcribed than the older families L1PA3-5. On the right is a depiction of the data for L1HS and L1PA2 separately, in dot plot format (mean+/-sem, n=4)). Overall, the siRNA did not downregulate LINE-1 RNA at the family level, but most probably certain L1HS/L1PA2 elements more specifically, which is why we observe a siRNA-dependent trend for downregulation when using LINE-1 primers located in the 5'UTR of young LINE-1 elements (as shown in Suppl Fig 3B) and a significant downregulation of LINE-1 in RTqPCR upon siRNA-mediated KD of LINE-1 using a second siRNA against LINE-1 (please see new Suppl Fig 5).

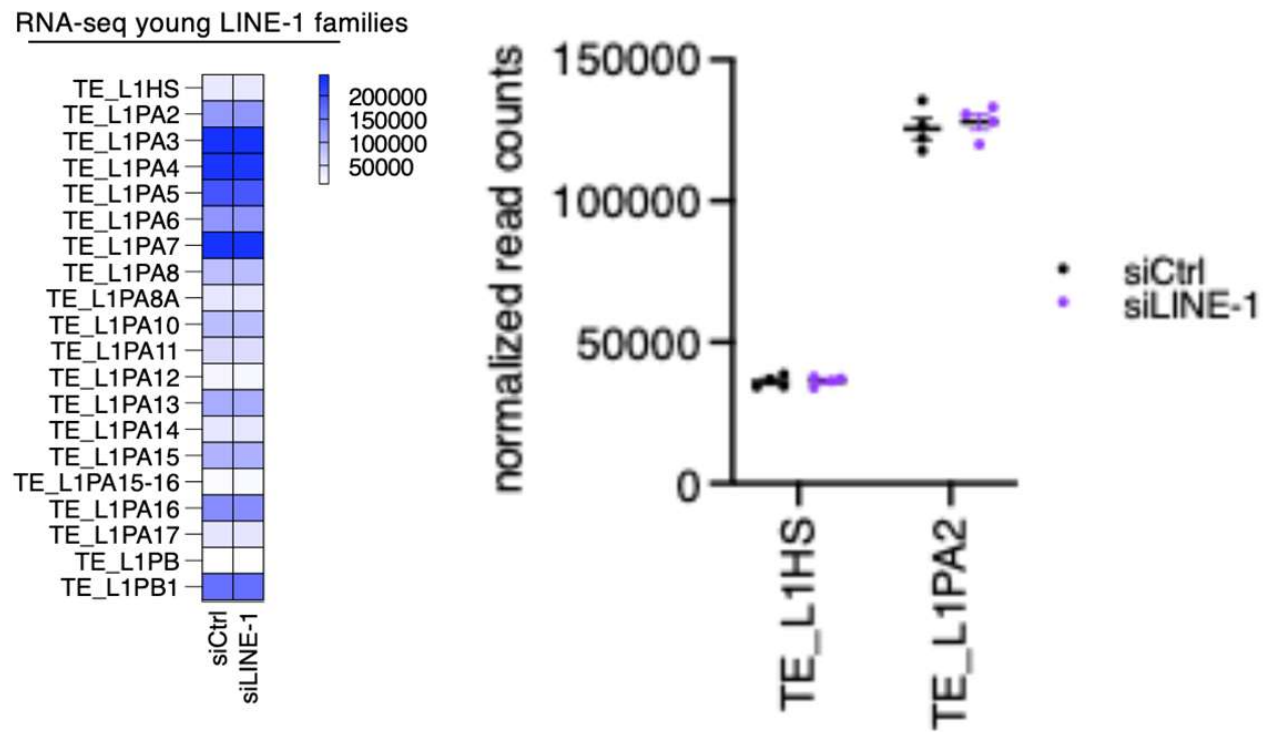

We added the following sentence to the manuscript, line 337: » Efficient knock-down of ORF1p was verified by immunostaining with a significant reduction in the cytoplasmic and the nuclear compartment (Fig. 4A) and by Western blot (Suppl. Fig. 3A) while LINE-1 RNA was slightly but not significantly reduced in independent RTqPCR experiments (Suppl. Fig. 3B, left) and not reduced at the family-level of the youngest LINE1 families in the RNA-seq data as depicted in the heatmap in Suppl. Fig. 3B, right)."

<u>Potential off-target effects/ use of a second siRNA</u>
Initially, we had tried other siRNA sequences in our system and unfortunately had not identified any other siRNA sequence with efficient silencing of ORF1p expression (evaluated by Western blot). However, we had always used a slightly different timing of analysis after transfection, that is, we harvested cells on day 5 (transfection was done on day 3 for the neurons to be differentiated), 48h after transfection. We then changed to a harvest at day 7 (96h after transfection) and this is when we reproducibly observed a downregulation of the ORF1 protein. Most probably, the important difference was the timing of harvest due to a high stability of the ORF1 protein. In this revision, we have now successfully tested one other siRNA sequence (siLINE-1 #2) which downregulated ORF1p and LINE-1 RNA. In order to validate whether the effect on the downregulation of specific genes was reproducible when using this independent siRNAs against LINE1, we quantified the expression of the same RNAs identified as downregulated in the RNA-seq and independently validated in RTqPCR using the first siRNA as in Figure 6G, now using siLINE-1 #2. The results are now added as Suppl Fig 5A-D.
We added the following data to the manuscript (<u>Suppl Figure 5):</u>

Suppl Figure 5

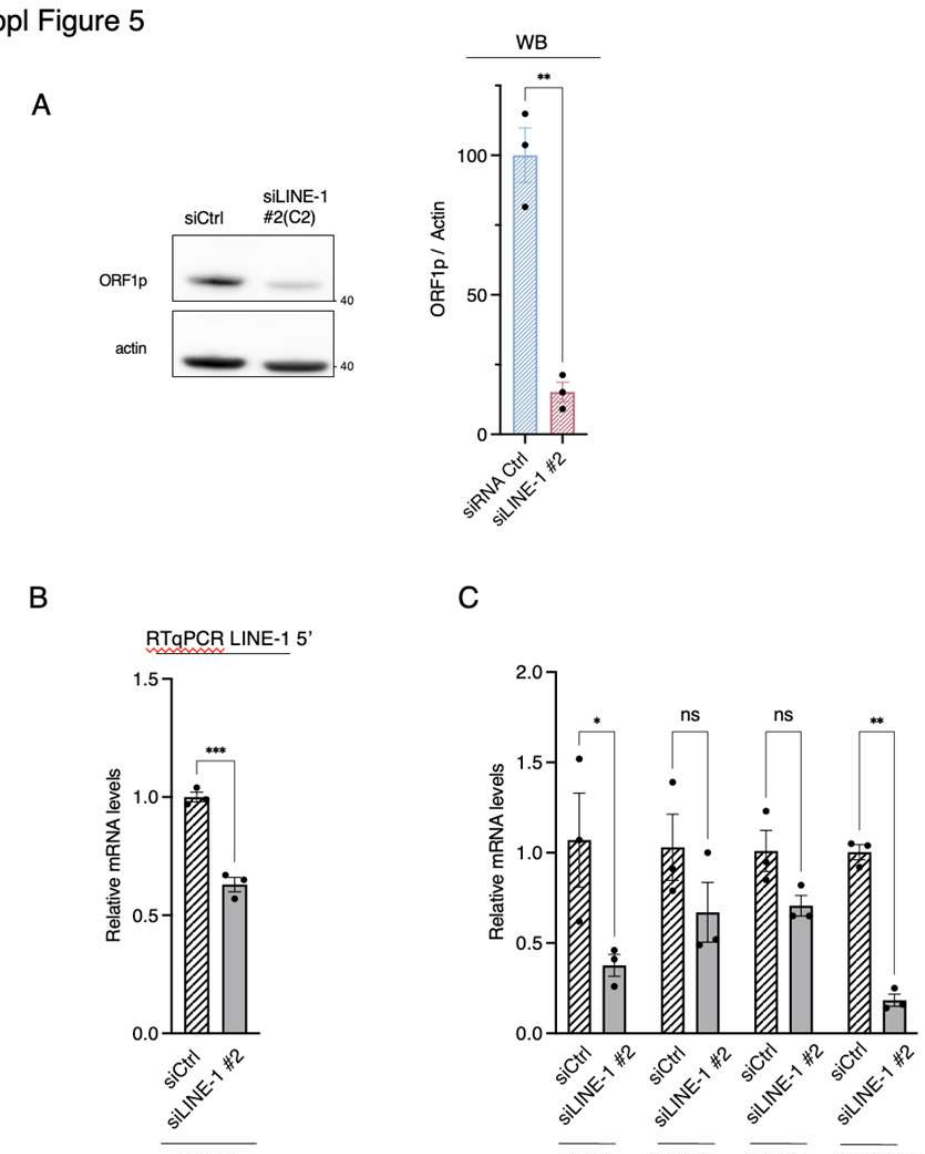

Line 467: "<u>In order to independently validate the LINE-1 KD mediated downregulation of neuron-related genes, we used an additional s targeting LINE-1. Despite difficulties in selecting siRNAs knocking down LINE-1 ORF1p in LUHMES neurons, where the ORF1p protein seems to be particularly stable, we identified another LINE-1 siRNA</u>

which knocked down ORF1p efficiently as shown and quantified in Suppl. Fig. 5A using the same protocol as above. Using this LINE-1 siRNA ((siLINE-1 #2), LINE-1 RNA was significantly downregulated (Suppl. Fig. 5B). We then tested this siRNA targeting LINE-1 in RTqPCR selecting the same four genes we had identified as downregulated in the RNA-seq experiment and in RTqPCR using siLINE-1 #1 as shown above (Fig.6). We confirmed the downregulation of DLRG5 and GABRB3, and there was a trend, but no significant downregulation, of the expression of NEGR1 and EFNA5 (Suppl. Fig. 5C)."

Concerning potential on and off-targets, we also did additional *in silico* analyses of the human and mouse siRNA used.

Human siRNA:

The human LINE1-ORF1 siRNA sequences we used had been evaluated by another group in a previous publication (Philippe et al, eLife, 2016). We evaluated the two sequences in this study (siLINE-1 and siLINE-1 #2), which the authors had successfully used as shRNA for LINE-1 KD, The first sequence which efficiently knocked-down ORF1p in our experimental system was (AAGAAGGCTTCAGACGATCAA which was adapted from the shRNA#1 used in Philippe et al, eLife, 2016 -> CCGG**AAGAAGGCTTCAGACGATCAA**CTCGAGTTGATCGTCTGAAGCCTTCTTTTTTTG with the target sequence indicated in bold). They showed that this shRNA efficiently knocked-down ORF1p (WB) and LINE-1 RNA (RNA-seq) in two different cell lines, namely MCF7 and 2102Ep (Philippe et al, eLife, 2016 -> Figure 3C, D and E). Using ClustalW alignment of all 146 full-length LINE-1 with intact open reading frames ORF1 and ORF2 identified in the LINE-1Base version 2 database (Penzkofer et al, NAR, 2017), we identified the target sequence in 134 of the 146 full-length LINE-1 sequences with intact coding potential.

On and Off-target assessment of the LINE-1 siRNA was performed *in silico* using BLASTn-short against the human genome and transcriptome. ORF1 was identified, as expected, confirming LINE-1 as the on-target. We agree that siRNA off-target effects are an important consideration. Three protein-coding transcripts (GRHL2, ZNF66 and SOD2) exhibited extended complementarity (as defined by 100% coverage and >95% complementarity) in this BLAST analysis, indicating the possibility of Ago2-mediated cleavage. We therefore analyzed the RNA-seq data in our cellular system after LINE-1 KD as to whether the expression of these three potential targets was effected. All three transcripts were not significantly regulated comparing siRNA ORF1p to scrambled siRNA with raw and adjusted p-values >0.05 and visual inspection of all loci confirmed this throughout the whole gene locus. BLAST identified nine non-coding RNAs, of which all, but one (PENK-AS1) contained a L1 PA2, PA3, PA4 or PA5 element. None of these transcripts was significantly regulated upon LINE-1 KD.

Mouse siRNA : The mouse siRNA stems from a previous publication from our group (Blaudin de Thé et al, EMBO J, 2018) where we could show, importantly along two other anti-LINE-1 strategies, that the infusion of this siRNA (coupled to penetratin) was sufficient to rescue mouse dopaminergic cell loss *in vivo* and to prevent DNA strand breaks in primary neurons *in vitro*. We have corrected in the manuscript, that this sequence is directed against ORF2 and not ORF1, our apologies for this error. Blastn optimized for short sequences did not reveal any off targets matching 100%, but did reveal one potential off-target, namely Maip1, which is a nuclear encoded mitochondrial protein involved in calcium transport (https://www.genecards.org/cgi-bin/carddisp.pl?gene=MAIP1).

-The ATACseq data of L1-high vs L1-low neurons shows some interesting DARs with reasonable effect sizes. Whether that has anything to do with L1s is of course a separate and relevant question, but the description is interesting and worth reporting. It would be logical that some of these DARs were over young L1 promoters - I know there are mappability challenges but did the authors find evidence for this? Sorry if I missed it.

We overlapped the genomic intervals of all DARs identified in both, human post-mortem nuclei (ORF1p-high/low) and LUHMES neurons (Ctrl/LINE1-KD) over the young LINE1 families L1HS and L1PA2. We found one overlap (IGV screenshot shown below) of a DAR+ peak in ORF1p-low neurons with a L1PA2 (not full-length and coding). Otherwise, there were no overlaps between genomic regions with accessibility changes depending on LINE1/ORF1p content, indicating that LINE1 RNA and/or encoding proteins do not interfere with the regulation of the chromatin accessibility of young LINE1 sequences.

We added the following sentence to the manuscript

Line 280 : « To test whether DARs were present on young LINE1 elements, we overlapped the genomic intervals of all DARs and L1HS and L1PA2 elements and found one single overlap (chr3:195087505-195087829, intronic L1PA2 within the XXYLT1 gene), suggesting that LINE1 RNA and/or encoding proteins do not interfere with the regulation of the chromatin accessibility of young LINE1 sequences. »

Line 376 : « To test whether DARs were present on young LINE-1 elements, we overlapped the genomic intervals of all DARs and L1HS and L1PA2 elements and found no overlap, again suggesting that LINE-1 RNA and/or encoding proteins do not interfere with the regulation of the chromatin accessibility of young LINE-1 sequences. "

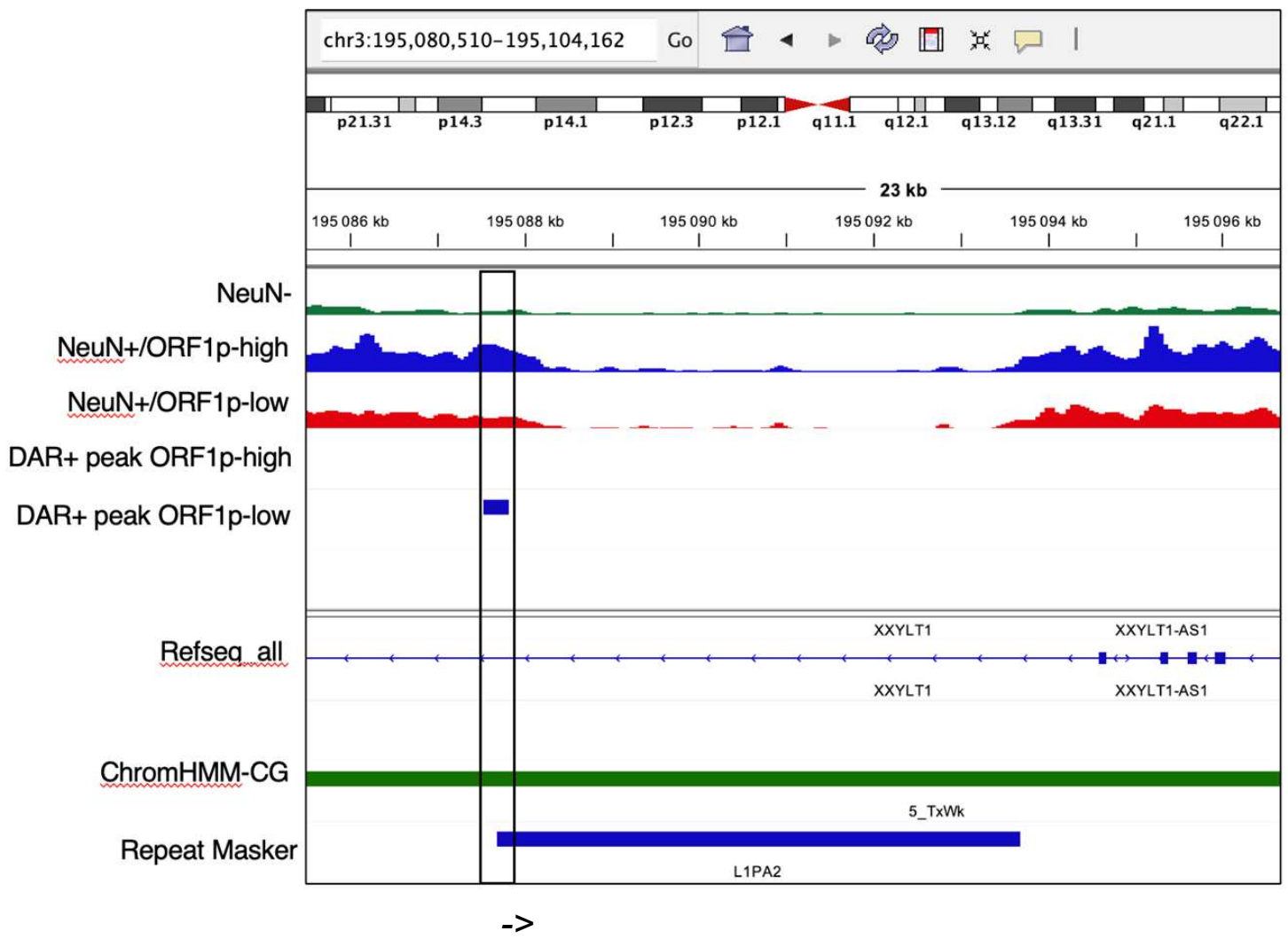

-The overlap between these DARs and the control/KD neurons (where the effect size appears barely measurable even in the snapshot examples) is, however, very low. I applaud the authors for being up-front about this but still, I would encourage the authors to focus mostly on the L1-high/low comparison and characterization and to tone down a lot anything that comes from the siRNA, unless extensive further validation of that treatment is done.

We agree. However, it is important to notice that while overall there weren't many overlaps in DARs between ORF1p-high/low and siRNA control and LINE1-KD nuclei, we made overall similar observations; nuclei containing high levels of ORF1p showed more accessible chromatin preferentially in distal intergenic regions associated with polycomb-associated repressive domains and nuclei with low ORF1p content were predominantly characterized by more accessible DARs in intronic regions associated with enhancer functions and active transcription containing signatures of homeobox transcription factor binding sites (please see also Table 1). This gives some confidence on similar changes operating on chromatin depending on the nuclear ORF1p content.
We have now also added new data validating the siRNA-mediated effect using a second siRNA LINE-1 (siRNA LINE-1 #2) followed by RTqPCR of RNA-seq identified genes downregulated with the siRNA#1 (see Fig. 6G), shown in new Suppl. Fig 5. Please also see above.

-How are they sure that any effects are protein and not RNA-dependent (cis or trans). Shouldn't the siRNA knock down L1 transcripts also in the nucleus that may drive novel transcripts in cis?

Indeed, we cannot be sure that the effects are solely protein and not RNA-dependent. Indeed, LINE-1 RNA revealed by RNAscope seems detectable only in the nucleus in differentiated LUHMEs neurons (preliminary data). However, both in the RNA-seq data as well as in independent RTqPCR experiments, LINE1 RNA content was only variably affected by the LINE-1 siRNA. This indeed suggests that what we observe is at least not majorly driven by L1 RNA-dependent changes and reinforces the notion of ORF1 protein-dependent mechanisms especially when taking into account the detectable decrease of ORF1p on chromatin after LINE1-KD (Suppl. Fig. 3C).
In the case of human brain nuclei containing high or low ORF1p, we do not know whether ORF1p is bound or not to LINE-1 RNA. Importantly, whether we characterize accessible chromatin features in human brain or in human neurons *in vitro*, we do observe similar overall characteristics depending on the content of ORF1p in the nucleus/cells (please see also table 1). This does suggest that in both systems, the fact of having high or low ORF1p content, is associated with similar chromatin accessibility changes and indicates at least that these effects are protein-dependent and may or may not be RNA-dependent.

-Are the protein interactors bound in an RNA-dependent manner? Or are they direct? I wonder (maybe for future studies) about screening with AlphaFold for stable complexes.

Indeed, a very interesting question, but we did not include a +/-RNAseA condition. This would indeed be very interesting and important to address in future studies.

-For the chromatin interactions, could I clarify: do the authors hypothesize that ORF1p is bound to particular sequences - i.e. is the model that ORF1 is bound with cis-preference to L1-derived RNA that remains chromatin associated in these cells? Outside the scope probably, but it would be cool if they could ChIP the ORF1p (either

with the antibody or via the HA tag)? In the absence of such a model it's hard to understand whether and how particular pathways/genes regulated by L1s could be functionally connected? On which point, for the pathway analysis, is the background the genes expressed in the given cell type?

This question how ORF1p effects chromatin and how it binds to chromatin is extremely interesting, We do not have a model for this as our attempts to ChIP ORF1p were not successful, but we discuss possibilities in the discussion:
Line 642: » How exactly nuclear ORF1p can regulate chromatin accessibility remains to be investigated. One possibility is through the association with LINE-1 RNA or directly with proteins involved in chromatin-related functions. Indeed, we identified many nuclear proteins with chromatin-related function interacting with ORF1p. These protein interactors of ORF1p provide an extensive network of proteins related to chromatin organization and transcription regulation. Three protein interactors of ORF1p stood out as they were identified as common ORF1p interacting proteins in both, mouse and human neurons: RCOR2 (corepressor 2 of REST), a transcriptional repressor required for mouse ESC differentiation[84] and neurogenesis[85,86], YTHDC1, a protein implicated in m6A-dependent RNA metabolism[87] and HNRNPU, a DNA and RNA binding protein associated with various neurodevelopmental disorders[88]. HNRNPU is involved in chromatin organization, transcription regulation and many other nuclear processes[89] and has been previously identified as an ORF1p interactor in cancerous cell lines[33]. Interestingly, YTHDC1 has been shown to bind m6A-modified LINE-1 RNA in order to form a protein-RNA complex which represses the 2-cell program in ESCs[90] but might also be involved in neuronal survival[91] and cerebral stress response[92]. It would be interesting to test the functional relevance of the interaction of ORF1p with these proteins in mediating chromatin accessibility in neurons. While the functional impact of these protein-protein interactions remains to be determined, their identification reinforces the hypothesis that it might be ORF1p that modulates chromatin accessibility in mature neurons, either directly or indirectly through the binding to these proteins and/or to RNA. Given the extensive list of ORF1p protein interactors with functions in RNA metabolism, we cannot exclude that ORF1p might also regulate gene expression post-transcriptionally. While many of these interactions were consistent with previously identified protein partners of ORF1p in human cancer cell lines, mouse brain tissues and sperm and were identified in well-established neuronal cell lines, future studies in primary neurons or brain tissue will be important to confirm their relevance *in vivo* and fully characterize the specificity and strength of these associations. »
Experimentally, we tried both, to ChIP endogenous ORF1p using the ORF1p antibody and to ChIP HA-tagged ORF1p after transfection with an HA antibody (low nucleofection yield), but did not get clean, trustworthy results. Indeed, a ChIP-ORF1p +/-RNAse would have allowed to address this urgent question.

-Some of the figure details are too small and hard to read - particularly genome browser snapshots and interactomes, please check throughout.

We improved the visual aspect of the genome browser snapshots (please see Figure 3G,H ; Figure 4E,F) or took them out completely (Figure 5). We could not, however, improve the interactomes and added instead a new table (Suppl Table 2) which contains all the information of proteins belonging to each GO category. We added (line 185: « please also see Suppl Table 2 for further information on proteins belonging to each category")

I hope my comments are constructive and improve your manuscript.

Thank you very much for your comments which were indeed very constructive and, we believe (and hope you agree), have improved our manuscript!

Reviewer #2 (Comments to the Authors (Required)):

Using correlative genomics and loss of function, the authors have addressed the potential role of L1-Orf1 proteins in human neurons. Recent studies have identified the physiological roles of L1s in early embryonic and brain development, primarily focusing on L1 RNA function and its role as a cis-regulatory element in gene regulation. However, as the authors note, the role of L1 proteins is not well understood, except in the context of RT/de novo L1 insertion. The authors observed L1-Orf1 expression in both the cytoplasm and the nucleus of post-mitotic neurons. They explored the potential role of Orf1 proteins. First, they identified Orf1-interacting proteomes using mouse and human dopaminergic cell lines. They found cytoplasmic and nuclear interactors of Orf1, some of which were epigenetic regulators. To infer the potential roles of these interactors, they isolated Orf1-high and Orf1-low NeuN+ cells and compared their chromatin accessibility profiles. No differences in cell type-related accessibility were found between the two populations, but several differentially accessible regions were identified. They noted that these regions exhibited different levels of enrichment of potential binding motifs. However, it is not totally clear whether these changes are driven by Orf1p.
To investigate this, Orf1 was knocked down in human DA neurons using siRNA and differentially accessible regions (DARs) were identified by ATAC-seq. Similar patterns of DAR distribution were observed with Orf1-high/low DARs (enrichment in TSS/Polycomb), but the overlap between the two systems was low (5-10%). Subsequently, they conducted RNA-seq after Orf1 KD and identified differentially expressed genes. They noted that protein-coding long neuronal genes were more preferentially affected. They then overlapped the ATAC-seq and RNA-seq data and found that 26 genes overlapped among the 698 DEGs.

While the observation is interesting, there are several major assumptions that are not clearly supported by the data. Therefore, the manuscript requires substantial revision.

The major points are:
1. Although the manuscript concludes that the observed effects are likely derived from Orf1p, no direct evidence is provided. The observed effects could be mediated by LINE-1 RNAs or indirect effects, such as changes in RNA metabolism, translational changes and secondary effects of gene changes. Could the authors verify whether any DARs or DEGs are directly bound by Orf1p? Or could they be rescued by nuclear-targeted Orf1p expression? Otherwise, their major conclusion should be toned down.

We agree that the observed effects could be mediated by ORF1p and LINE-1 RNA in conjunction and we discuss this possibility in the discussion (line 608-641). However, we think that indirect effects as the only functional mechanisms are not likely due to the overlap of many functional features detected in the ATAC-seq in both, the human brain tissue and neuronal cells in culture (see Table 1), but indeed cannot be ruled out completely. We agree, that it would have been important to have a ChIP ORF1p in order to prove and map direct DNA interactions and to characterize them. Unfortunately, our attempts to ChIP ORF1p (using either the ORF1p antibody or, after transfection of an HA-tagged ORF1p with the HA antibody – low nucleofection yield) did not generate clean, trustworthy results. Indeed, a ChIP-ORF1p +/-RNAse would have allowed to address this urgent question.

To tone down, we have replaced ORF1p-LOF by LINE-1 KD. We have also made other changes throughout the manuscript:
Line 699: « Although we have provided correlative genomic and loss of function evidence for an involvement of ORF1p in adult neuronal physiology, our study has some limitations. We cannot definitively exclude that the observed effects are partially mediated by LINE-1 RNA or other indirect effects or whether ORF1p is bound to LINE-1 RNA on chromatin. The identification of ORF1p chromatin binding sites, the characterization of the ORF1p complex binding chromatin and/or rescue experiments of ORF1p should be the subject of future studies."
Line 349: "Although effect sizes were rather low, we observed…."
Line 622: "Whether once in the nucleus, ORF1p associates with RNA or not to bind chromatin, is an open question, which remains to be addressed."
Line 640: "Whether LINE-1 RNA might be involved or not remains to be investigated. »
Line 643:" One possibility is through the association with LINE-1 RNA or directly with proteins involved in chromatin-related functions."
Line 502: "LINE-1 knock-down leads to changes in neurites morphology"
"In addition to the fact that knock-down of LINE-1 induced chromatin accessibility and transcriptomic changes which suggested that LINE-1 ORF1p (+/-LINE-1 RNA) might be involved in regulating the homeostasis and maintenance of adult neurons at steady-state."
Line 545:" In summary, these data suggest that LINE-1 ORF1p, in conjunction or not with LINE-1 RNA, plays a role in the maintenance of neurite morphology possibly by converging pathways mediated by protein interactions and by modulating chromatin accessibility and transcriptomic landscapes."

2. The authors correlated Orf1-LOF DARs with motifs. Given that they have interactome data, why don't they compare the list of Orf1p interactors with the enriched motifs in Orf1-LOF DARs? Are there any overlaps? If there are any, this would support the idea that Orf1p may be binding to chromatin regions with its partner. Can this be verified by ChIP-qPCR?

We compared the transcription factors predicted to bind to chromatin with accessibility changes in high compared to low nuclear ORF1p nuclei or control compared to LINE-1 KD, but did not find an overlap with ORF1p interacting proteins. Nevertheless, among ORF1p interacting proteins were many proteins involved in gene expression in both, mouse and human neurons. These are now listed in Suppl Table 2. In MN9D, there were 37 ORF1p protein interactors belonging to GO category « negative gene regulation » and 4 RNA polymerase II core complex proteins. In LUHMES, there were 99 proteins belonging to the GO category « Gene expression ». We discuss this in the discussion line 642 : « How exactly nuclear ORF1p can regulate chromatin accessibility remains to be investigated. One possibility is through the association with LINE-1 RNA or directly with proteins involved in chromatin-related functions. Indeed, we identified many nuclear proteins with chromatin-related function interacting with ORF1p. These protein interactors of ORF1p provide an extensive network of proteins related to chromatin organization and transcription regulation. Three protein interactors of ORF1p stood out as they were identified as common ORF1p interacting proteins in both, mouse and human neurons: RCOR2 (corepressor 2 of REST), a transcriptional repressor required for mouse ESC differentiation[84] and neurogenesis[85,86], YTHDC1, a protein implicated in m6A-dependent RNA metabolism[87] and HNRNPU, a DNA and RNA binding protein associated with various neurodevelopmental disorders[88]. HNRNPU is involved in chromatin organization, transcription regulation and many other nuclear processes[89] and has been previously identified as an ORF1p interactor in cancerous cell lines[33]. Interestingly, YTHDC1 has been shown to bind m6A-modified LINE-1 RNA in order to form a protein-RNA complex which represses the 2-cell program in ESCs[90] but might also be involved in neuronal survival[91] and cerebral stress response[92]. It would be interesting to test the functional relevance of the interaction of ORF1p with these proteins in mediating chromatin accessibility in

neurons. While the functional impact of these protein-protein interactions remains to be determined, their identification reinforces the hypothesis that it might be ORF1p that modulates chromatin accessibility in mature neurons, either directly or indirectly through the binding to these proteins and/or to RNA. Given the extensive list of ORF1p protein interactors with functions in RNA metabolism, we cannot exclude that ORF1p might also regulate gene expression post-transcriptionally. While many of these interactions were consistent with previolsy identified protein partners of ORF1p in human cancer cell lines, mouse brain tissues and sperm and were identified in well-established neuronal cell lines, future studies in primary neurons or brain tissue will be important to confirm their relevance *in vivo* and fully characterize the specificity and strength of these associations.

(3) siRNA knockdown of LINE-1 could downregulate Orf2 RNA and proteins. How can they be sure that the observed phenotypes are due to Orf1 and not Orf2?

Indeed, we cannot rule out that ORF2p is affected by the siRNA directed against a sequence in the ORF1 region as we are dealing with a polycistronic RNA. We have, however, not detected the expression of ORF2p by Western blot, by immunofluorescence or by mass spectrometry in the cells used in this study. Importantly, it is generally now accepted (Nielsen et al, Mobile DNA, 2025), that ORF2p is not expressed in healthy tissues, and thus is most probably not expressed in human neurons or neuronal cell lines at steady state as used in this present study.

(4) What is the spatial relationship between the expressed LINE-1 elements in the cell lines used and the DAR/DEGs? Can they be sure that the effects of LINE-1 KD are not mediated by nuclear LINE-1 KD? It would be important to check the spatial relationship between the expressed LINE-1 elements in the cell lines/tissue used and the Orf1-associated DARs/DEGs.

Yes, the reviewer is right, we cannot completely rule out that there is a nuclear LINE-1 KD although the RTqPCR and RNA-seq quantifications of young L1HS and L1PA2 expression do not show a significant LINE-1 RNA knock-down with siRNA#1 or a slight KD with siRNA#2. Very preliminary experiments using LINE-1 RNAscope (in LUHMES) suggest that LINE-1 RNA is almost exclusively nuclear. However, we know that ORF1p is nuclear and that the accessibility differences in human brain nuclei are associated with ORF1p nuclear content. We overlapped the genomic intervals of all DARs identified in both, human post-mortem nuclei (ORF1p-high/low) and LUHMES neurons (Ctrl/LINE1-KD) over the young LINE1 families L1HS and L1PA2. We found one overlap (IGV screenshot shown below) of a DAR+ peak in ORF1p-low (ATAC-seq human) neurons with a L1PA2 (not full-length and coding). In LUHMES neurons there was no overlaps between genomic regions with accessibility changes depending on LINE1/ORF1p content (ATAC-seq DAR overlap with L1HS and L1PA2 elements), indicating that LINE1 RNA and/or encoding proteins do not interfere with the regulation of the chromatin accessibility of young LINE1 sequences.
We added the following sentences to the manuscript (regarding ATAC-seq human tissues:
Line 280: « To test whether DARs were present on young LINE-1 elements, we overlapped the genomic intervals of all DARs and L1HS and L1PA2 elements and found one single overlap (chr3:195087505-195087829, intronic L1PA2 within the XXYLT1 gene), suggesting that LINE-1 RNA and/or encoding proteins do not interfere with the regulation of the chromatin accessibility of young LINE-1 sequences. «
Line 376: « To test whether DARs were present on young LINE-1 elements, we overlapped the genomic intervals of all DARs and L1HS and L1PA2 elements and found no overlap, again suggesting that LINE-1 RNA and/or encoding proteins do not interfere with the regulation of the chromatin accessibility of young LINE-1 sequences. »

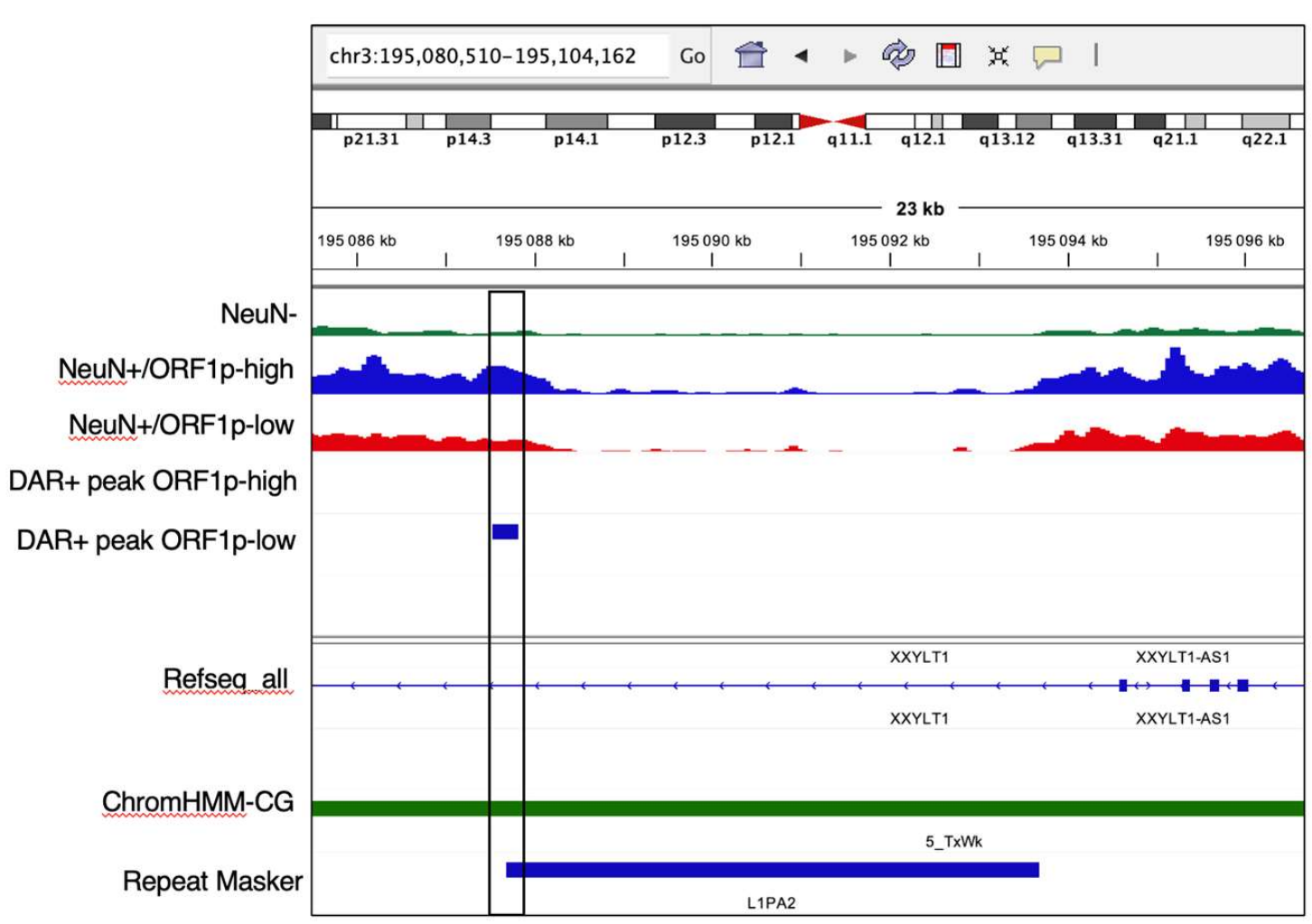

->

Minor point:
In Figure 1E/1F, the names of the proteins are not visible.

We added a new table (Suppl Table 2) which contains all the information of proteins belonging to each GO category as depicted in Figure 1E/F.

We thank reviewer #2 for her/his comments and suggestions.

Reviewer #3 (Comments to the Authors (Required)):

In this manuscript, Sinnassamy et al. describe a set of observations linking expression of the LINE-1 retrotransposon-encoded ORF1p protein with neuronal gene regulatory patterns and expression, as well as cellular identity and function. A large body of data derives from comparison of neuronal cells expressing low versus high levels of endogenous ORF1p or from cells subjected to siRNA-mediated knockdown of ORF1p. Their observations are intriguing, suggesting a possible physiological role for LINE-1 retrotransposons in neuronal function. They are also timely, with several other recently published articles suggesting interaction of LINE-1 RNA with Polycomb complexes or LINE-1 sequences acting as cis-regulatory elements in embryonic neurogenesis. However, the current manuscript suffers from a lack of controls that renders its conclusions insufficiently robust. The main issues that would need to be resolved before publication are listed below.

Main points:
1. For any siRNA experiment, using at least two independent siRNAs targeting two distinct regions of the target is essential to exclude possible off-target effects. In the current study, only one is used per organism (one for mouse, one for human).

Potential off-target effects/ use of a second siRNA
Initially, we had tried other siRNA sequences in our system and unfortunately had not identified any other siRNA sequence with efficient silencing of ORF1p expression (evaluated by Western blot). However, we had always used a slighly different timing of analysis after transfection, that is, we harvested cells on day 5 (transfection was done on day 3 for the neurons to be differentiated), 48h after transfection. We then changed to a harvest at day 7 (96h after transfection) and this is when we reproducibly observed a downregulation of the ORF1 protein. Most probably, the important difference was the timing of harvest due to a high stability of the ORF1 protein. In this revision, we have now successfully tested one other siRNA sequence (siLINE-1 #2) which downregulated ORF1p and LINE-1 RNA. In order to validate whether the effect on the downregulation of specific genes was reproducible when using this independent siRNAs against LINE1, we quantified the expression of the same RNAs identified as downregulated in the RNA-seq and independently validated in RTqPCR using the first siRNA as in Figure 6G, now using siLINE-1 #2. The results are now added as Suppl Fig 5A-D.
We added the following data to the manuscript (Suppl Figure 5):

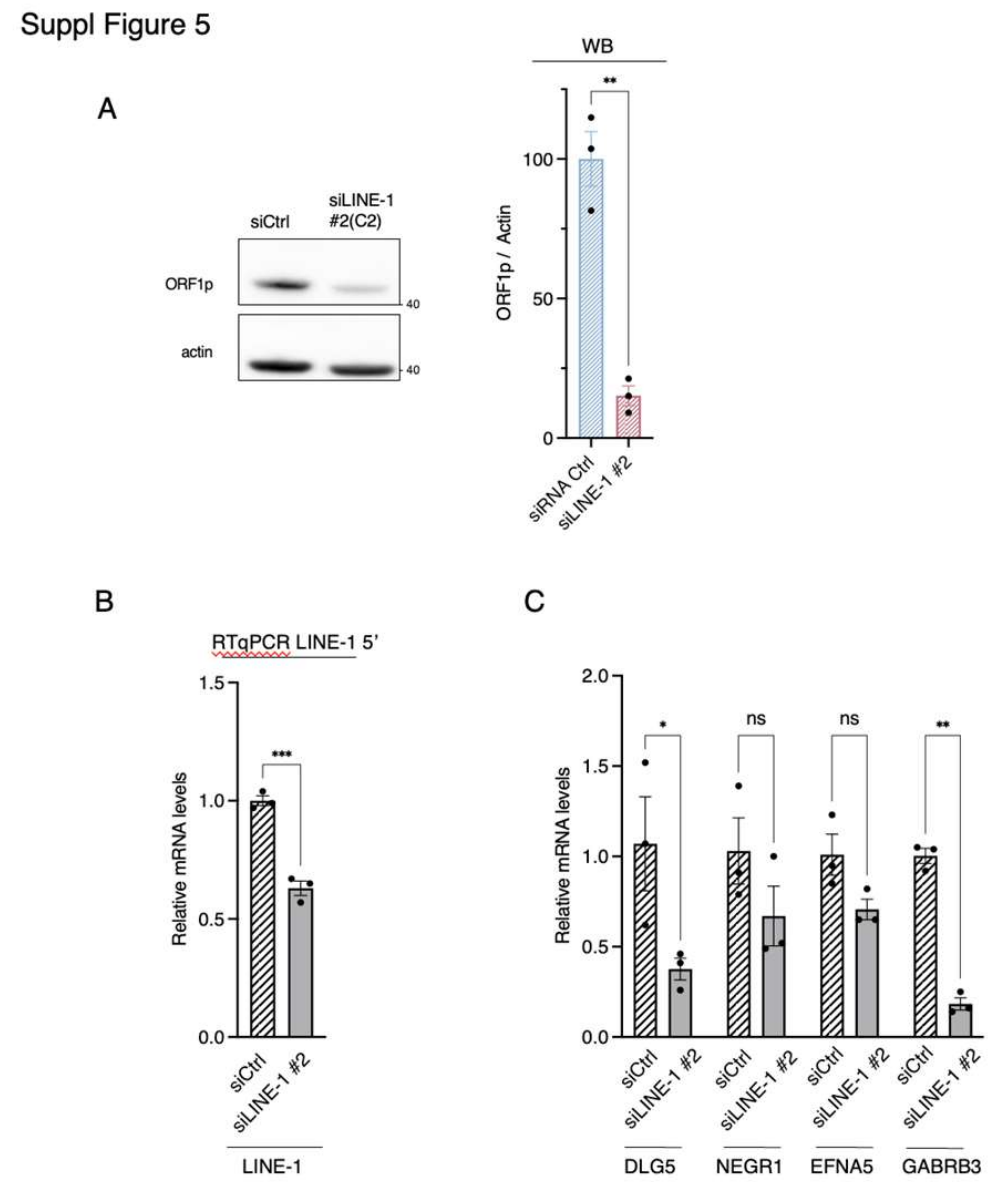

Line 467: "In order to independently validate the LINE-1 KD mediated downregulation of neuron-related genes, we used an additional siRNA targeting LINE-1. Despite difficulties in selecting siRNAs knocking down LINE-1 ORF1p in LUHMES neurons, where the ORF1p protein seems to be particularly stable, we identified another LINE-1 siRNA which knocked down ORF1p efficiently as shown and quantified in Suppl. Fig. 5A using the same protocol as above. Using this LINE-1 siRNA ((siLINE-1 #2), LINE-1 RNA was significantly downregulated (Suppl. Fig. 5B). We then tested this siRNA targeting LINE-1 in RTqPCR selecting the same four genes we had identified as downregulated in the RNA-seq experiment and in RTqPCR using siLINE-1 #1 as shown above (Fig.6). We confirmed the downregulation of DLRG5 and GABRB3, and there was a trend, but no significant downregulation, of the expression of NEGR1 and EFNA5 (Suppl. Fig. 5C)."

On-target validation

We validated on-target effects by Western blot and RTqPCR showing efficient protein and (less efficient, non significant) RNA knock-down. We have now added new data based on the RNA-seq experiment. We reanalyzed the RNA-seq data with a TE-adapted mapping method (please see Materials and Methods for details) normalized the raw counts by gene +TE counts and plotted the normalized expression of young LNE-1 families in a heatmap, now added to Suppl. Fig. 3B and below. As expected, the youngest LINE-1 families L1HS and L1PA2 are less transcribed than the older families L1PA3-5. On the right is a depiction of the data for L1HS and L1PA2 separately, in dot plot format (mean+/-SEM, n=4)). Overall, the siRNA did not downregulate LINE-1 RNA at the family level, but most probably certain L1HS/L1PA2 elements more specifically, which is why we observe a siRNA-dependent trend for downregulation when using LINE-1 primers located in the 5'UTR of young LINE-1 elements (as shown in Suppl Fig 3B).

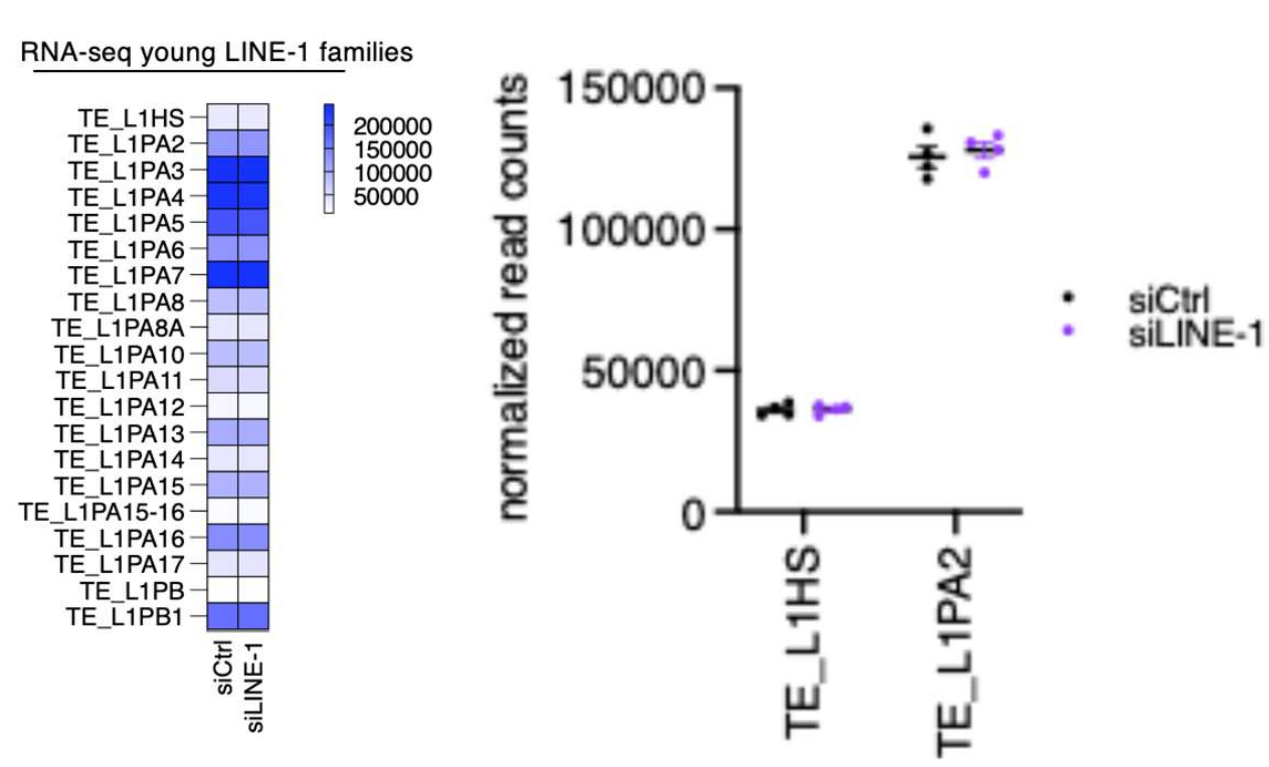

Concerning potential on and off-targets, we also did additional *in silico* analyses of the human and mouse siRNA used.
Human siRNA :
The human LINE1-ORF1 siRNA sequences we used had been evaluated by another group in a previous publication (Philippe et al, eLife, 2016). We evaluated the two sequences in this study (siLINE-1#1 and siLINE-1 #2), which the authors had successfully used as shRNA for LINE-1 KD, The first sequence which efficiently knocked-down ORF1p in our experimental system was (AAGAAGGCTTCAGACGATCAA which was adapted from the shRNA#1 used in Philippe et al, eLife, 2016 -> CCGG**AAGAAGGCTTCAGACGATCAA**CTCGAGTTGATCGTCTGAAGCCTTCTTTTTTTG with the target sequence indicated in bold). They showed that this shRNA efficiently knocked-down ORF1p (WB) and LINE-1 RNA (RNA-seq) in two different cell lines, namely MCF7 and 2102Ep (Philippe et al, eLife, 2016 -> Figure 3C, D and E). Using ClustalW alignment of all 146 full-length LINE-1 with intact open reading frames ORF1 and ORF2 identified in the LINE-1Base version 2 database (Penzkofer et al, NAR, 2017), we identified the target sequence in 134 of the 146 full-length LINE-1 sequences with intact coding potential.
On and Off-target assessment of the LINE-1 siRNA was performed *in silico* using BLASTn-short against the human genome and transcriptome. ORF1 was identified, as expected, confirming LINE-1 as the on-target. We agree that siRNA off-target effects are an important consideration. Three protein-coding transcripts (GRHL2, ZNF66 and SOD2) exhibited extended complementarity (as defined by 100% coverage and >95% complementarity) in this BLAST analysis, indicating the possibility of Ago2-mediated cleavage. We therefore analyzed the RNA-seq data in our cellular system after LINE-1 KD as to whether the expression of these three potential targets was affected. All three transcripts were not significantly regulated comparing siRNA ORF1p to scrambled siRNA with raw and adjusted p-values >0.05 and visual inspection of all loci confirmed this throughout the whole gene locus. BLAST identified nine non-coding RNAs, of which all, but one (PENK-AS1) contained a L1 PA2, PA3, PA4 or PA5 element. None of these transcripts was significantly regulated upon LINE-1 KD.

Mouse siRNA : The mouse siRNA stems from a previous publication from our group (Blaudin de Thé et al, EMBO J, 2018) where we could show, importantly along two other anti-LINE-1 strategies, that the infusion of this siRNA (coupled to penetratin) was sufficient to rescue mouse dopaminergic cell loss *in vivo* and to prevent DNA strand breaks in primary neurons *in vitro*. We have corrected in the manuscript, that this sequence is directed against ORF2 and not ORF1, our apologies for this error. Blastn optimized for short sequences did not reveal any off targets matching 100%, but did reveal one potential off-target, namely Maip1, which is a nuclear encoded mitochondrial protein involved in calcium transport (https://www.genecards.org/cgi-bin/carddisp.pl?gene=MAIP1).

2. It is also good practice to perform rescue experiments. Because the authors target repeated sequences that can also be dispersed in genes, knocking down LINE-1 may also affect genes that contain them (not strictly speaking off-target effects, but rather collateral effects). This may explain why the comparison of ORF1p high/low versus ORF1p control/knockdown yields minimal overlap and why longer genes (which have a higher probability of containing L1 sequences) are more affected by LINE-1 knockdown.

We agree, that it would have been instructive to co-transfect the ORF1p siRNA and the ORF1p-HA construct, but it is technically, in the cellular system we use, difficult to do so due to the notoriously difficult-to-transfect mature neurons. The siRNA was transfected with lipofection, but did not transfect well with nucleofection and the plasmids only transfected by nucleofection. What we could do, however, was to verify that long transcripts were affected by LINE-1 knockdown independent on the fact to contain an intronic full-length LINE-1 element. Indeed, of 68 unique genes which contain a full-length LINE-1 in one of the introns (LINE-1 Base v2, Penzkofer et al, NAR, 2017), 62 were identifiable by their gene name in the LINE-1 LOF RNA-seq data. Of these 62 genes, the vast majority (54), as expected, were long genes (>100kb). Of all downregulated transcripts, 155 were long genes. Of these only two contained an intronic full-length LINE-1 sequence (FRAS1 and NEGR1). Among upregulated genes, no transcript contained a flL1 sequence. Therefore, we conclude, that the probability to be differentially regulated by LINE-1 knock-down for a long transcript containing a flLINE-1 sequence is not higher than for long transcripts not containing a flLINE-1 sequence. Of genes shorter than 100kB, no gene shorter than 100kB and containing an intronic flLINE-1 sequence was regulated by LINE-1 KD.
We added the following sentence to the manuscript, line 443:
« However, the probability of a long gene containing a flLINE-1 sequence to be differentially regulated by LINE-1 knock-down was not higher than for long genes not containing a flLINE-1 sequence. Along the same line, 59 out of 671 downregulated genes contained >/=1 young L1HS or L1PA2 element (8,79%), a frequency similar to the 19 out of 254 upregulated genes containing >/=1 young L1HS or L1PA2 element (7,48%). «

3. Whether some of the observed effects are direct and whether they are mediated by ORF1p or LINE-1 RNA requires clarification. Therefore, the rescue could be performed with LINE-1 sequences that can or cannot be translated into ORF1p.

We agree that the issue of whether the effects are mediated by ORF1p and/or ORF1p+LINE-1 RNA remains unresolved. However, RTqPCR experiments and the newly added data showing that LINE-1 RNA in the RNA-seq experiment and in the RTqPCR, using two different siRNAs is not always significantly downregulated, suggest that in our system, the effects we observe are mediated at least in part by the knock-down of the ORF1 protein and possibly not or only partially by the knock-down of the LINE-1 RNA. However, this does not exclude that ORF1p associates with LINE-1 RNA.
To tone down, we have replaced ORF1p-LOF by LINE-1 KD. We have also made other changes throughout the manuscript :
Line 699: « Although we have provided correlative genomic and loss of function evidence for an involvement of ORF1p in adult neuronal physiology, our study has some limitations. We cannot definitively exclude that the observed effects are partially mediated by LINE-1 RNA or other indirect effects or whether ORF1p is bound to LINE-1 RNA on chromatin. The identification of ORF1p chromatin binding sites, the characterization of the ORF1p complex binding chromatin and/or rescue experiments of ORF1p should be the subject of future studies."
Line 349: "Although effect sizes were rather low, we observed…."
Line 622: "Whether once in the nucleus, ORF1p associates with RNA or not to bind chromatin, is an open question, which remains to be addressed."
Line 640: "Whether LINE-1 RNA might be involved or not remains ot be investigated. »
Line 643:" One possibility is through the association with LINE-1 RNA or directly with proteins involved in chromatin-related functions."
Line 502: "LINE-1 knock-down leads to changes in neurites morphology"
"In addition to the fact that knock-down of LINE-1 induced chromatin accessibility and transcriptomic changes which suggested that LINE-1 ORF1p (+/-LINE-1 RNA) might be involved in regulating the homeostasis and maintenance of adult neurons at steady-state."
Line 545:" In summary, these data suggest that LINE-1 ORF1p, in conjunction or not with LINE-1 RNA, plays a role in the maintenance of neurite morphology possibly by converging pathways mediated by protein interactions and by modulating chromatin accessibility and transcriptomic landscapes."

Minor points:
4. It would be helpful to clarify whether, in this experimental system, ORF1p binding to chromatin is RNA-dependent and whether ORF1p can directly bind DNA (ChIP-seq).

Indeed, we cannot be sure that the effects are solely protein and not RNA-dependent. Indeed, LINE-1 RNA revealed by RNAscope seems detectable only in the nucleus (preliminary data). However, both in the RNA-seq data as well as in independent RTqPCR experiments, LINE1 RNA content overall did not seem to be systematically affected by the LINE-1 siRNA. This indeed suggests that what we observe is at least not majorly driven by L1 RNA-dependent changes and reinforces the notion of ORF1 protein-dependent mechanisms especially when taking into account the detectable decrease of ORF1p on chromatin after LINE1-KD (Suppl. Fig. 3C).
In the case of human brain nuclei containing high or low ORF1p, we do not know whether ORF1p is bound or not to LINE-1 RNA. Importantly, whether we characterize accessible chromatin features in human brain or in human neurons *in vitro*, we do observe similar overall characteristics depending on the content of ORF1p in the nucleus/cells (please see also table 3). This does suggest that in both systems, the fact of having high or low ORF1p content, is associated with similar chromatin accessibility changes and indicates at least that these effects are protein-dependent and may or may not be RNA-dependent.

5. Subcellular fractionation experiments would benefit from quantification. Some fractions are missing in Fig. S3C.

In subcellular quantification experiments shown in Fig 2, we are not drawing any quantitative conclusions and therefore, we believe that quantifications are not necessary or even useful. We agree that in S3C, quantifications comparing siCtrl and siLINE-1 are useful, which is why we added the quantification to the manuscript (Suppl. Figure 3C, see below). Indeed, there is no PE compartment (= pellet) in the LINE1-KD condition on the membrane simply because there was no more space available (the 10th lane or rather the first one was reserved for the molecular weight ladder). However, the pellet fraction might not be indispensable for the conclusion of a lowering of all fractions per marker in the siLINE-1 condition compared to the siCtrl as now quantified and added to Suppl Fig 3C.

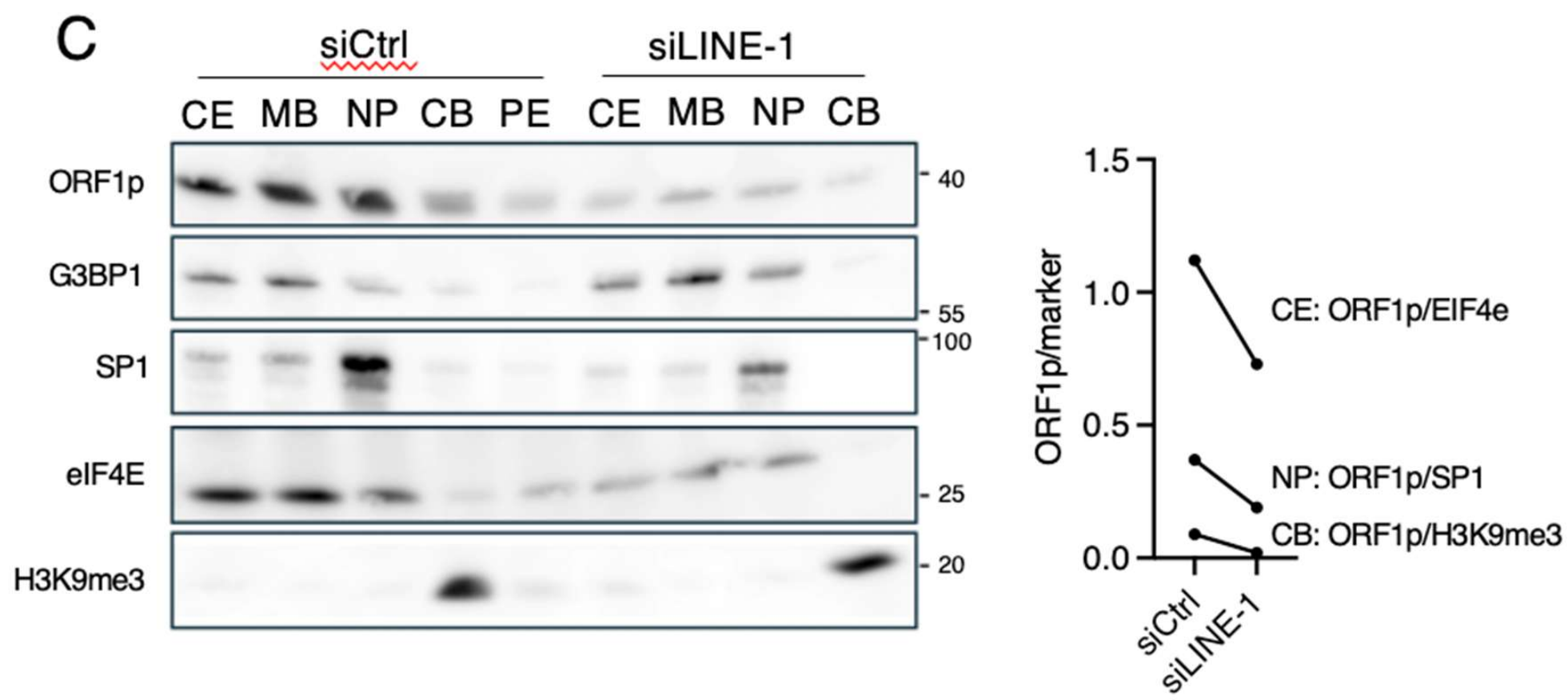

6. Do differentially expressed genes upon LINE-1 knockdown contain L1 sequences and/or potential binding sites for the LINE-1 siRNA?

We have not identified genes which contain a potential siRNA binding site to be downregulated (please also see above). We have also checked whether downregulated genes contained young LINE-1 sequences (L1HS or L1PA2). The human genome (hg38) contains 1725 L1HS and 5251 L1PA2 elements (please find details in the Materials and Methods section). We overlapped the list of genomic intervals of differentially regulated RNAs from the RNA-seq experiment upon LINE1-KD with the intervals of all L1HS and L1PA2 elements. Of 671 downregulated genes, 59 genes (8,79%) contain >/= 1 L1HS or L1PA2. Of 254 upregulated genes, 19 genes (7,48%) contain >/=1 L1HS or L1PA2. We conclude that there are not more young LINE-1 elements in downregulated compared to upregulated genes upon LINE1-KD.
We added the following sentences to the manuscript, line 443: « However, the probability of a long gene containing a flLINE-1 sequence to be differentially regulated by LINE-1 knock-down was not higher than for long genes not containing a flLINE-1 sequence. Along the same line, 59 out of 671 downregulated genes contained >/=1 young L1HS or L1PA2 element (8,79%), a frequency similar to the 19 out of 254 upregulated genes containing >/=1 young L1HS or L1PA2 element (7,48%). »

7. When performing knockdown of LINE-1, there is a minimal effect on LINE-1 RNA by RT-qPCR (Fig. S3B). What about separating cytoplasmic versus nuclear RNA? It would be helpful to understand which fraction of LINE-1 RNA is actually affected by the knockdown.

In preliminary LINE-1 RNAscope experiments (in LUHMES), we observe LINE-1 RNA only in the nucleus.

8. Fig. S3 B and C: legends were inverted.

Thank you for pointing this out, this has been corrected.

9. Fig. S3D does not show DAR−/DAR+ unlike the main text description (lines 360-364).

Thank you, this has been corrected.

10. Fig. 4E/F: track legends (on the left) do not correspond to the displayed tracks.

Thank you, this has been corrected.

11. Referencing: in the introduction, the original primary papers showing LINE-1 expression in the brain are missing (from the Gage and Faulkner labs). Most cited preprints are now published (Zernia et al., Sci. Adv. 2025; Adami et al., Cell Genom. 2025; Bonnifet et al., eLife 2025; Zhang et al., Dev. Cell 2025) and could be referenced as such.

Yes of course, this has been added/changed. One paper is still in preprint form : Pérez-Rico YA, Bousard A, Misikova LH, et al. Transcriptional perturbation of LINE-1 elements reveals their cis -regulatory potential. Genomics. Preprint posted online February 20, 2024. doi:10.1101/2024.02.20.581275. All other papers have been added or their published version is now cited.

12. The article by Della Valle et al., Sci. Transl. Med. 2022, which describes the role of LINE-1 RNA in heterochromatin formation, notably through SUV39H1 regulation, is directly related to the present study and would merit citation and discussion.
We apologize for the omission to cite this paper, which indeed is very relevant for our manuscript. We have now discussed and cited this work in the discussion:
Line 572: «In premature aging cells, LINE-1 is upregulated and LINE-1 RNA acts a negative regulator of the heterochromatin maintenance enzyme SUV39H1 (suppression of variegation 3-9 homolog 1). SUV39H1 dysfunction leads to heterochromatin loss and onset of aging phenotypes which can be rescued by ASO-mediated depletion of LINE-1 RNA (Del Valle et al, Sci Transl Med, 2022)."

13. Although this could be due to the submission system, the quality of network graphs could be improved, as they are not readily readable in their present state.

We have improved the visual aspect of the genome browser snapshots.

14. Comparison of IP/MS between mouse and human, and with previous ORF1p IP/MS obtained in other systems, could be enhanced in the supplementary table: which interactor was found in which study/sample? (Adding a column per cell type and/or study, rather than a simple list of "known interactors," would facilitate comparison).

We have now added each study in separate columns to Suppl Table 1 (known ORF1p interactors MN9D or LUHMES), which indeed facilitates greatly the comparison between the different studies.

Thank you very much for your comments and suggestions.

July 10, 2026

RE: Life Science Alliance Manuscript #LSA-2025-03546-TR

Dr. Julia FUCHS
Collège de France
Centre for Interdisciplinary Research in Biology (CIRB)
11, place Marcelin Berthelot
Paris 75231 Paris Cedex
France

Dear Dr. Fuchs,

Thank you for submitting your revised manuscript entitled "Interactions of LINE-1 ORF1p with proteins and chromatin converge on a role in neuronal physiology".

Your revised manuscript has been assessed by the original reviewers whose notes are appended below. As you will note, all the reviewers have acknowledged the potential value of this work. Reviewers 2 and 3 also noted that their concerns were not resolved entirely. That said, all the reviewers have acknowledged the challenge in resolving all the concerns and noted your moderated conclusions. We hope you find the additional suggestion and clarification from Reviewer 1 useful for your future work.

In line with the reviewers' overall evaluation, we would be happy to publish your paper in Life Science Alliance pending resolution of final revisions necessary to meet our formatting guidelines.

MANUSCRIPT ORGANIZATION AND FORMATTING:

To avoid unnecessary delays in the acceptance and publication of your paper, please read the following information carefully. Full guidelines are available on our Instructions for Authors page, https://www.life-science-alliance.org/authors

Submission of a paper that does not conform to Life Science Alliance guidelines will delay the acceptance of your manuscript.

-We recommend a minor edit in your title (should you agree to change it): "Interactions of LINE-1 ORF1p with proteins and chromatin suggest a role in neuronal physiology".
-Please see the point of Reviewer 1 on higher resolution images for Figure 1E and 1F.
-Please include a scale bar and size information for Figure 7F and recommend that you make the existing scale bars in Figure 7 more prominent. Please include scale bar and size information for S1C.
-For Figure 1A, for both images of the blots, please remove the lane with the molecular weight marker whilst indicating the molecular weight (as done for Figure 2).
-Please include a statement for experiments involving human subjects that informed consent was obtained from all subjects and that the experiments conformed to the principles set out in the WMA Declaration of Helsinki and the Department of Health and Human Services Belmont Report.
-Kindly expand on imaging details for information on objectives and fluoresce imaging.
-Please include antibody names, source and concentrations in description of methods for western blotting and imaging experiments. Please include concentrations of antibodies used in FACS experiments. If you prefer, you may also include a table with all this information and refer to the table in the methods section.
-Please add ORCID ID for corresponding (and secondary corresponding) author - you should have received instructions on how to do so.
-Please add the X and Bluesky handles of your host institute/organization, as well as your own, and/or one of the authors, in our system.
-Please rename "Competing interests" to "Conflict of Interest."
-Please add your main, supplementary figure, and table legends to the main manuscript text after the references section.
-The "Data Availability" section should be placed after the Materials & Methods section. Please consult our guidelines at https://www.life-science-alliance.org/manuscript-prep#format.
-Figure 5 has only panels A and B; however, there is a call-out for Figure 5C in the manuscript text. Please correct this.
-Please be sure that the authorship listing and order is correct

We encourage our authors to provide original source data, particularly uncropped/-processed electrophoretic blots and spreadsheets for the main figures of the manuscript. If you would like to add source data, we would welcome one PDF/Excel-file per figure for this information. These files will be linked as supplementary "Source Data" files.

It is Life Science Alliance policy that if requested, original data images must be made available to the editors. Failure to provide original images upon request will result in unavoidable delays in publication. Please ensure that you have access to all original data images prior to final submission.

If you are planning a press release on your work, please inform us immediately to allow informing our production team and scheduling a release date.

We welcome submissions of potential cover images for the issue of LSA in which your work would appear. If you have high quality images associated with this work, please feel free to email these, with a caption, to the journal office.

LSA encourages authors to provide a 30-60 second video where the study is briefly explained. These videos will be appear embedded with the manuscript online at Life Science Alliance, and on social media to promote the published paper and authors (for examples, see https://docs.google.com/document/d/1-UWCfbE4pGcDdcgzcmiuJl2XMBJnxKYeqRvLLrLSo8s/edit?usp=sharing). Corresponding or first-authors are welcome to submit the video. Please submit only one video per manuscript. The video can be emailed to contact@life-science-alliance.org

FINAL FILES:

To upload the final version of your manuscript, please log in to your account: https://lsa.msubmit.net/cgi-bin/main.plex
You will be guided to complete the submission of your revised manuscript and to fill in all necessary information. Please get in touch in case you do not know or remember your login name.

The following items are required for acceptance.

-- An editable version of the final text (.DOC or .DOCX) is needed for copyediting (no PDFs).

-- High-resolution figure, supplementary figure and video files uploaded as individual files: See our detailed guidelines for preparing your production-ready images, https://www.life-science-alliance.org/authors

-- Summary blurb (enter in submission system): A short text summarizing in a single sentence the study (max. 200 characters including spaces). This text is used in conjunction with the titles of papers, hence should be informative and complementary to the title. It should describe the context and significance of the findings for a general readership; it should be written in the present tense and refer to the work in the third person. Author names should not be mentioned.

The license to publish form must be signed before your manuscript can be sent to production. A link to the license to publish form will be available to the corresponding author only. Please take a moment to check your funder requirements.

Reviews, decision letters, and point-by-point responses associated with peer-review at Life Science Alliance will be published, alongside the manuscript. If you do want to opt out of having the reviewer reports and your point-by-point responses displayed, please let us know immediately.

Thank you for your attention to these final processing requirements. Please revise and format the manuscript and upload materials as soon as you are able.

Thank you for this interesting contribution to the literature. We look forward to publishing your paper in Life Science Alliance.

Sincerely,

Sarita Hebbar, PhD
Scientific Editor
Life Science Alliance
http://www.lsajournal.org

Reviewer #1 (Comments to the Authors (Required)):

In this revision the authors added a second siRNA to support their claims and tried to address the key overarching critique of all three reviewers about direct/indirect effects and on/off targets of their interventions. Here are some comments:

-I think they misunderstood my comment about L1-high and L1-low DARs: they say they found only one DAR overlapping a young L1 and conclude that ORF1 doesn't regulate accessibility at L1 promoters. My comment was different. I was trying to say that there should be DARs at L1 promoters *because L1s are differentially expressed in L1-high vs L1-low neurons*. The protein has to come from transcript...so it would surely be expected that L1s are more transcribed in one condition than another, and it

therefore stands to reason that L1 promoters of whichever elements make the protein should therefore be differentially accessible. It could be that the L1 protein comes from only one or two elements - maybe this one in XXYLT1! That one is transcribed in fetal brain, see fig4b PMID 37910626 (or it could be polymorphisms absent from the hg38 reference, or in unmappable/blacklisted/masked regions). Noone actually knows the answer to this question I think... This is what I'm getting at, and another reason that the L1-high vs L1-low analysis is quite exciting regardless of the putative ORF1-protein-dependent effects. I hope this makes sense.

-This serves as an example of just how challenging this topic is. These questions are just really technically demanding to answer definitively, and while I do applaud the efforts put in by the authors, I do remain a little skeptical of some of the KD findings. I would definitely suggest for future studies to use a technology to directly manipulate ORF1 at the protein level, and to make further efforts on the ChIP (or similar) experiments. I am glad they have toned down the text in several places, and would suggest to clearly discuss the challenges and caveats in the conclusions of the paper.

-I still can't read the protein network graphs of Fig 1!

My overall suggestion is to publish the study because it is interesting and the Fig1,2,3 present data that could be valuable to others pursuing these ideas. Also, while I am still a bit skeptical about the L1 KD on/off targets, I feel the paper is honestly presented and could provoke valuable discourse and challenge in the L1 community. To conclusively nail the underlying biochemistry and mechanisms here will probably be a whole new study, and I wish the authors luck in this!

Christopher Douse
Lund Stem Cell Center

Reviewer #2 (Comments to the Authors (Required)):

The authors have addressed the points I requested. Most of them could not be resolved due to technical limitations and sensitivity issues. Based on this, they have toned down their statement. Although the project did not provide new mechanistic insights, the study revealed new potential regarding the role of LINE-1 Orf1, so the project will provide the specific research community with some new information.

Reviewer #3 (Comments to the Authors (Required)):

A major issue raised by all three reviewers (the use of a single siRNA against L1) has been partially addressed: the authors identified a second effective siRNA and confirmed that the 4 genes downregulated by the first siRNA are also downregulated by the second. I regret that the authors chose this minimal validation rather than a full RNA-seq comparison of gene expression profiles. This does not exclude the possibility that the observed effects could stem from downregulation of a pre-mRNA containing a bona fide intronic L1 target (one of the 4 genes is NEGR1, which does contain a full-length L1, as explained by the authors). However, given the significant rewording and toning down of the manuscript, I will refrain from any additional comments and do not see major issues that would prevent publication in Life Science Alliance.

August 11, 2026

RE: Life Science Alliance Manuscript #LSA-2025-03546-TRR

Dr. Julia FUCHS
Collège de France
Centre for Interdisciplinary Research in Biology (CIRB)
11, place Marcelin Berthelot
Paris 75231 Paris Cedex
France

Dear Dr. Fuchs,

Thank you for submitting your Research Article entitled "Interactions of LINE-1 ORF1p with proteins and chromatin suggest a role in neuronal physiology". It is a pleasure to let you know that your manuscript is now accepted for publication in Life Science Alliance. Congratulations on this interesting work.

The final published version of your manuscript will be deposited by us to PubMed Central upon publication.

Your manuscript will now progress through copyediting and proofing.
We noted that your statement on availability of ATAC-seq data from human tissue is only available upon request from the authors. We understand that these data may not be publicly available due to privacy or ethical restrictions. We invite you to clarify this in your statement, which you may change during the proofing stage as follows: "The ATAC-seq data stemming from post-mortem human brain tissue is only available upon request from the authors due to privacy and ethical restrictions."

It is journal policy that authors provide original data upon request. Your article will publish open access upon publication under a CC-BY license.

Reviews, decision letters, and point-by-point responses associated with peer-review at Life Science Alliance will be published, alongside the manuscript. If you do want to opt out of having the reviewer reports and your point-by-point responses displayed, please let us know immediately.

IMPORTANT: If you will be unreachable at any time, please provide us with the email address of an alternate author. Failure to respond to routine queries may lead to unavoidable delays in publication.

Scheduling details will be available from our production department. You will receive proofs shortly before the publication date. Only essential corrections can be made at the proof stage so if there are any minor final changes you wish to make to the manuscript, please let the journal office know now.

DISTRIBUTION OF MATERIALS:
Authors are required to distribute freely any materials used in experiments published in Life Science Alliance. Authors are encouraged to deposit materials used in their studies to the appropriate repositories for distribution to researchers.

You can contact the journal office with any questions, contact@life-science-alliance.org

Again, congratulations on a very nice paper. I hope you found the review process to be constructive and are pleased with how the manuscript was handled editorially. We look forward to future exciting submissions from your lab.

Sincerely,

Sarita Hebbar, PhD
Scientific Editor
Life Science Alliance
http://www.lsajournal.org
