# Supplementary material for: Interactions of LINE-1 ORF1p with proteins and chromatin suggest a role in neuronal physiology
Source: Life Sci Alliance. 2026 Aug 21;9(11):e202503546. doi: 10.26508/lsa.202503546 (PMC13498733; doi:10.26508/lsa.202503546)
Supplement: Supplementary file 2 [file LSA-2025-03546_TableS3.docx]

**Table 2.** Metadata of post-mortem cingulate gyrus tissues used in FANS/ATAC-seq

PD: Parkinson disease

PMI: post-mortem interval
